# Publisher Correction: Terahertz field effect in a two-dimensional semiconductor

**DOI:** 10.1038/s41467-026-68521-1

**Published:** 2026-01-17

**Authors:** Tomoki Hiraoka, Sandra Nestler, Wentao Zhang, Simon Rossel, Hassan A. Hafez, Savio Fabretti, Heike Schlörb, Andy Thomas, Dmitry Turchinovich

**Affiliations:** 1 Fakultät für Physik, Universität Bielefeld, Bielefeld, Germany; 2 Leibniz-Institut für Festkörper- und Werkstoffforschung, Helmholtzstraße 20, Dresden, Germany; 3 Institut für Festkörper- und Materialphysik, Technische Universität Dresden, Haeckelstraße 3, Dresden, Germany

**Keywords:** Terahertz optics, Two-dimensional materials, Electronic devices

Correction to: *Nature Communications* 10.1038/s41467-025-60588-6, published online 05 June 2025

In the version of the article initially published, “$\left|{F}_{x,{{{\rm{in}}}}}\right|=10\,{{{\rm{kV}}}}/{{{\rm{cm}}}}$” in the “THz pump-optical probe measurements” section and “${F}_{x,{{{\rm{in}}}}}=10\,{{{\rm{kV}}}}/{{{\rm{cm}}}}$” in the caption to Fig. 3d should have read “$\left|{F}_{x,{{{\rm{in}}}}}\right|=104\,{{{\rm{kV}}}}/{{{\rm{cm}}}}$” and “${F}_{x,{{{\rm{in}}}}}=104\,{{{\rm{kV}}}}/{{{\rm{cm}}}}$”, respectively. This correction has been made to the HTML and PDF versions of the article.

