# Supplementary material for: Terahertz field effect in a two-dimensional semiconductor
Source: Nat Commun. 2025 Jun 5;16:5235. doi: 10.1038/s41467-025-60588-6 (PMC12141635; doi:10.1038/s41467-025-60588-6)
Supplement: Supplementary file 1 — Supplementary Information [file 41467_2025_60588_MOESM1_ESM.pdf]

Supplementary Information

Terahertz field effect in a two-dimensional semiconductor

Tomoki Hiraoka[1], Sandra Nestler[2], Wentao Zhang[1], Simon Rossel[1], Hassan A. Hafez[1], Savio Fabretti[1], Heike Schlörb[2], Andy Thomas[2,3], and Dmitry Turchinovich[1]

[1]Fakultät für Physik, Universität Bielefeld, 33615 Bielefeld, Germany
[2]Leibniz-Institut für Festkörper- und Werkstoffforschung, Helmholtzstraße 20, 01069 Dresden, Germany
[3]Institut für Festkörper- und Materialphysik, Technische Universität Dresden, Haeckelstraße 3, 01069 Dresden, Germany

Contents

Supplementary Notes 1. Simulation of THz-field response of 2D-3D hybrid THz antenna

1-1. Dimensions of 2D-3D hybrid THz antenna

1-2. THz conductivity of 8-nm-thick Au film

1-3. FDTD simulation setting

1-4. Convergence test

1-5. FDTD simulation results: Field enhancement

1-6. FDTD simulation results: Spatial inhomogeneity

Supplementary Notes 2. Simulation of optical reflectance spectra

2-1. Reflectance spectra of antenna without MoS_2 flake

2-2. Reflectance spectra of antenna with MoS_2 flake

2-3. Origin of reflectance dip shift

2-4. Injecting carrier to 2D material via direct contact to an electrode

Supplementary Notes 3. Chirp correction of TPOP measurement data

Supplementary Notes 4. Adjustment of time offset of EO-sampling and TPOP-measurement data

Supplementary Notes 5. DC biasing

5-1. Leak current and dielectric-breakdown threshold measurement

5-2. Unexpected leak current path

Supplementary Notes 6. All the TPOP measurement and analysis data

Supplementary Notes 7. Photoluminescence and reflectance measurement data

Supplementary Notes 8. Optical measurement detail

8-1. Reflection microspectroscopy

8-2. THz pump-optical probe reflection microspectroscopy

8-3. EO sampling

Supplementary References

Supplementary Notes 1. Simulation of THz-field response of 2D-3D hybrid THz antenna

We have simulated the THz-field response of the hybrid 2D-3D antenna structure with a finite difference time domain (FDTD) method. Supplementary Notes 1-1 shows the actual dimensions of the antenna structure, which was also used for the simulation. Supplementary Notes 1-2 shows the characterization of THz-conductivity of 8-nm-thick gold film, which was necessary for the simulation. Supplementary Notes 1-3, 1-4, and 1-5 show the settings, convergence test, and result of the simulation, respectively.

1-1. Dimensions of 2D-3D hybrid THz antenna

Supplementary Figure 1 shows the whole device structure with information on the thickness of the layers. Supplementary Figure 2 and 3 show the in-plane dimensions of the bottom and top electrodes, respectively.

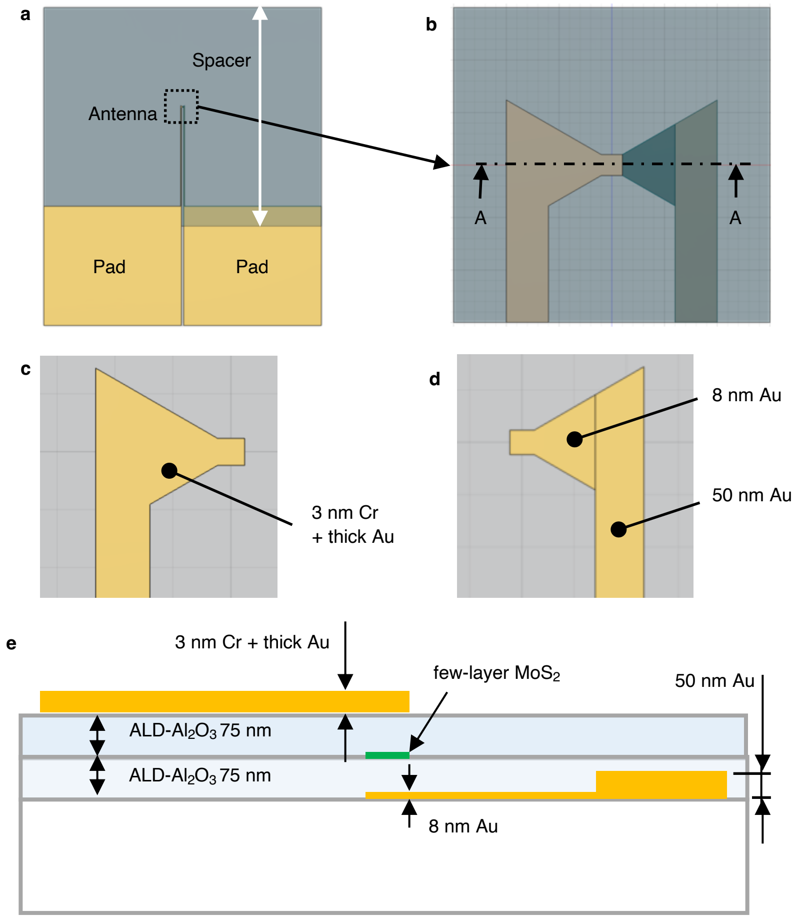

Supplementary Figure 1. The whole device structure with information on the thickness of layers. **a,** Top view of the entire structure on the substrate. The two parts noted as Pad are the contact pads for DC-biasing. **b,** Enlarged view of the antenna. The top and bottom electrodes are connected to the contact pads via connection lines. **c,** Enlarged view of the top electrode only. The thickness of the top electrode is 25 nm for α-batch and 50 nm for β-batch devices, respectively. **d,** Enlarged view of the bottom electrode only. **e,** A-A section of the device with information on thickness of the layers.

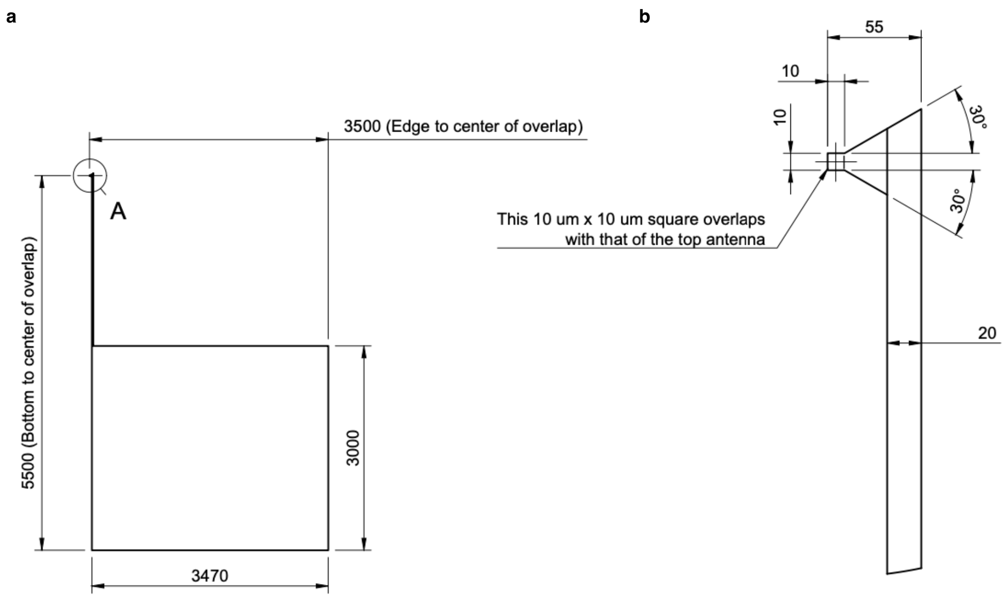

Supplementary Figure 2. In-plane dimension of the bottom electrode. **a**, Whole structure. **b**, Enlarged view of A in **a**. The unit of dimensions is μm.

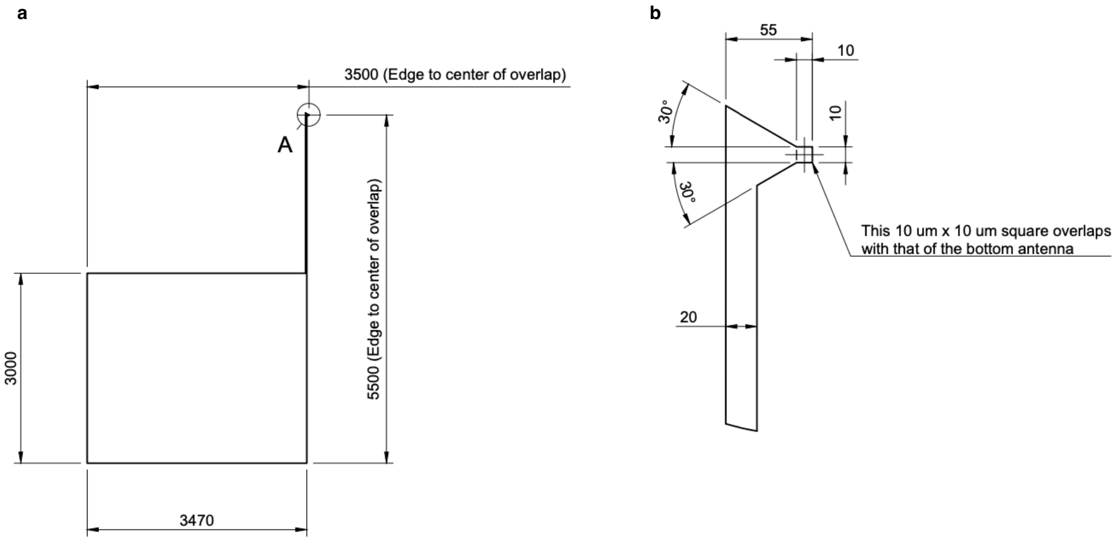

Supplementary Figure 3. In-plane dimension of the top electrode. **a**, Whole structure. **b**, Enlarged view of A in **a**. The unit of dimensions is μm.

1-2. THz conductivity of 8-nm-thick Au film

To measure the conductivity of 8-nm-thick Au film, we performed the THz time-domain spectroscopy (THz-TDS [1] on a uniform 8-nm-thick Au film sputtered on the corning Eagle XG, the glass substrate which is used for actual devices. In this measurement, we measured THz-field transmission through a glass substrate with 8-nm-thick Au film and a reference glass substrate, as shown in Supplementary Figure 4.

The conductivity spectrum was derived with an assumption that the 8-nm-thick Au film is metallic in the following way: We used Tinkham's formula [2], which gives the complex conductivity spectra of conductive thin film $\tilde{\sigma}(\omega) = \sigma_1(\omega) + i\sigma_2(\omega)$ from the complex field transmission spectra $\tilde{t}(\omega) = E_{\mathrm{Substrate+film}}(\omega)/E_{\mathrm{substrate}}(\omega)$ as

$$\tilde{\sigma}(\omega) = \frac{1+n}{Z_0 d}\left(\frac{1}{\tilde{t}(\omega)} - 1\right). \quad \text{(A1)}$$

To relate the complex transmission spectra to the complex conductivity, we need both the amplitude and phase of the transmission spectra. However, in this measurement, we could not obtain the phase of transmission spectra due to the large uncertainty of the thickness of substrates for sample and reference (tolerance of the substrate thickness was ±20 μm). To overcome this point, we used the approximation of the Hagen-Rubens regime [3], which is valid when the film thickness is much smaller than the skin depth. Since the skin depth of the gold in the THz frequency range is approximately 100 nm, the 8-nm-thick Au film is in this regime, where the real conductivity is nearly constant, and the imaginary conductivity is negligible. Then, we can relate the amplitude transmission to the real conductivity as

$$\sigma_1(\omega) = \frac{1+n}{Z_0 d}\left(\frac{1}{|\tilde{t}(\omega)|} - 1\right). \quad (A2)$$

By using eq. (A2), we derived the real conductivity of the 8-nm-thick Au film as $1.597 \times 10^7 \pm 5 \times 10^4/\Omega\mathrm{m}$, as shown in Supplementary Figure 5. This value is in the same order as that of the conductive Au nanofilms in a previous study [4], which also does not contradict the assumption of the conductive film and Hagen-Rubens regime.

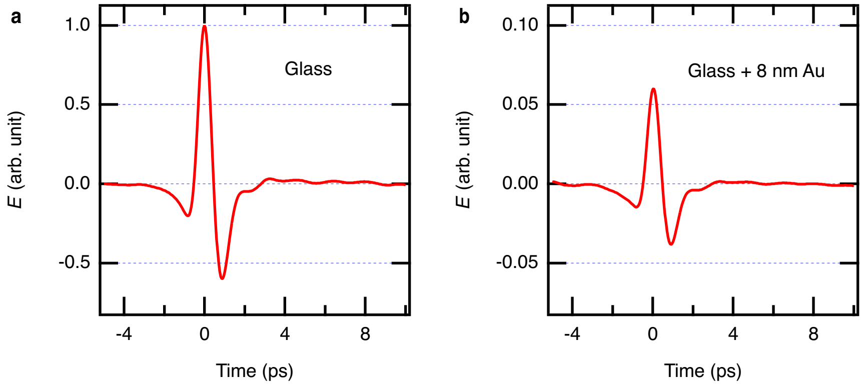

Supplementary Figure 4. Measured THz field waveforms. **a**, THz field through the reference glass substrate. **b**, THz field through the glass substrate with 8-nm-thick Au film.

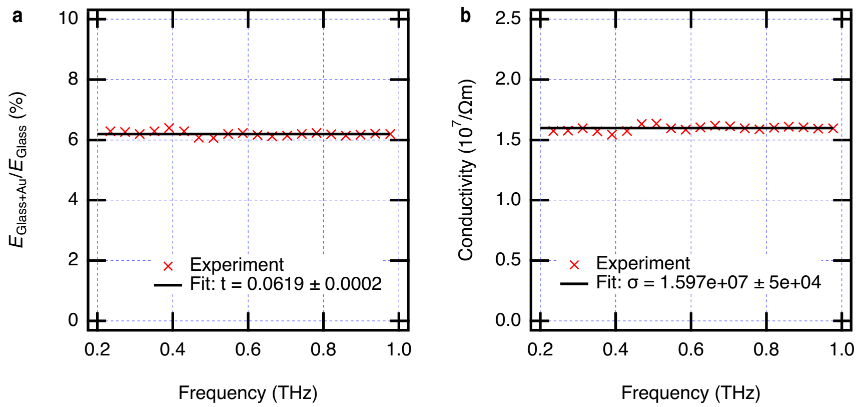

Supplementary Figure 5. Obtained spectra from THz-TDS measurement of 8-nm-thick Au film. **a**, Field-transmission spectrum. **b**, Conductivity spectrum derived from **a** via Tinkham equation and Drude model.

1-3. FDTD simulation setting

For the FDTD simulation, we used the commercial software Ansys Lumerical 2024 R2.2 Finite Difference IDE. We simulated the entire device structure without MoS_2 with the dimension shown in Supplementary Notes 1-1. The properties of materials were given as shown in Supplementary Table 1. The refractive index of the glass substrate in the THz frequency range was measured via THz-TDS. The conductivity of 8-nm-thick Au film was obtained via THz-TDS, as shown in Supplementary Notes 1-2, and was used as the conductivity of the entire top and bottom electrodes. The refractive index of ALD-grown Al_2O_3 was obtained from literature as a square root of its dielectric constant [5].

Supplementary Figure 6a and b show the entire simulation domain, location of the device and the field monitor, and location of the incident beam source. The center of the field-enhancement region of the device is set as the origin of the coordinate in this simulation, and the THz-field waveforms were monitored at this point. All the boundaries of the simulation domain are perfectly matched layers (PML). The boundaries are far enough from the origin to eliminate the simulation artifact around the PML. Also, it is far enough to delay the reflected wave at the simulation boundaries, which occurs even with the PML boundaries, returning to the monitor. Supplementary Figure 6 c and d show the close-in antenna structure and the domains with additional finer mesh settings. The details of the mesh settings are discussed in the next section.

The incident THz wave source is a Gaussian beam source with a 1/e full width (in intensity) of 1.4 mm located 600 μm above the origin and focused to the origin, as shown in Supplementary Figure 6b and Supplementary Figure 7. Its waveform is made from the actual pump THz waveform in our experiment, which was measured via EO sampling [6]. To eliminate a simulation artifact caused by the incident waveform, we made the incident waveform for the simulation by subtracting a DC offset from the measured waveform and moderating the signal cutoff via a Hanning window, as shown in Supplementary Figure 8.

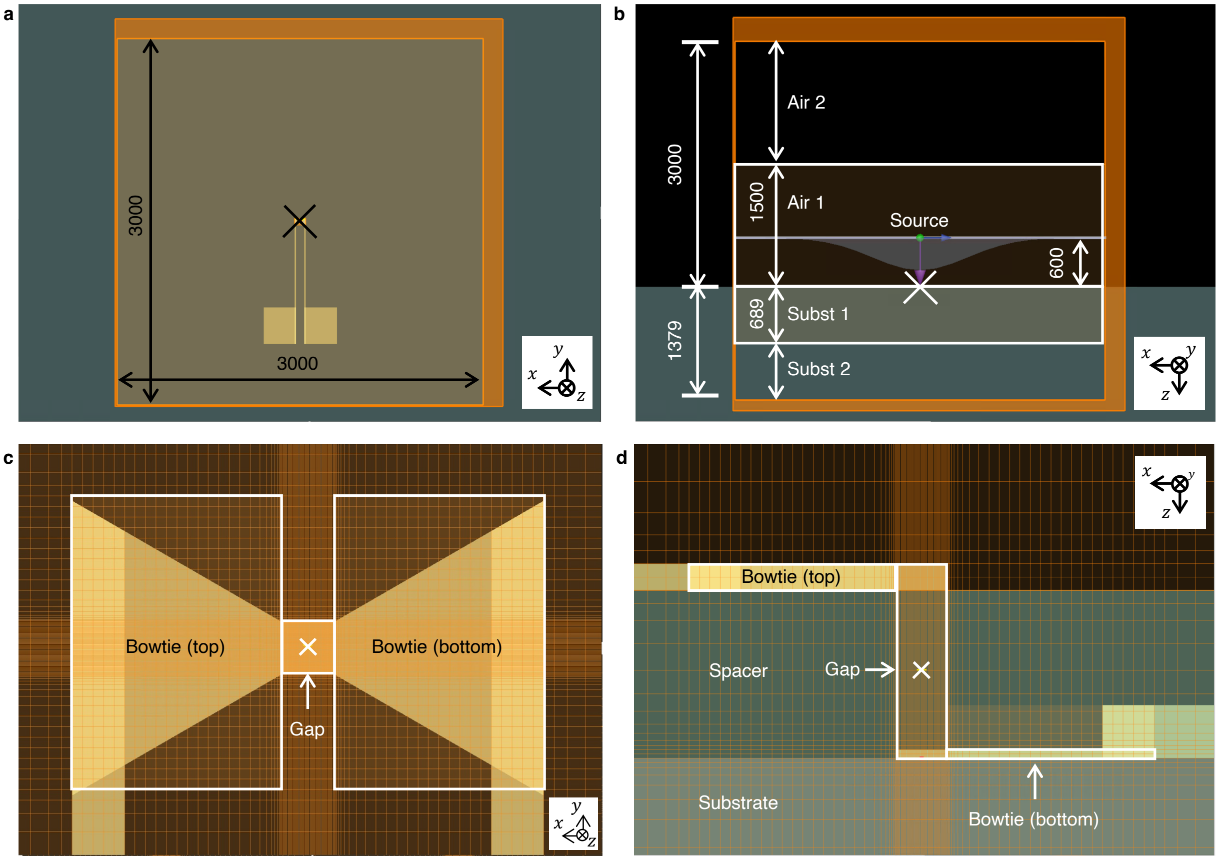

Supplementary Figure 6. Entire simulation domain and domains with additional finer mesh settings. The cross marks are the origin of the coordinate where the THz waveforms are recorded using an electric field monitor. The white lines illustrate the domains with additional finer mesh settings. The unit of length is µm. **a**, An xy-view of the entire simulation domain. The orange-colored squared domain of 3000 µm × 3000 µm is the entire simulation domain. PML boundary conditions are applied to all boundaries. **b**, An xz-view of the entire simulation domain. The extent of simulation domain in z-direction is 3000 and 1379 µm in the air and substrate, respectively. A Gaussian beam source centered at (0 µm, 0 µm, 600 µm) with the focus at the center of the antenna gap (0 µm, 0 µm, 0 µm) is used as the incident beam. Additional finer mesh settings were applied in Air 1 and Subst 1. Air 2 and Subst 2 are additionally added space to delay back reflected waves at the simulation boundary coming back to the field monitor. The mesh sizes of these domains were not specified and automatically set by the simulation software. These domains were omitted in the simulations where we do not need to monitor the waveform after 15 ps. **c**, An xy-view of the close-in antenna structure and the domains with additional finer mesh settings on the antenna: Bowtie (top), Bowtie (bottom), and Gap. **d**, An xz-view of the close-in antenna structure and the domains with additional finer mesh settings on the antenna.

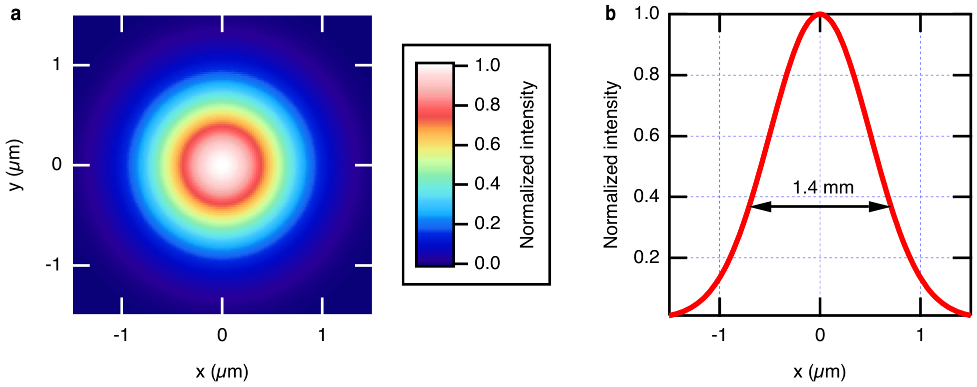

Supplementary Figure 7. Spatial distribution of the Gaussian beam source at the source plane $z = 600$ μm. **a**, Color plot of the beam intensity. **b**, Section of the beam distribution at y = 0 μm. The 1/e full width is 1.4 mm in the intensity.

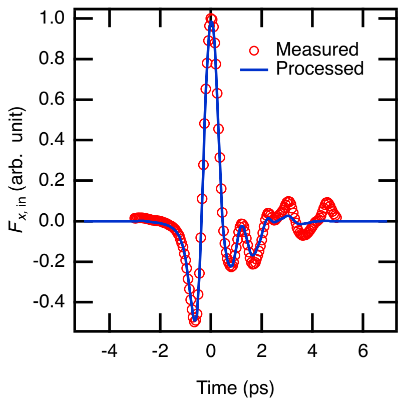

Supplementary Figure 8. Experimentally measured THz waveform and preprocessed THz waveform used as the incident wave in the simulation.

Supplementary Table 1. Properties of materials in the THz frequency domain used in the FDTD simulation.

	Material	Model	Reflactive index	Conductivity ($/\Omega$m)
Background	Air	Dielectric	1	-
Substrate	Glass	Dielectric	2.176	-
Electrodes	Au thin film	Metal	-	1.597×10^7
Spacer layer	ALD-grown Al_2O_3	Dielectric	3	-

1-4. Convergence test

In FDTD simulation, the simulated waveform gets altered in shape and amplitude if we do not use appropriate mesh settings. Therefore, we confirmed the convergence of the result by changing the mesh settings over the air, glass substrate, and the fine structure of the antenna. For the finer mesh settings, we set the upper limit of the mesh size in the specified domain. In some cases, the software may automatically make the meshes smaller than the mesh size limit so that there is no spatially abrupt change in their size. To avoid redundancy, we call the mesh-size limit as mesh size later.

First, we simulate the propagation of the Gaussian beam in the absence of the antenna to judge the meshing of the background and glass substrate. Supplementary Figure 9a shows the incident electric field recorded by the field monitor at (0 µm, 0 µm, 0 µm) simulated with only air in the entire simulation domain. Supplementary Figure 9b shows the field simulated with air covering the entire domain of z > 75 nm and a glass substrate covering the entire domain of z < 75 nm. These figures show that the maximum amplitude of the incident field depends on the mesh settings.

Supplementary Figure 10 shows the dependence of the maximum value of incident in-plane field amplitude, corresponding to the peak field amplitude around 7 ps in Supplementary Figure 9, to the mesh sizes in the air and glass. In each graph, the amplitude is normalized to the amplitude obtained with the mesh size adopted for the later simulations. We fit the data points in each graph with a linear function. The section of this line corresponds to the error due to meshing, i.e., the ratio of the amplitude obtained with infinitely small meshes to that obtained with the adopted mesh settings. We have adopted the mesh settings that make the error reasonably small and do not make the simulation time too long. Only for the result in Supplementary Figure 10b, the section is still as large as 0.9. However, this error is resolved by setting finer mesh over the antenna structure, as discussed in the next paragraph. Based on this convergence test, the mesh sizes in the air and substrate were decided, as shown in Supplementary Table 2.

Supplementary Figure 11 shows the result of the convergence test with the antenna structure with additional mesh settings. The graphs show the normalized maximum amplitude of the enhanced field in the z-direction. The simulated enhanced field amplitude is approximately linearly dependent on the mesh size. Based on the simulation time and estimated error, i.e., section of the linear fit, we adopted the mesh size shown in Supplementary Table 2. The estimated error in the adopted mesh settings is only a few percent. In Supplementary Figure 10b, there is a large error since the mesh size was too large to separate the surface of the glass and the

monitor point. However, this was solved by setting a finer mesh around the monitor point defined by the convergence test, which will be discussed in Supplementary Figure 11.

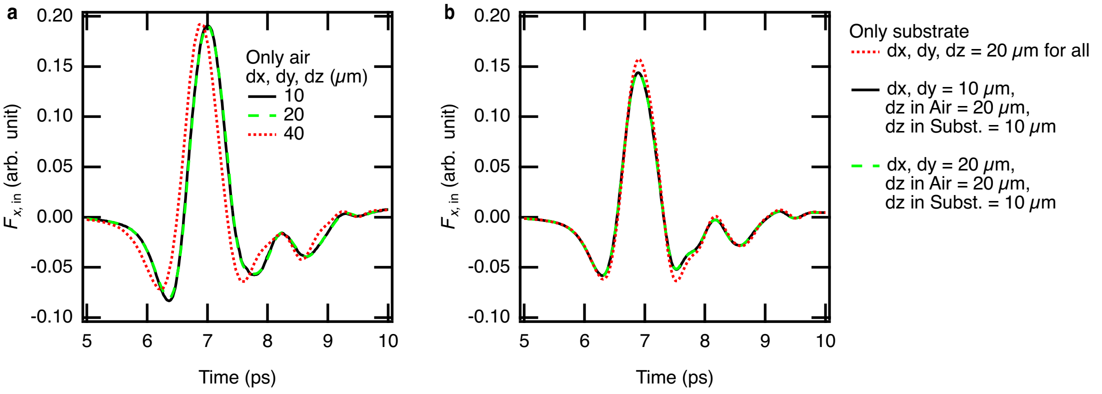

Supplementary Figure 9. Effect of varying mesh size on the incident THz waveform sampled at the origin (0 µm, 0 µm, 0 µm) without the antenna structure. **a**, Simulation with only air with varying the mesh size for the entire simulation domain as dx = dy = dz. **b**, Simulation with the air and a glass substrate with varying the mesh settings (dx and dy for all the simulation domain and dz for the glass.)

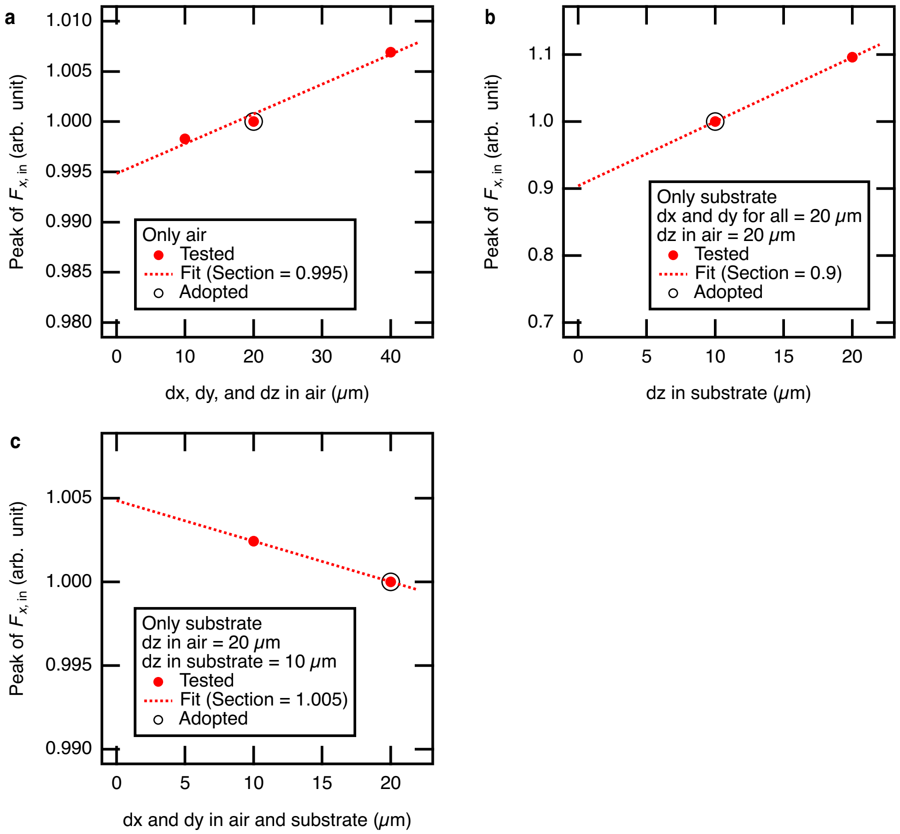

Supplementary Figure 10. Maximum incident-field amplitude to the varying mesh size in air and substrate. **a**, Test with only air with varying mesh size in the air (dx = dy = dz.) **b**, Test with air and substrate with varying

vertical mesh size (dz) in the substrate. **c**, Test with air and substrate with varying the horizontal mesh size (dx = dy) in air and substrate. In each graph, the red dots show the test results, and the open black circle shows the mesh settings adopted for the later simulations. The field amplitude in each graph is normalized to the field amplitude in the adopted settings. The red dotted line shows the linear fit of the test results. The section of the fit curve is the expected field amplitude in case infinitely small meshes are used for the simulation. The detailed mesh settings for the convergence tests are also shown in the text boxes in the graphs.

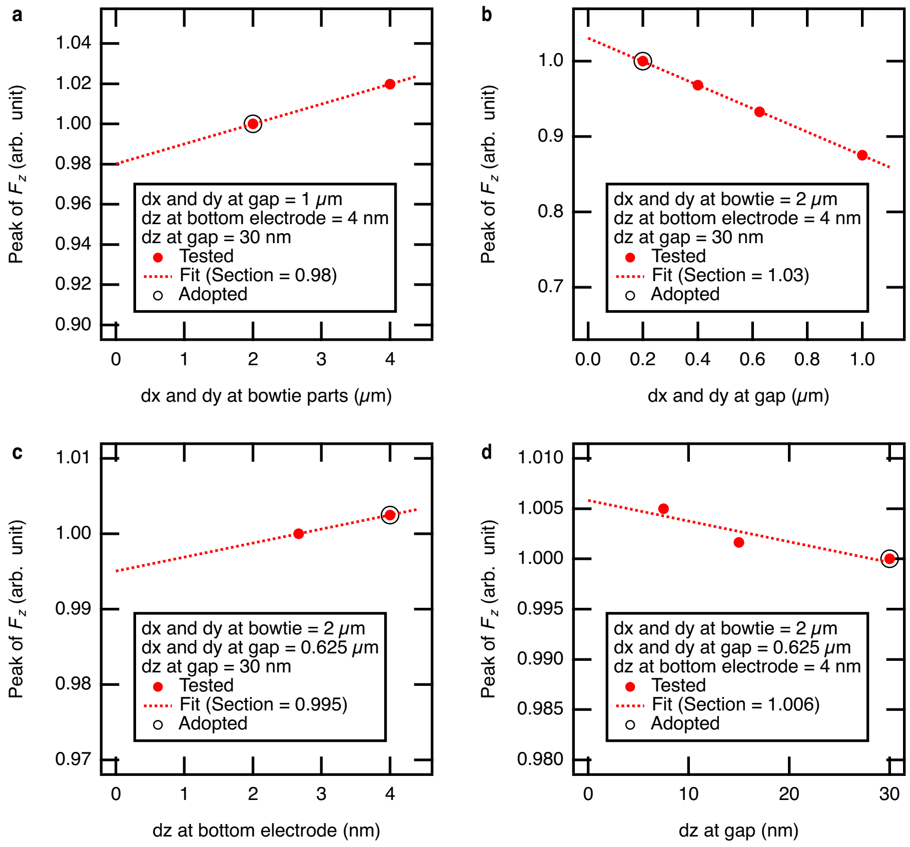

Supplementary Figure 11. Maximum incident-field amplitude to the mesh settings at the antenna structure, plotted in the same way as Supplementary Figure 9. **a**, With varying the horizontal mesh size (dx = dy) at bowtie parts of the top and bottom electrodes. **b**, With varying the horizontal mesh size (dx = dy) at the gap of the antenna structure. **c**, With varying the vertical mesh size (dz) at the bottom and top electrodes. When the vertical mesh size for the bottom electrode was 4 and 2.67 nm, the vertical mesh size for the top electrode was 12.5 and 6.25 nm, respectively. **d**, With varying the vertical mesh size (dz) at the gap.

Supplementary Table 2. Mesh size adopted based on the convergence test.

Direction	Domain	Mesh size (Upper limit in the domain)
x, y	Entire simulation domain	20 μm
z	Air 1	20 μm
z	Subst 1	10 μm
z	Air 2	Not specified
z	Subst 2	Not specified
x, y	Bowtie (bottom and top)	2 μm
x, y	Gap	0.2 μm
z	Bowtie (bottom)	4 nm
z	Bowtie (top)	12.5 nm for device with 25-nm top electrode and 25 nm for device with 50-nm top electrode
z	Gap	30 nm

1-5. FDTD simulation results: Field enhancement

Supplementary Figure 12 shows the simulated waveforms of an incident in-plane THz field $F_{x,\mathrm{in}}$ without the antenna structure and the enhanced out-of-plane plane THz field F_z at the gap of 2D-3D hybrid antenna. Supplementary Figure 12a shows the incident THz waveform in a long temporal window of 0 to 28 ps. The signal after 25 ps is the THz wave reflected at the simulation-domain boundary. Supplementary Figure 12b shows the out-of-plane THz waveform in the long temporal window. This waveform has two components: One is a fast component dominant in the time range of 5-10 ps and resembles the incident waveform. This is a resonance to the bowtie shape of the antenna. The other is a slow component dominant in the time range of 10-25 ps and does not resemble the incident waveform. This is a resonance to the large-scale structure, including the contact pad. Supplementary Figure 12c shows the incident in-plane THz field and out-of-plane THz field plotted with a time offset to adjust the peak at 0 ps. The amplitude of the out-of-plane field is 13.3 times larger than the incident in-plane field. Although the out-of-plane field has a slightly lower frequency compared to the incident field due to the antenna's resonance frequency, the overall waveform of the fast component of the out-of-plane field resembles the incident waveform.

Supplementary Figure 13 shows the spectral information of the simulated waveforms. Supplementary Figure 13a shows the intensity spectra of the in-plane and out-of-plane THz fields. Supplementary Figure 13b and c show the response function of the antenna structure as the ratio of the amplitude spectra and the difference of the phase spectra between the in-plane and out-of-plane THz fields.

Supplementary Figure 14 shows the difference of the out-of-plane THz field caused by the antenna structures with a thickness of the top electrode of 25 nm and 50 nm. The peak amplitude of the fast component at 7.5 ps has almost no difference in these devices, and it is only 0.8% higher for the device with a 50-nm-thick top electrode compared to a 25-nm-thick top electrode.

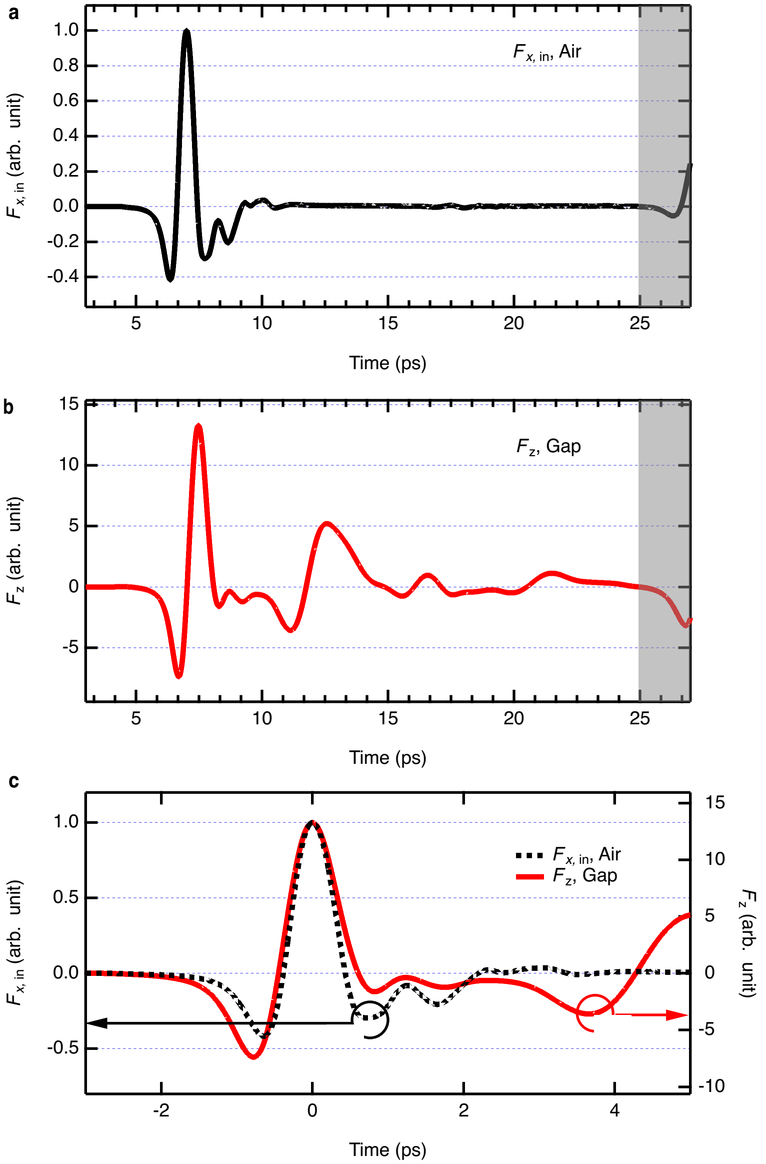

Supplementary Figure 12. Simulated waveforms of incident in-plane THz field and out-of-plane THz field caused by the antenna structure. **a**, Incident in-plane THz field without the antenna structure. **b**, Out-of-plane THz field caused by the antenna structure. The gray-colored area after 25 ps is affected by the back-reflected waves at the simulation boundaries. **b**, Incident in-plane field and out-of-plane field plotted together to show the time offset of the waveforms. **c**, Incident in-plane field and out-of-plane field plotted with a time offset adjusted to the field peak. In all the plots, the field amplitude is normalized to the peak of the incident in-plane field.

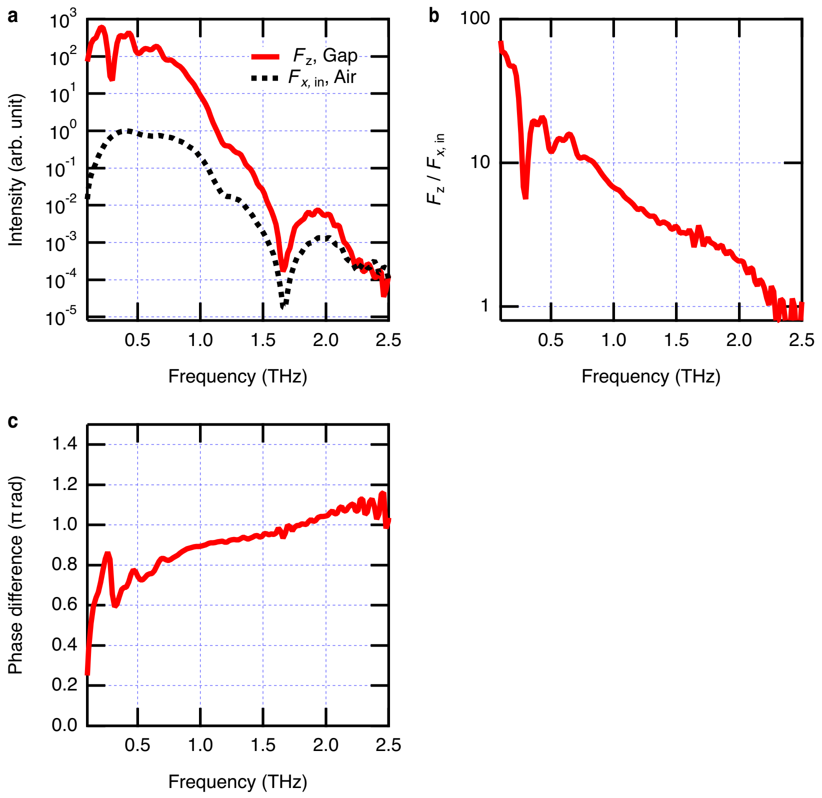

Supplementary Figure 13. Spectral information of the simulated waveforms. **a**, Intensity spectra of the incident in-plane THz field and out-of-plane THz field caused by the antenna. **b**, Raito of amplitude spectra of the incident in-plane THz field and out-of-plane THz field. **c**, Difference of phase spectra of the incident in-plane THz field and out-of-plane THz field.

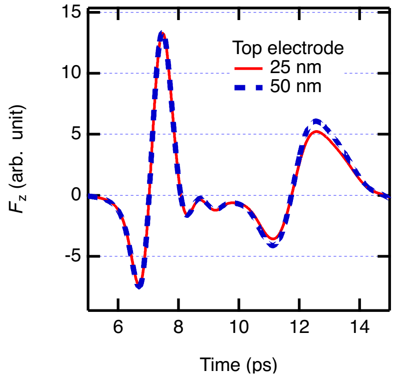

Supplementary Figure 14. Simulated out-of-plane field waveform for antenna devices with the top-electrode thickness of 25 nm and 50 nm. The scaling of the vertical axis is the same as that of Supplementary Figure 12.

1-6. FDTD simulation results: Spatial inhomogeneity

Our antenna generates the out-of-plane field in the gap with spatial inhomogeneity that is unique to this system: Due to the spatial charge distribution defined by the antenna's resonance, the out-of-plane field in the field-enhancement region has a slight inhomogeneity in the x-direction as shown in Supplementary Figure 15.

Supplementary Figure 16 shows the waveforms of the THz field probed at three different points in the antenna gap. The waveforms have a delay of 160 fs and a peak amplitude difference of 12 % for the two points located on the opposite side of the gap in the x-direction, representing the points of maximum field inhomogeneity.

In our measurement, the effect of such a spatially distributed THz field is averaged by the optical probe spread over the gap region. However, as we see in Fig. 16a and b, the difference in the waveforms is not so large, and it does not significantly affect the observed result.

In addition, in relation to the spatial inhomogeneity of the charge distribution, we have checked the amplitude of the in-plane THz field in the gap. As shown in Supplementary Figure 17, the in-plane component is smaller than the out-of-plane component by two orders of magnitude. Therefore, the effect of the inhomogeneity on the in-plane field component is also negligible. We do not see the in-plane component from the incident field in this plot. It would be because the incident field is blocked at the top electrode and it does not diffract into the gap.

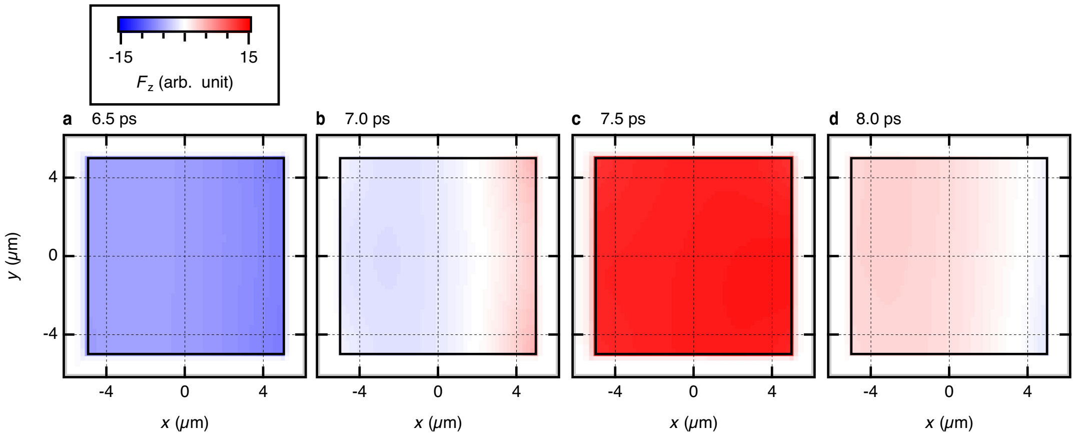

Supplementary Figure 15. Spatial distribution of simulated out-of-plane THz field around the field-enhancement region at $z = 0$ plane plotted for various timing around the main peak of the THz pulse. The time for each frame is **a**: 6.5 ps, **b:** 7.0 ps, **c:** 7.5 ps, and **d:** 8.0 ps. The field-enhancement region of 10 μm × 10 μm is shown by the solid square in each frame.

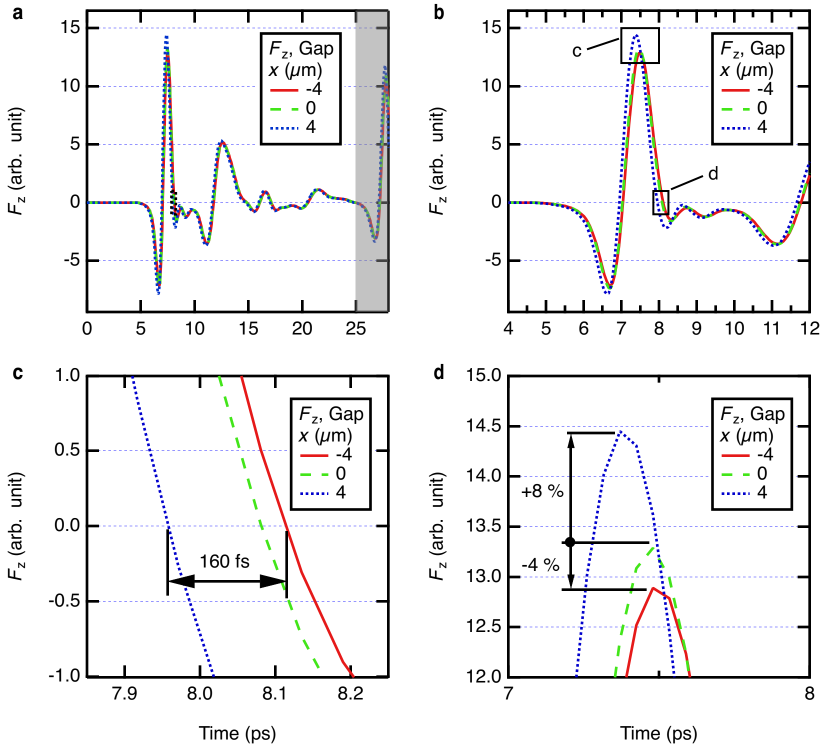

Supplementary Figure 16. Simulated out-of-plane THz field waveform in the field-enhancement region probed at different points. Coordinate of the probed points are (x,y,z) = (-4 μm, 0 μm, 0 μm), (0 μm, 0 μm, 0 μm), (4 μm, 0 μm, 0 μm), respectively. **a**, Temporal waveforms plotted over 0-28 ps. **b**, Temporal waveforms plotted over 4-12 ps. The boxes labelled as c and d indicate the region plotted in **b** and **c**. **c**, Magnified view of the waveforms around their zero-crossing points around 8 ps. **d**, Magnified view of the waveforms around their peaks around 7.5 ps.

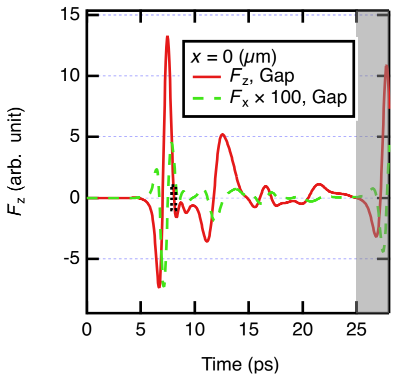

Supplementary Figure 17. Comparison of the ouf-of-plane and in-plane THz field in the antenna gap probed at (x, y, z) = (0 μm, 0 μm, 0 μm). The in-plane component is smaller than the out-of-plane component by two orders of magnitude.

Supplementary Notes 2. Simulation of optical reflectance spectra

2-1. Reflectance spectra of antenna devices with and without MoS_2

We use the electromagnetic simulation software CST Studio Suite (Frequency domain solver) to simulate the reflectance spectra of the antenna devices. Here we model the central part of the antenna shown in Supplementary Figure 18 and we apply unit cell boundary conditions in transverse directions (XZ &YZ planes) to simplify the simulation. In the simulation, we use a plane wave at normal incidence (propagating in +z-direction from Port 1) as excitation source. This may result in slightly different behaviour, because in the experiment a focused Gaussian beam is used. However, this simplified simulation is sufficient for the qualitative discussion in this section. Further, Port 1 is placed in direct contact with the glass substrate. This approach eliminates reflections at the backside of the substrate and allows the substrate thickness to be modelled as much smaller, while avoiding introducing unrelated Fabry-Perot (FP) effects. This way the propagation through the complete 1.1 mm glass substrate does not have to be simulated. The glass-air interface is highly transmissive, so neglecting the reflection at this interface will not make much difference. For the material properties in the optical frequency range for the simulation, the following literature values were used:

- Top electrode (50 nm) – Dielectric function of gold film reported by Olmon et al. [7]
- Bottom electrode (8 nm) – Dielectric function of 7.5 nm gold reported by Ze et al. [8]
- Al_2O_3 – Refractive index $n = 1.76$ (corresponding the dielectric function $\varepsilon = 3$) [9]
- Glass – Refractie index $n = 1.5$
- MoS_2 – Dielectric function of 3L MoS_2 reported by Yu et al. [10]

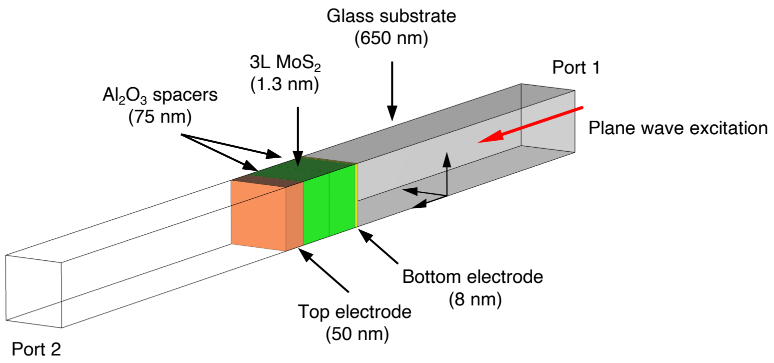

Supplementary Figure 18. Modelled center part of the antenna. The numbers indicate the layer thicknesses.

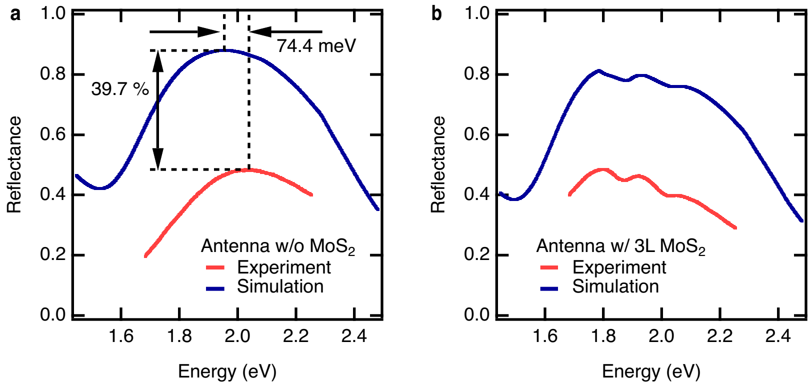

Supplementary Figure 19. a, Simulated and measured optical reflectance spectra of antenna structure without MoS_2, and **b,** that of the antenna structure with 3 layers (3L) of MoS_2. There is only 74.4 meV energy difference of simulated and measured FP peak in **a**.

Supplementary Figure 19a shows the simulated and measured reflectance spectra of the antenna without MoS_2. Both spectra show a curve originated from a FP resonance of low-Q factor caused by multi-reflection between the top and bottom electrodes. Absorption within the layers also affects this curve. There is a vertical offset of 39.7% of the simulated result compared to the experiment. This is most likely due to the focused Gaussian beam used in the experiment. In a previous study, it has been shown that the FP effect is sensitive to the beam shape [11]. In particular, the divergence of the Gaussian beam leads to decreased FP peak height. Supplementary Figure 19b shows the simulated and measured reflectance spectra with 3 layers of MoS_2. The reflectance dips caused by the MoS_2 absorption are clearly visible in both spectra. There is a vertical offset of the simulated data for the same reasons in Supplementary Figure 19a.

Supplementary Figure 20a summarises the simulated reflectance spectra of antenna without and with MoS_2 from Supplementary Figure 19a and b. The redshift of the FP resonance peak in the antenna with 3L MoS_2 is due to the increased effective cavity length. Supplementary Figure 20b shows the energy loss relative to the incident energy for the antenna simulation with the 3L MoS_2. The two MoS_2 absorption peaks coincide with the dips in the reflectance spectrum. Therefore, the reflectance dips are caused by the MoS_2 absorption peaks. The two absorption peaks correspond to A- and B- exciton absorption (ε_i peak of literature [10]).

We note that there is a small deviation of the reflectance A-dip minima (1.8765 eV) and the absorption A-peak maxima (1.8791 eV). This should be due to the FP background in the reflectance spectra. In addition, it is affected by the fact that the absorption curves (Supplementary Figure 20b) arise not only from the intrinsic material parameters but also from the spatial overlap of FP modes with the material. Further, the additional loss contributions of the gold layers can also affect the reflectance dip energy.

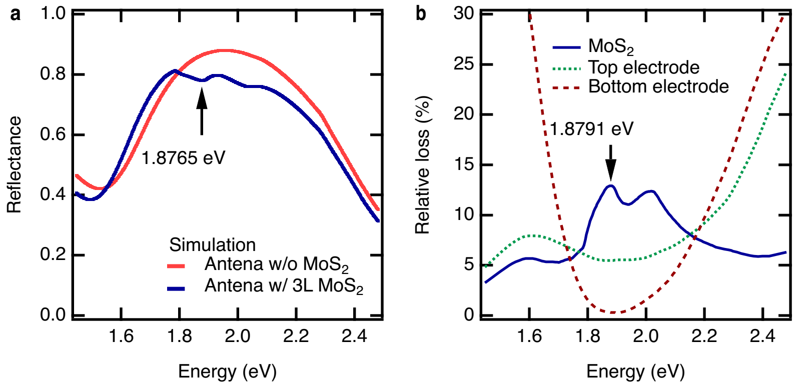

Supplementary Figure 20. a, Simulated optical reflectance spectra of antenna with and without a 3L MoS_2 (corresponding to Fig. 2a and b) and **b,** the energy loss relative to the incident energy in each of the materials of the simulation with a 3L MoS_2.

2-2. Effect of material-property change in Al_2O_3 spacer layer and gold electrodes

In this section, we show that (I) the broadband TPOP response of the antenna without MoS_2 most likely originates from the change of refractive index of Al_2O_3 spacer and (II) the shift of the reflectance dips of the antenna with MoS_2 is originated from the shift of absorption peaks in MoS_2. To address these points, we simulated two possible effects in addition to the absorption-peak shift of MoS_2, which could potentially lead to a shift of the reflectance dips when the strong THz is present in the antenna gap:

A. The change of dielectric function of the Al_2O_3 spacers due to electro-optic effect or carrier trap
B. The change of conductivity of the gold electrodes due to a modulation of charge density when the strong THz field is present in the antenna gap (Further explained in Supplementary Notes 2-3)

We numerically investigate the effects of the change of material properties of Al_2O_3 as well as the gold on the antenna reflectance spectrum in the following. In the subsequent simulation sweeps, the material parameters are swept in a range that leads to a noticeable change in the reflectance spectrum. It should be noted that the change of material parameters required for this is much larger compared to the realistic strength of the underlying effects discussed above.

B2-A. Change of dielectric function of Al_2O_3 spacers

The dielectric function of Al_2O_3 may be expressed by $\varepsilon(\omega) = \varepsilon_0\varepsilon_r + i\,\sigma/\omega$, where $\varepsilon_0, \varepsilon_r$ and σ are the vacuum permittivity, real part of the relative permittivity, and conductivity, respectively. The simulated results for different ε_r of the Al_2O_3 is shown in Supplementary Figure 21. The increase ε_r leads to a redshift of the FP resonance due to an increase of the effective cavity length. It results in the broadband change of reflectance, which is an increase in a certain energy range (1.5-2.15 eV) and a decrease in another range (> 2.15 eV) as ε_r increases, as shown in Supplementary Figure 21a. This behaviour qualitatively matches with the experimentally observed TPOP response of an antenna without MoS_2 (see Fig. 2a of the main text), which is an increase of

reflectance for the energy lower than about 1.95 THz and decrease for the energy higher than that, while the amount of the reflectance change is only on the order of $\pm$ 1%. Therefore, slight change of ε_r and the FP resonance can be the origin of the TPOP response of antenna without MoS_2.

A shift of A-dip minima of Δ_A = 10.3 meV can be observed when ε_r is increased from 2.84 to 3.16 ($\Delta\varepsilon_r$ = 0.32), as shown in Supplementary Figure 21b. However, at the same time, the FP-mode shifts as much as 84.8 meV in the lower energy side of the FP resonance. Such a large alteration of the FP curve is not observed in the experiment, in which only a local change in the reflectance dips is observed. Therefore, a change of ε_r of Al_2O_3 spacer layer is not the origin of reflectance dip shift observed in the TPOP measurements.

The simulated reflectance spectra of the antenna with increased conductivity σ of the Al_2O_3 is shown in Supplementary Figure 22a and b. The increasing absorption in the Al_2O_3 strongly alters the FP background spectrum: the reflectance decreases as the conductivity increases in the entire spectral range. This behaviour of the FP background does not match with the experimentally observed TPOP response of the antenna without MoS_2, which shows an increase and decrease in a different energy range. Therefore, a change of conductivity in Al_2O_3 spacer layer is not the origin of reflectance dip shift.

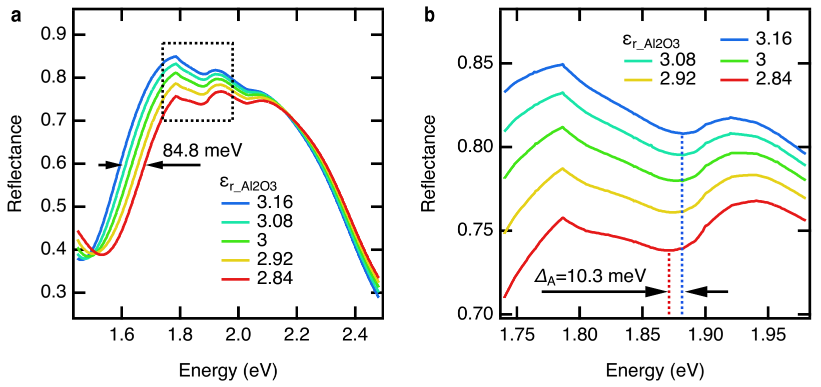

Supplementary Figure 21. a, Simulated optical reflectance of antenna with 3L MoS_2 with different real-valued permittivity of the Al_2O_3 spacers. **b,** Magnified plot of **a** corresponding to the region shown by the dotted box.

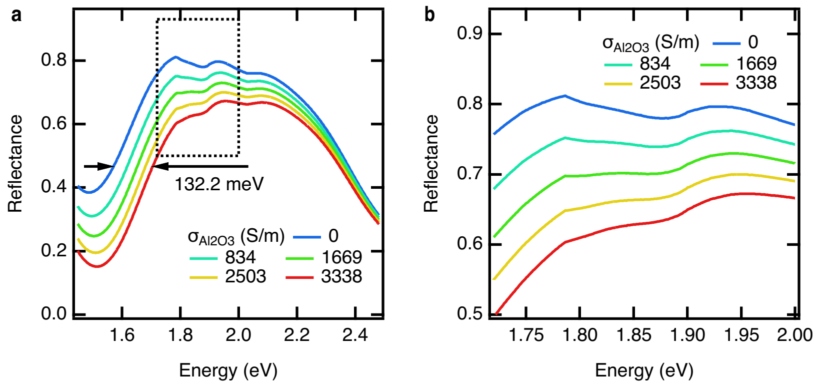

Supplementary Figure 22. a, Simulated optical reflectance of antenna with 3L MoS_2 with different conductivities of the Al_2O_3 spacers. **b,** Magnified plot of **a** corresponding to the region shown by the dotted box.

B2-B. Change of conductivity of gold electrodes

Supplementary Figure 23a and b show the simulated reflectance spectra for different dielectric functions of the top electrode layer. Here the literature dielectric function $\varepsilon_{\mathrm{Au,top}}$ of the gold is scaled by multiplying a real-valued coefficient c on both real and imaginary part. The scaled dielectric functions are shown in Supplementary Figure 23c and d.

Supplementary Figure 24a and b show the simulated reflectance spectra for the scaled dielectric functions of the bottom electrode layer. Here the literature dielectric function $\varepsilon_{\mathrm{Au,bottom}}$ of 7.5-nm thick gold is scaled by a real-valued coefficient c. The scaled dielectric functions are shown in Supplementary Figure 24c and d.

A shift of A-dip minima of Δ_{A} = 11.6 meV and 14.9 meV can be observed when the scaling of the dielectric function c increases from 0.6 to 1.4. However, a large modulation of the FP background, which was not observed experimentally, should occur at the same time. Also, the dielectric function of gold needs to be more than doubled (c = 0.6 to 1.4) to realize the simulated dip shift, which is too large and unrealistic. We estimate the change of conductivity of gold electrodes when the strong THz field is present in the antenna gap and conclude that it should be negligibly small in Supplementary Notes 2-3. Therefore, a change of the dielectric function of top and bottom electrodes is not the origin of the shift of reflectance dips in the TPOP measurements.

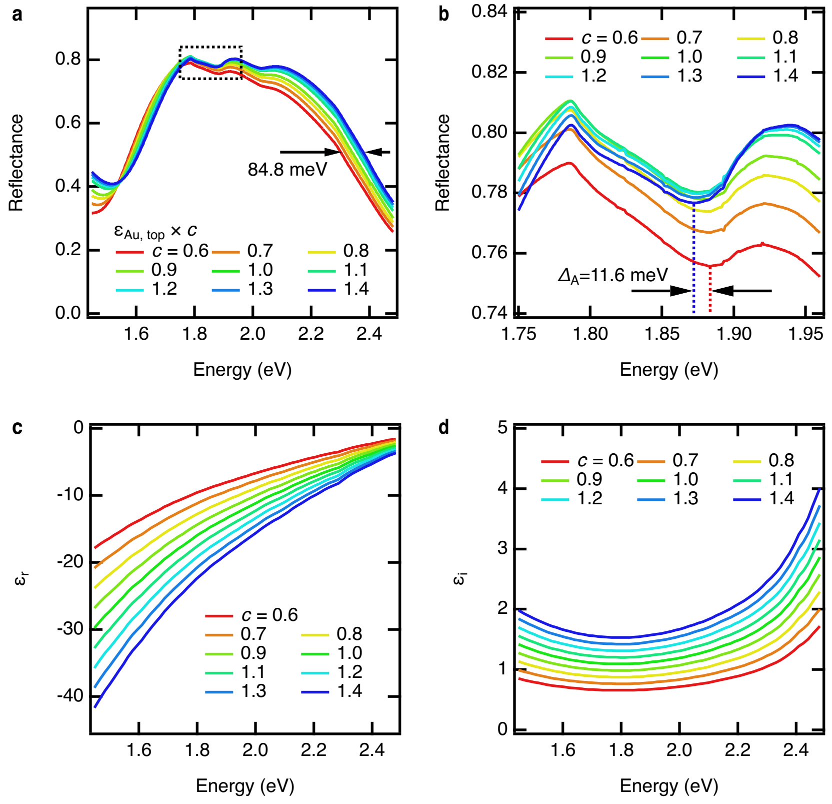

Supplementary Figure 23. a, Simulated optical reflectance spectra of antenna with 3L MoS_2 with scaled dielectric function of the top electrode layer. **b,** A magnified plot of **a** around the dips. **c,** Real part of the

corresponding scaled dielectric functions of the top electrode. **d,** Imaginary part of the corresponding scaled dielectric functions of the top electrode.

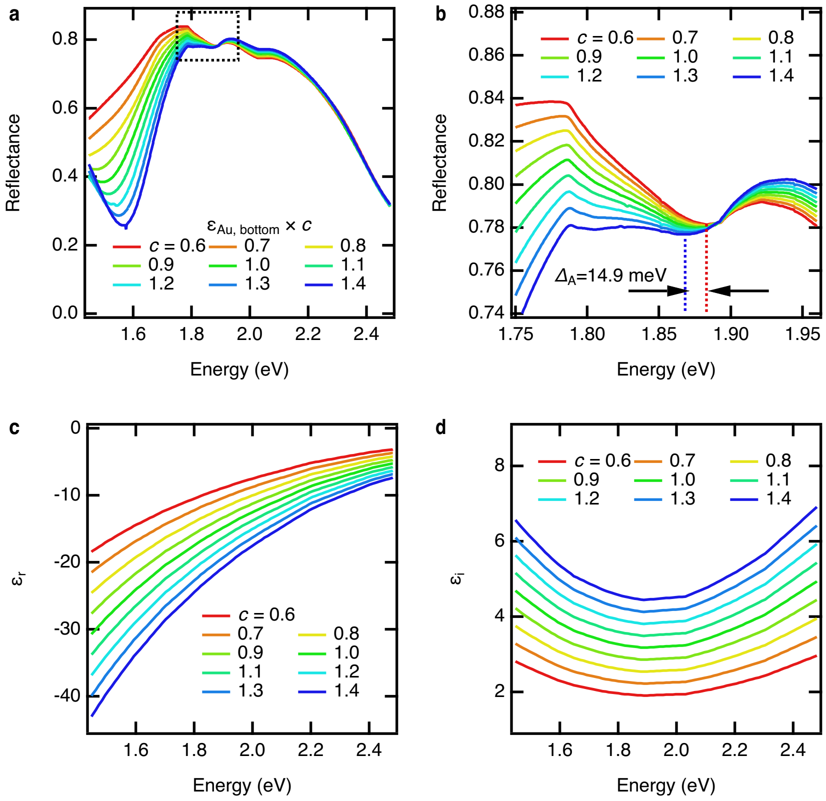

Supplementary Figure 24. a, Simulated optical reflectance spectra of antenna with 3L MoS_2 with scaled dielectric function of the bottom electrode layer. **b,** A magnified plot of **a** around the dips. **c,** Real part of the corresponding scaled dielectric functions of the bottom electrode. **d,** Imaginary part of the corresponding scaled dielectric functions of the bottom electrode.

Summary of Supplementary Notes 2-2

From the results shown in Supplementary Notes 2-2, we conclude as following:

(I) Slight change of permittivity of Al_2O_3 spacer layer causes the FP resonance shift, which is most likely the origin of the experimentally observed TPOP response of the antenna without MoS_2 shown in Fig. 2a.

(II) Shift of the reflectance dips cannot be caused by change of dielectric function of Al_2O_3 spacer nor gold electrodes without inducing unrealistically large alternation of the FP background spectrum. Therefore, the origin of the observed reflectance dip shift in TPOP experiment is the absorption peak shift of MoS_2.

2-3. Estimation of change of conductivity due to accumulation of charge on gold electrodes

The-field enhancement at our 2D-3D hybrid THz antenna occurs due to a capacitor effect: When an in-plane THz field is incident to the antenna, it causes a modulation of the charge density in the top and bottom electrodes at the centre part of antenna. These electrodes act as two capacitor plates as illustrated in Supplementary Figure 25 and causes the strong field in the field-enhancement section. In this section, we estimate the modulation of charge density.

First, we combine the two known capacitor equations

$$C = \frac{Q}{V} = \frac{Q}{E_{\mathrm{z}} d} \qquad (B1)$$

and

$$C = \frac{\varepsilon_0 \varepsilon A}{d} \qquad (B2)$$

where C is the capacitance, Q the accumulated charge, E_{z} the enhanced THz electric field, d the distance between the plates, ε the permittivity of Al_2O_3, and A the area of the capacitor (simplified to include only the centre patch of the antenna). In the first equation, the voltage V was substituted by $V = E_{\mathrm{z}} d$ under the assumption that the field in the gap is homogeneous. By combining above equations, one obtains

$$Q = \varepsilon_0 \varepsilon A E_{\mathrm{z}}. \qquad (B3)$$

Under the assumption of spatially homogenous electron density on both plates one can write as well

$$Q = tAe\Delta n, \qquad (B4)$$

where t is the electrode thickness, Δn the electron-density modulation for the capacitor effect, and e the electron charge. By combining the two previous equations, we obtain

$$\Delta n = \varepsilon_0 \varepsilon A E_{\mathrm{z}} \frac{1}{tAe} = \frac{\varepsilon_0 \varepsilon E_{\mathrm{z}}}{te}. \qquad (B5)$$

The permittivity of Al_2O_3 is $\varepsilon = 3$ and the THz field in the gap is $|E_{\mathrm{z}}| = k|E_{\mathrm{x,in}}|$, where $|E_{\mathrm{x,in}}| \approx 10^7\ \mathrm{V/m}$ is the incident THz field strength and $k = 13.3$ is the field enhancement factor, which was obtained through FDTD simulation in Supplementary Notes 1. The thickness of the bottom and top electrodes is 8 and 50 nm, respectively. Using these values, the electron-density modulation of bottom electrode $\Delta n_{\mathrm{bottom}}$ and that of top electrode Δn_{top} can be calculated as

$$\Delta n_{\mathrm{bottom}} \approx 2.8 \times 10^{24}/\mathrm{m}^3 \qquad (B6)$$

and

$$\Delta n_{\mathrm{top}} \approx 4.4 \times 10^{23}/\mathrm{m}^3. \qquad (B7)$$

These electron-density modulation potentially lead to an increase of conductivity. The increase can be estimated using the Drude conductivity model. The electron density affects the plasma frequency ω_p as

$$\omega_p = \sqrt{\frac{(n + \Delta n)e^2}{\varepsilon_0 m^*}} = \sqrt{\frac{ne^2}{\varepsilon_0 m^*} + \frac{\Delta n e^2}{\varepsilon_0 m^*}}, \qquad (B8)$$

where n is the electron density of the gold in absence of the THz field (equilibrium) and m^* is the effective electron mass. For gold the effective mass is approximately $m^* = 1.1 \times m_e$ [7], where m_e is the electron mass, and the plasma frequency of gold is in the order of $\omega_p \approx 10^{16}\ \mathrm{s}^{-1}$ [7]. This means that the first term in the square root, which gives the plasma frequency of the gold in absence of the THz (equilibrium), is of the order 10^{32}. The second term constitutes the change of the plasma frequency due to the electron-density change on the capacitor plates when the enhanced THz field is present. Using the previous values for Δn, we obtain the following values for the second term $\left(\frac{\Delta n e^2}{\varepsilon_0 m^*}\right)$ for each electrode:

$$\frac{\Delta n_{\mathrm{bottom}}\, e^2}{\varepsilon_0 m^*} \approx 8.0 \times 10^{27}\ \mathrm{s}^{-2} \tag{B9}$$

and

$$\frac{\Delta n_{\mathrm{top}}\, e^2}{\varepsilon_0 m^*} \approx 1.3 \times 10^{27}\ \mathrm{s}^{-2}. \tag{B10}$$

These terms are at least four orders smaller than the first term (10^{32}) in the plasma frequency formula.

Thus, the change of the plasma frequency ω_p due to the capacitor effect is negligible. The plasma frequency lies also far above the observed frequency range in the experiment. Thus, our measurement is not sensitive to small changes of ω_p. We conclude that the change of conductivity in the gold electrodes will be negligible in the observed TPOP response.

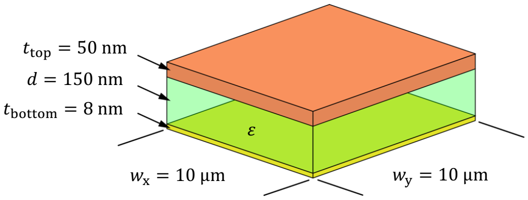

Supplementary Figure 25. Illustration of the centre part of the antenna structure without MoS_2.

2-4. Injecting carrier to 2D material via direct contact to an electrode

We could make a similar device structure without one of the top or bottom spacer layers to electrically contact the 2D material layer and an electrode for the ultrafast carrier density control of 2D material. The change of surface charge density corresponding to that of eq. (B6) and eq. (B7) is $2.2 \times 10^{12}\ \mathrm{cm}^{-2}$. With some optimization, such as reducing capacitance, it will be possible to realize a modulation of $10^{13}\ \mathrm{cm}^{-2}$, thus causing a significant modification of the properties of 2D materials [12][13]. Therefore, it will be a promising platform for ultrafast 2D material control through charge density.

Supplementary Notes 3. Chirp correction of TPOP measurement data

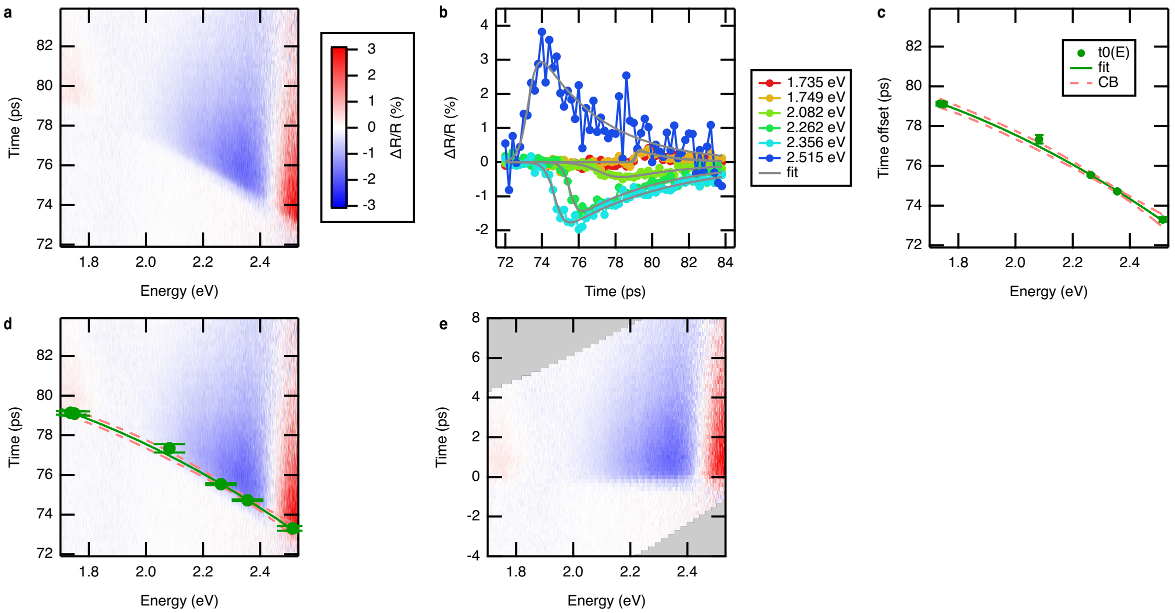

Supplementary Figure 26. Method of chirp correction. **a,** A transient reflectance-change spectrum of an antenna without MoS_2 before chirp correction. The vertical axis is the time delay defined by the optical delay stage. **b**, Transient reflectance change at several probe-photon energies. Gray lines are fitting curves. **c,** Time offset plotted against photon energy obtained via fitting of the transient reflectance shown in Supplementary Figure 26b. The error bar of the points are the standard deviation as a fitting coefficient. The solid line is the fitting curve to the time offset with a parabolic curve (time-offset curve.) Confidence band (CB) with a confidence level of 95% is also plotted to show the range where the true time-offset curve may exist, which has only about 0.2-0.3 ps width. **d**, The transient reflectance-change spectrum of an antenna without MoS_2 with the time-offset curve and its confidence band. **e**, The transient reflectance-change spectrum after chirp correction. The vertical axis is the time delay after THz pump. The data points with gray color did not exist in the original data. They are treated as the points which does not have a value.

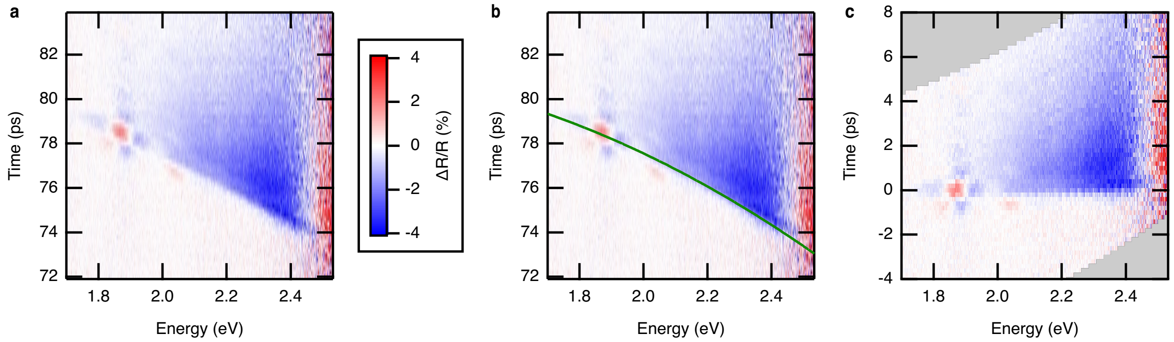

Supplementary Figure 27. Chirp correction for the TPOP data of antenna devices with MoS_2. **a,** A transient reflectance-change spectrum of an antenna without MoS_2 before chirp correction. **b**, The transient reflectance-change spectrum of an antenna without MoS_2 with time-offset curve obtained in the analysis shown in Supplementary Figure 26. **c**, The transient reflectance-change spectrum after chirp correction.

In the original data of TPOP measurement, a probe-energy-dependent delay was observed in the transient reflectance change of antenna devices without MoS_2 as shown in Supplementary Figure 26a. The THz-pump response of the antenna without MoS_2 is originated from the change of dielectric properties of Al_2O_3 spacer layer as discussed in Supplementary Notes 2. Such a response is broadband and not have a probe-energy-dependent response time. Therefore, the origin of the probe-energy-dependent delay of the signal is the chirp of the probe pulse, i.e., each energy component of the probe beam has different timing offset to the THz pump.

To obtain the reflectance change as a function of the actual time delay after THz pump independent of the probe energy, we performed a chirp correction as follows: (i) Plot the transient reflectance change ($\Delta R/R$) at several energies as a function of the time delay in the experiment t as shown in Supplementary Figure 26b. Then, fit the transient reflectance change with the following phenomenological formula:

$$\Delta R/R = A\ \exp(-\,t/T_2)/[1+\exp(-(t-t_0)/T_1)] \qquad (C1)$$

Here, A, T_1, T_2, and t_0 are amplitude, rising time, decay time, and time offset, respectively. (ii) Plot the time offset t_0 against the probe photon energy as shown in Supplementary Figure 26c. Then, fit the plot with a parabolic curve, assuming the chirp up to the second order. We call the fitting curve as time-offset curve since it gives the time offset at every probe photon energy. Supplementary Figure 26d is the plot of the transient reflectance change plotted with the time-offset curve. (iii) Shifted the time axis of transient-reflectance spectrum at every energy point by the value of the time-offset curve. The shifted transient-reflectance spectrum is shown in Supplementary Figure 26e. This results in the broadband TPOP signal without the probe-photon-energy dependent delay.

The TPOP signal of an antenna with a MoS_2 flake is also affected by the probe chirp. However, it is more complicated due to the probe-energy dependent response of MoS_2, as shown in Supplementary Figure 27a.

Therefore, for the chirp correction of the devices with MoS_2 flakes, we used the time-offset curve obtained from a measurement of an antenna without MoS_2 flake in the same experimental condition, as shown in Supplementary Figure 27b. By shifting the time axis at every photon energy using the time-offset curve, we obtained the chirp-corrected data as shown in Supplementary Figure 27c.

Supplementary Notes 4. Adjustment of time offset of EO-sampling and TPOP-measurement data

In this study, we performed the TPOP measurements with a reflection probe and the EO sampling measurements with a transmission probe, as shown in Supplementary Notes 8. Therefore, the position of the optical translation stage in these two measurements are different, and the measurement results have an unknown time offset. In this section, we describe how we adjusted the time offset when we make the plot of dip position against input THz field.

Supplementary Figure 28 shows all the Lorentzian parameter of the transient reflectance spectra obtained from a TPOP measurement (a-f) and the input THz field obtained from EO sampling (g and h). Here, the time offset of the THz waveform of Supplementary Figure 28g and h are adjusted so that the timing of peak THz field is roughly the same as that of the maximum dip shift. Furthermore, the time offset for Supplementary Figure 28g is adjusted by our fine-tuning policy described below. The offset for Supplementary Figure 28h is shifted by 200 fs compared to that of Supplementary Figure 28g for comparison.

Supplementary Figure 29 and 30 are the plot of Lorentzian parameters vs input THz field based on the time offset of Supplementary Figure 28g and Supplementary Figure 28h, respectively. As shown in Supplementary Figure 29e and f, the trace of dip position vs incident THz field is nearly single valued for the fine-tuned time delay. On the other hand, as shown in Supplementary Figure 30e and f, the trace is no more single valued, and it gets an apparent hysteresis loop when the time delay is off from the optimal one. If the dip shift is an caused by QCSE as we model, it should be instantaneous to the incident field and does not have a hysteresis. Therefore, to obtain the optimal time offset, we plotted the dip against incident field for various offset value and chose the one which gives the minimal apparent hysteresis loop.

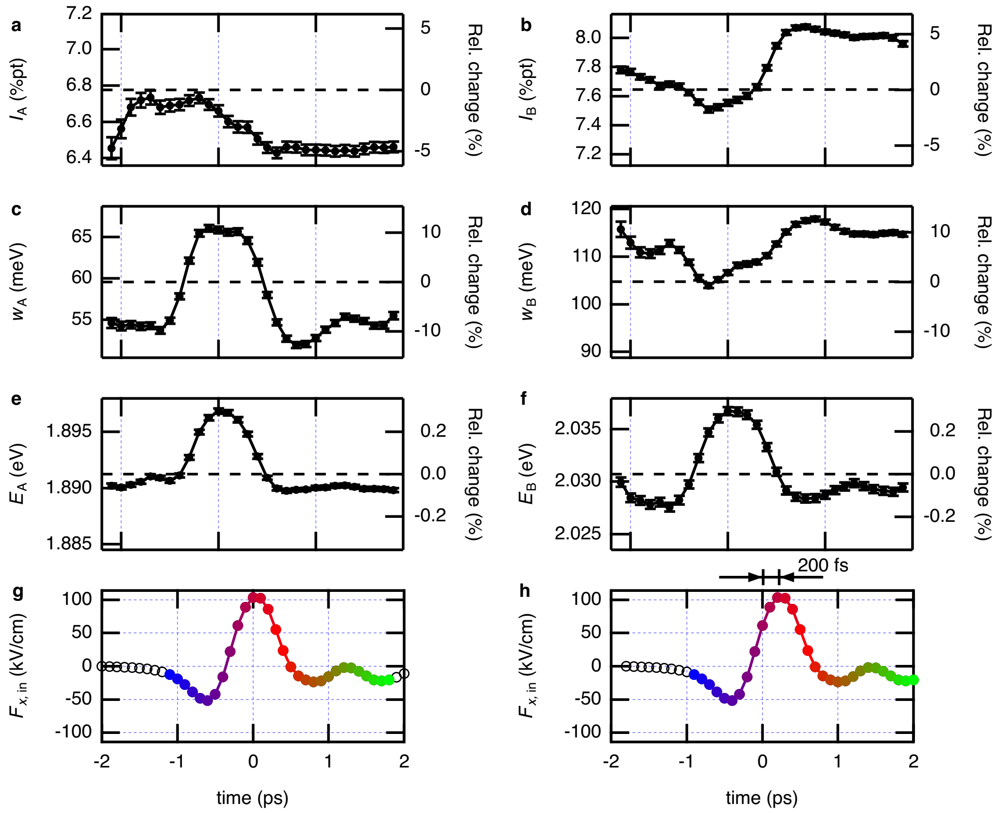

Supplementary Figure 28. All the Lorentzian-fit parameter of the transient reflectance spectra obtained from a TPOP measurement (a-f) and the input THz waveform obtained from EO sampling (g and h) as a function of delay time. **a**, Amplitude, **c**, width, and **e**, center of the A-dip. **b**, Amplitude, **d**, width, and **f**, center of the B-dip. **g**, Input THz waveform with an optimal time offset. **h**, Input THz waveform which is shifted by 200 fs from that of **g**.

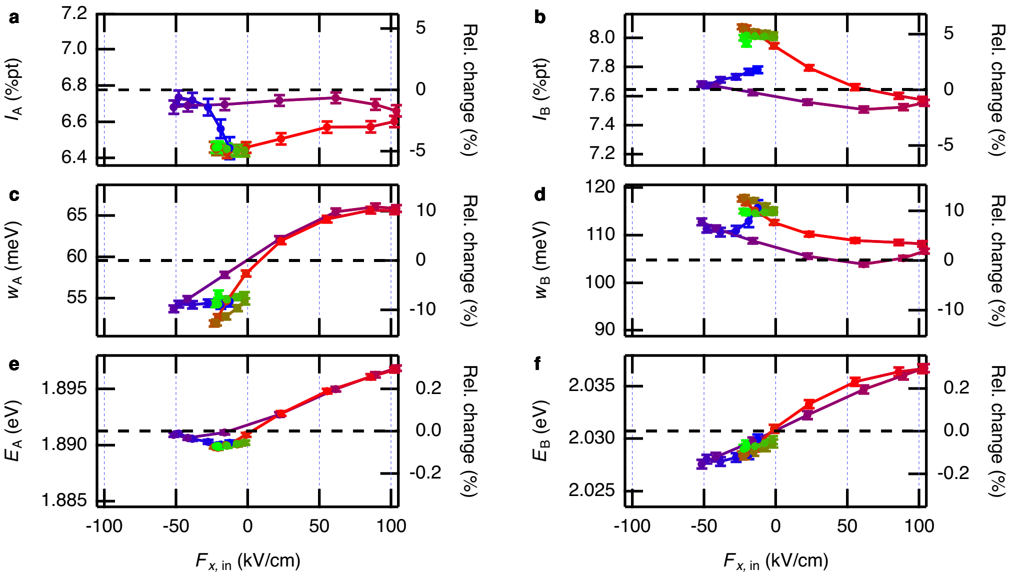

Supplementary Figure 29. Lorentzian parameter plotted against the incident THz field with the optimal time offset of Supplementary Figure 28g. **a**, Amplitude, **c**, width, and **e**, center of the A-dip. **b**, Amplitude, **d**, width, and **f**, center of the B-dip. Right axes are the relative change to the values without THz pump.

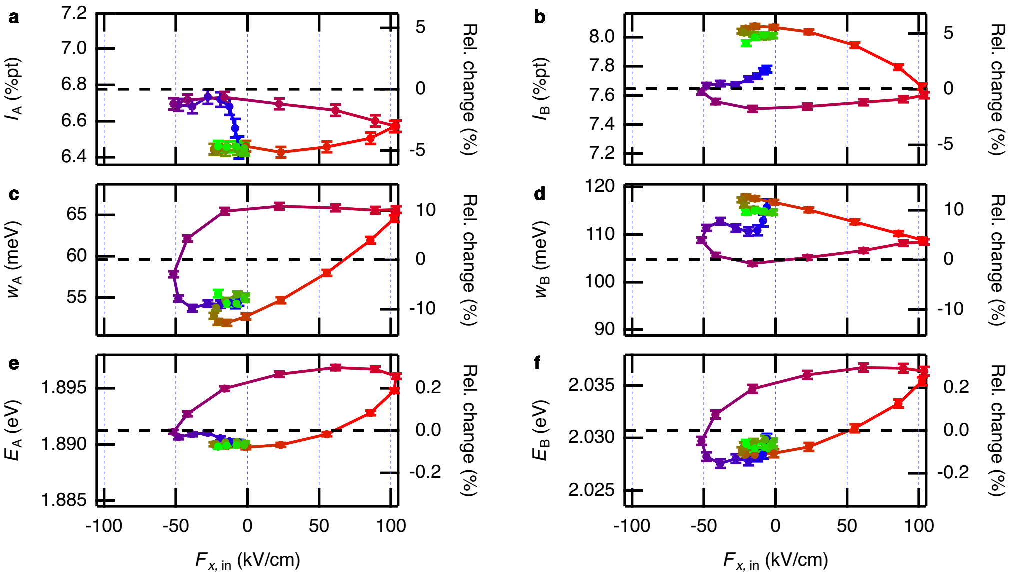

Supplementary Figure 30. Lorentzian parameter plotted against the incident THz field with the time offset of Supplementary Figure 28h, which is 200 fs off from the optimal value. **a**, Amplitude, **c**, width, and **e**, center of the A-dip. **b**, Amplitude, **d**, width, and **f**, center of the B-dip. Right axes are the relative change to the values without THz pump.

Supplementary Notes 5. DC biasing

5-1. Leak current and dielectric-breakdown threshold measurement of devices without MoS_2 flakes

We measured the leak current and the dielectric-breakdown threshold under a DC bias voltage for four 2D-3D hybrid THz antenna devices without MoS_2 flakes. We have connected a source meter (SMU 2450, Keithley) to the two contact pads of the device with a bonding wire and measured the current-voltage curve with two-terminal method. The tested samples were fabricated in the condition of α-batch.

The antennae without MoS_2 from α-batch had large sample-to-sample variation of the leak current and the breakdown threshold. The leak current was typically up to 8 to 40 μA at 110 to 150 V, and they went through the breakdown under the larger voltage. Supplementary Figure 31 shows the results for the antenna without MoS_2, fabrication number #1 from α-batch. Fabrication number is an original index given to all the samples during fabrication, which is noted here for traceability of fabrication and experimental records. This sample had a leak current up to 8 μA at 145 V and went through the dielectric breakdown. After the breakdown, the antenna, connection lines between antenna to contact pads, a part of contact pads, and bond contact were burned out. Supplementary Figure 32 shows the results for the antenna without MoS_2, fabrication number #2 from α-batch. It had a leak current up to 8 μA at 150 V and went through the dielectric breakdown. Before the breakdown, two discontinuity of the current-voltage curve was observed. These discontinuities most likely correspond to burn-down of a short-cut path of current, such as dusts connecting the two contact pads. Supplementary Figure 33 shows the results for the antenna without MoS_2, fabrication number #3 from α-batch. It had a leak current of only less than 500 pA and did not go through the breakdown of the device. This might be due to very high quality of the device occasionally achieved. Supplementary Figure 34 shows the results for the antenna without MoS_2 #4 from α-batch. It had a leak current up to 40 μA at 110 V and went through a breakdown of the bond contact.

Supplementary Figure 35 shows the results for an antenna without MoS_2, fabrication number #17 from β-batch, repeatedly measured for three times. The voltage was continuously swept in the order of 0V, +20V, -20V, and 0V as indicated by the arrows in the figure. This sample from β-batch had a leak current of only about 200 pA at 20V. Also, the reproducible I-V property shows the high stability of the spacer dielectric. Each current-voltage curve shows hysteresis loop, which may be originated by charge transfer from the electrodes to the trap states in the oxide layer.

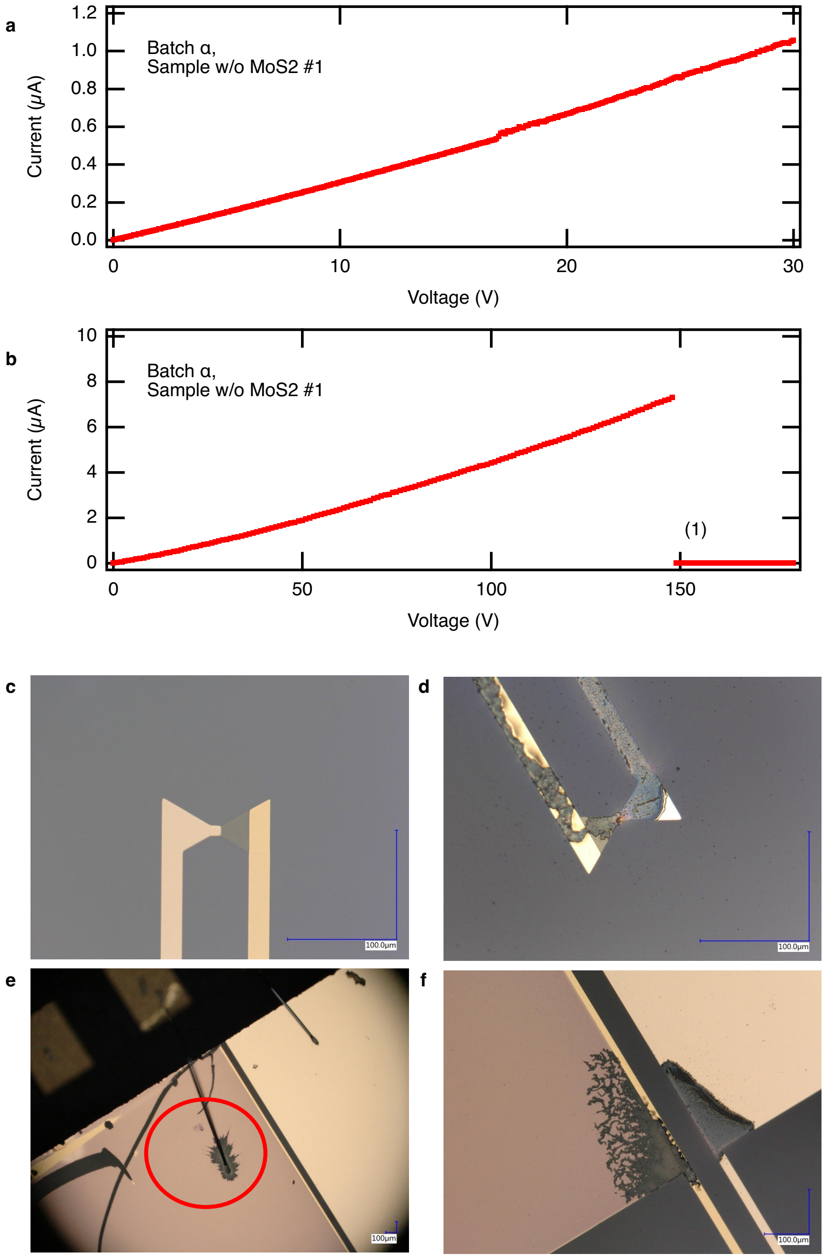

Supplementary Figure 31. Result of the leak current measurement and breakdown test for antenna without MoS_2, fabrication number #1 from α-batch. **a**, Current-voltage curve up to 30 V. **b**, Current-voltage curve up to 180 V. The point (1) corresponds to the breakdown of the device. **c**, Antenna as prepared. **d**, Antenna after the test. **e**, Bond contact after the test. **f**, Contact pad after the test.

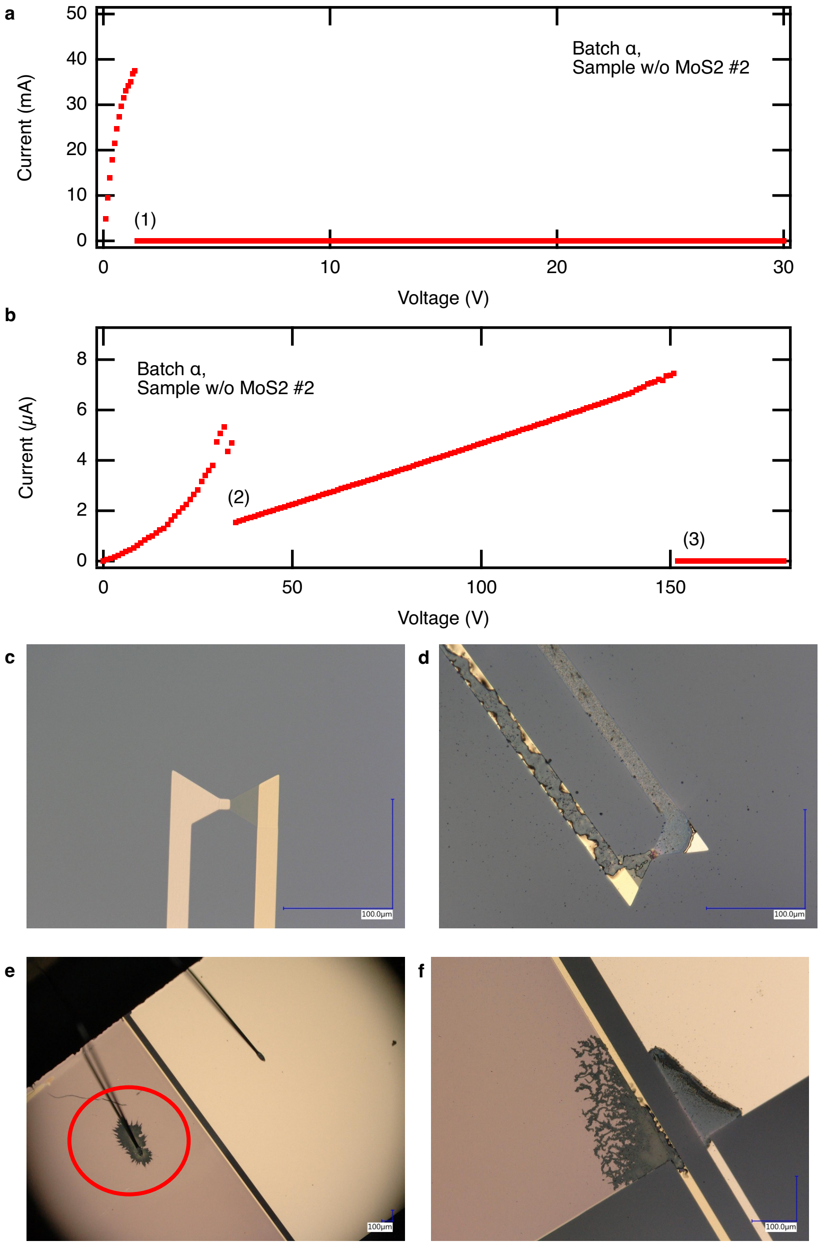

Supplementary Figure 32. Result of the leak current measurement and breakdown test for antenna without MoS_2, fabrication number #2 from α-batch. **a**, Current-voltage curve up to 30 V. **b**, Current-voltage curve up to 180 V. The points (1) and (2) probably correspond to burn-out of short-cut path for the current, such as a dust bridging the two contact pads. The point (3) corresponds to the dielectric breakdown of the device. **c**, Antenna as prepared. **d**, Antenna after the test. **e**, Bond contact after the test. **f**, Contact pad after the test.

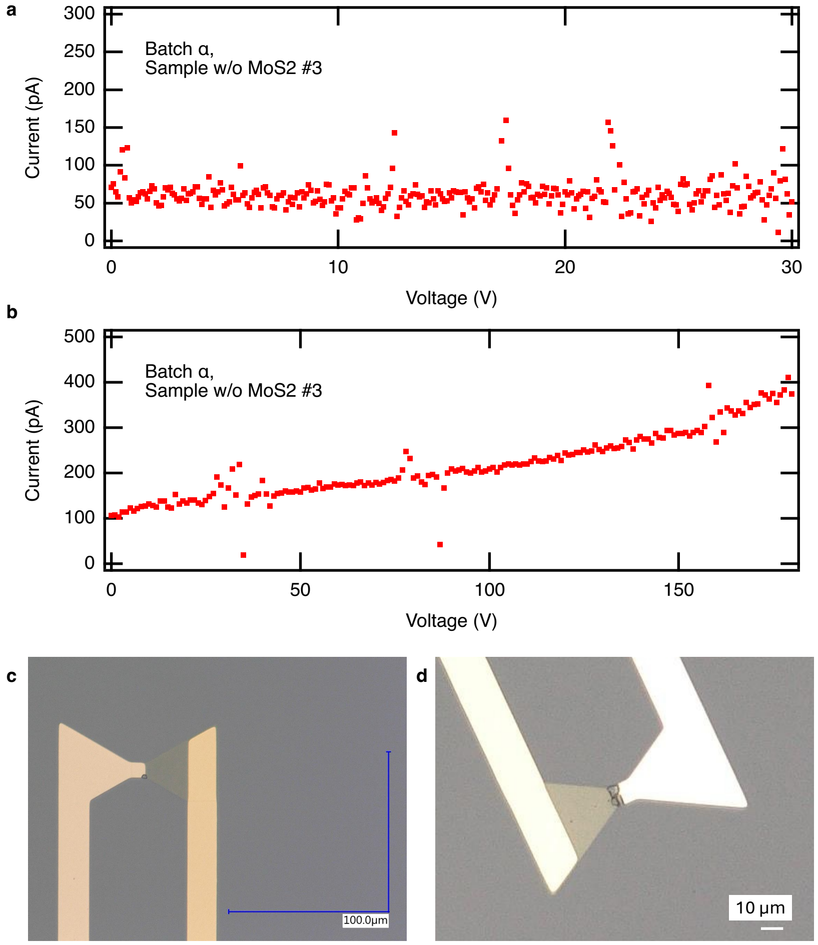

Supplementary Figure 33. Result of the leak current measurement and breakdown test for antenna without MoS_2, fabrication number #3 from α-batch. **a**, Current-voltage curve from the first measurement 1. **b**, Current-voltage curve from the second measurement up to 180 V. **c**, Antenna as prepared. **d**, Antenna after the test.

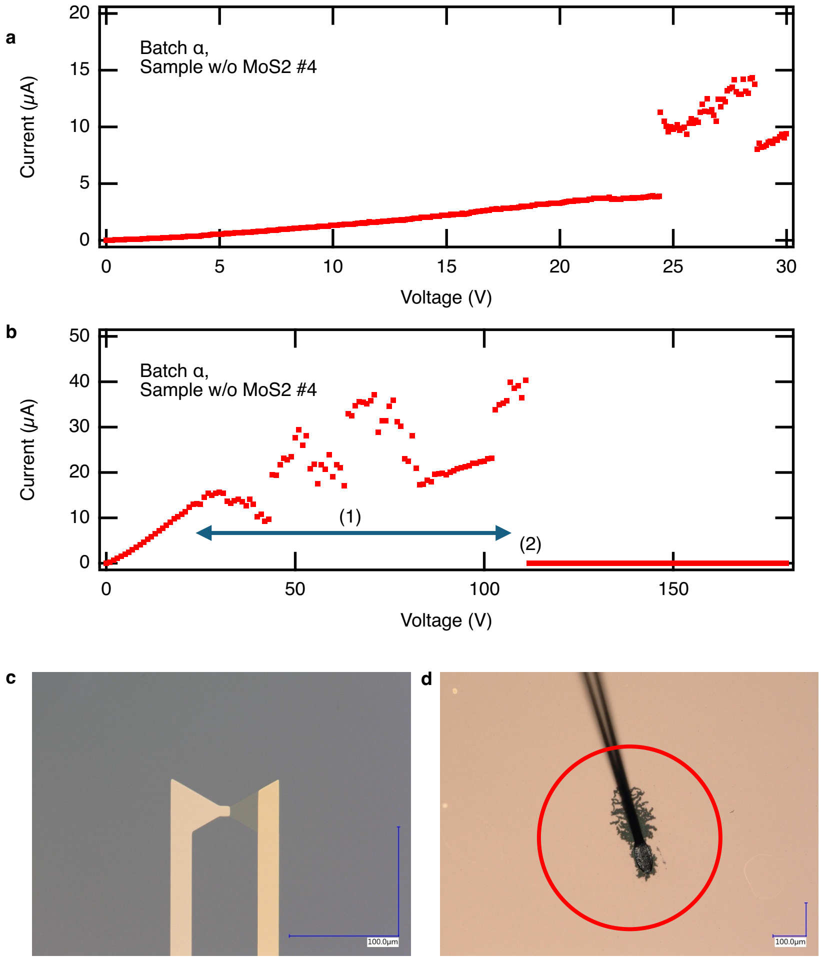

Supplementary Figure 34. Result of the leak current measurement and breakdown test for antenna without MoS_2, fabrication number #4 from α-batch. **a**, Current-voltage curve up to 30 V. **b**, Current-voltage curve up to 180 V. The regime (1) and point (2) probably correspond to destabilization and breakdown of the bond contact, respectively. **c**, Antenna as prepared. No change of antenna was observed after the point (2). **d**, Bond contact after the test.

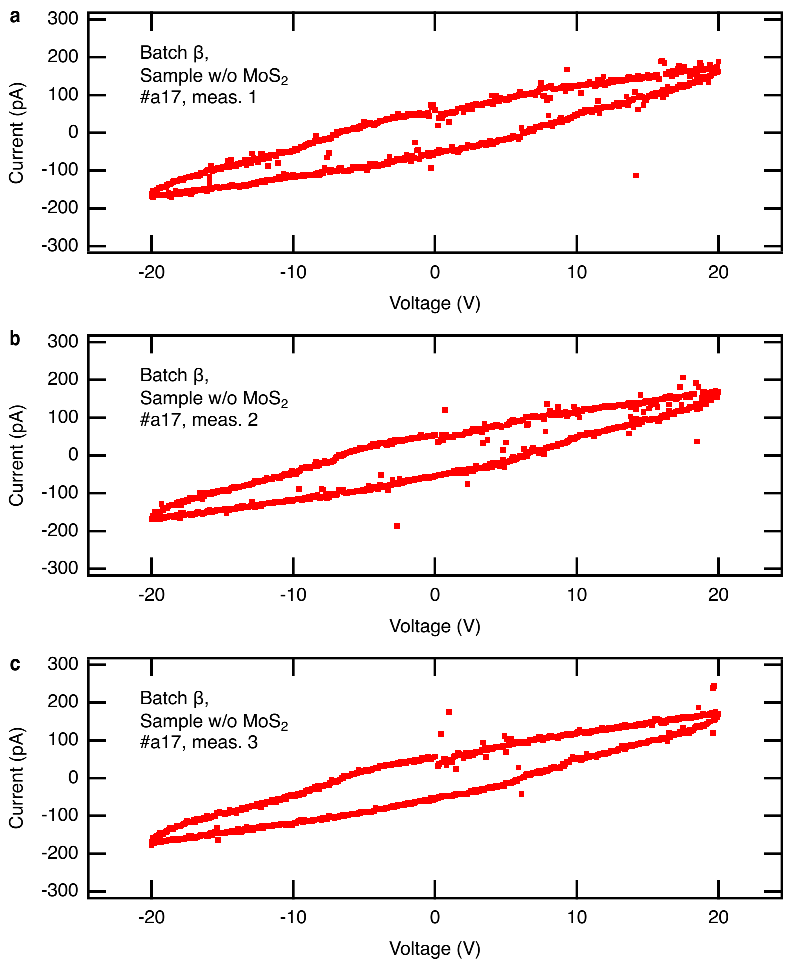

Supplementary Figure 35. Result of the leak current measurement for antenna without MoS_2, fabrication numbers #a17 from β-batch repeatedly. **a**, First measurement, **b**, Second measurement, **c**, Third measurement.

5-2. Unintentional shortcuts and dielectric breakdown of the devices under DC bias

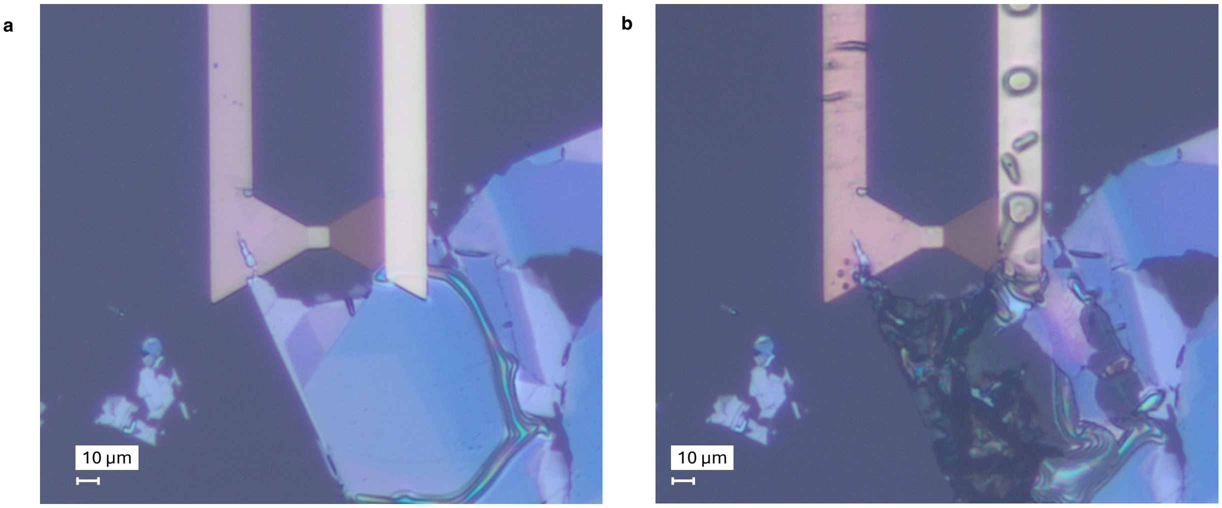

Supplementary Figure 36. Microscope image of an antenna device with MoS_2 flake before and after DC-biasing test. **a**, Before the test. A few-layer MoS_2 flake is located at the antenna gap even it is not clearly seen. In addition, thicker MoS_2 flake is located around the antenna structure. **b**, After the test. A leak current pathway from top electrode to bottom electrode through the thick MoS_2 flake is burned, and the line is deformed due to Joule heating.

Our devices had contact pads and lines connected to the top and bottom electrodes. We have applied DC-bias voltage on the devices to measure the DC-bias induced dip shift in reflectance spectra for calibration of the THz field amplitude. However, it did not work due to unexpected leak-current paths.

Supplementary Figure 36 shows an example of a device before and after such a measurement with DC biasing. In this device, not only the few-layer MoS_2 flake at the gap but also thicker MoS_2 flake was placed around the antenna structure due to the mechanical exfoliation and transfer process. After the DC biasing experiment, a change of appearance in the thick MoS_2 flake and the connection line was observed. This indicates that a leak current from the top to bottom electrodes through thick MoS_2 flew, and the dielectric breakdown took place along this leak-current path. Since most of our devices had such a leak pathway, we could not measure an effect of the DC-bias voltage. Removal of such unwanted leak and breakdown pathways will enable the MV/cm - scale DC biasing in the future studies.

Supplementary Notes 6. All the TPOP measurement data and analysis result

In Supplementary Data 1, the results of all the TPOP measurements and their analysis are presented in several types of figures. In this section, we give the caption of these figures using an example of measurement (1) for device 1 shown in Supplementary Figures 37-42. The device name, measurement number, and antenna angle are given in the folder name.

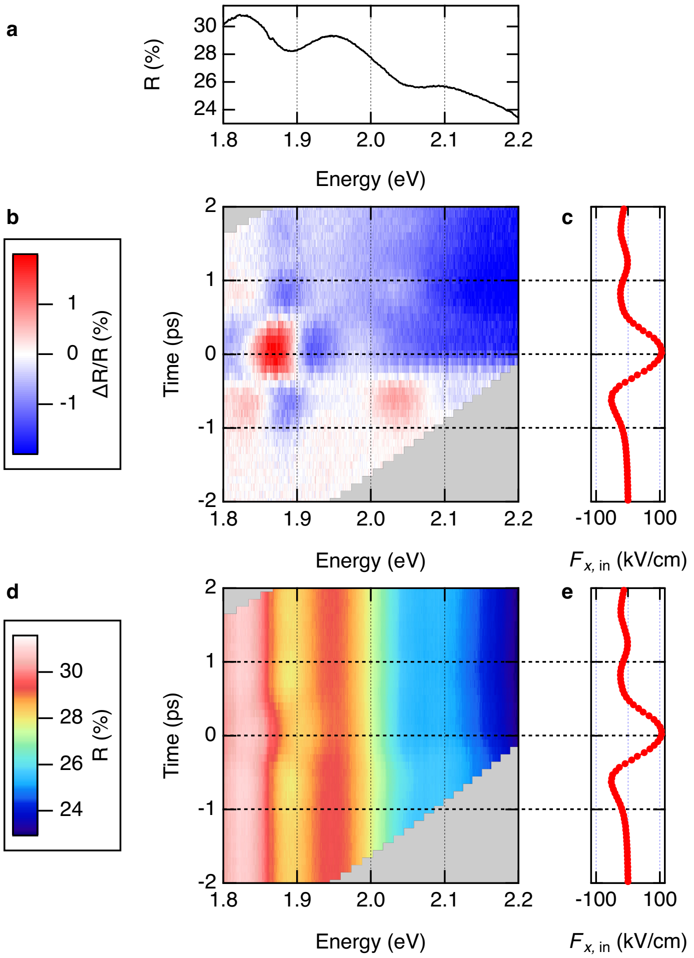

Supplementary Figure 37. An example of a figure named "XXX_summary_measTPOPdata," that shows the measured TPOP response for each antenna. XXX is an identifier for each experiment. **a**, Measured absoltue reflectance spectrum. **b**, Transient reflectance change observed in the TPOP measurement. **c**, Incident THz waveform for the TPOP measurement measured with EO sampling. **d**, Transient reflectance obtained from the reflectance spectrum and transient reflectance change shown in **a** and **b**. **e**, Incident THz waveform same as **c**.

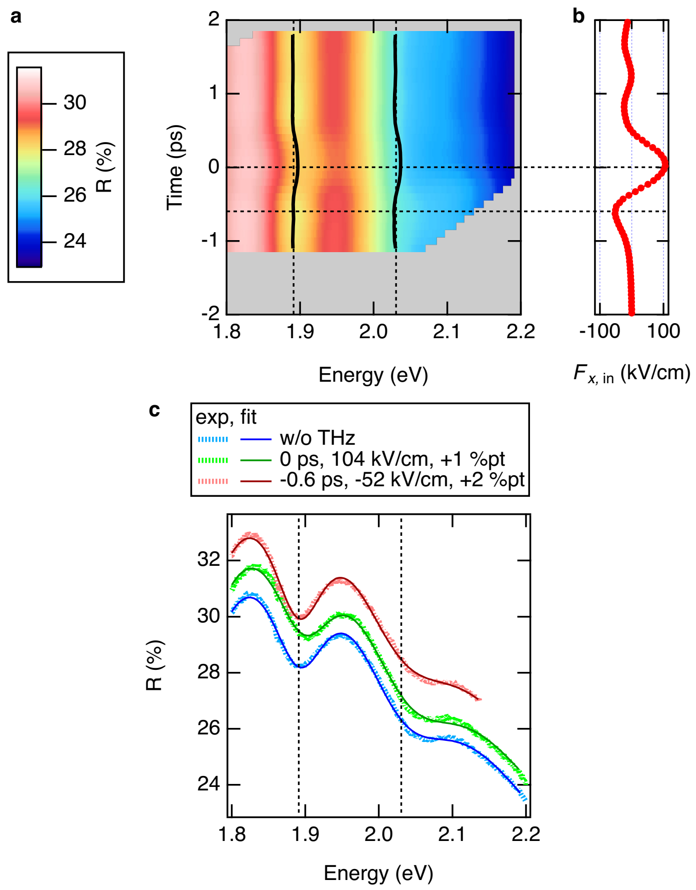

Supplementary Figure 38. An example of a figure named "XXX_REtFit_and_EOS," that shows the fitting curves of the reflectance spectra. XXX is an identifier for each experiment. **a**, Transient reflectance spectra obtained via fitting. Two dotted vertical lines show the original position of A- and B-dip without the THz field. **b**, THz waveform incident to the antenna (same as Supplementary Figure 37c.) **c**, Reflectance spectra without THz pump (blue), under THz pump of 104 kV/cm at 0 ps time delay (light green, vertically offset by +1%pt for clarity), and under THz pump of -52 kV/cm at -0.6 ps time delay (dark red, vertically offset by +2%pt for clarity). Traces of dotted lines are experimental data, and traces of solid lines are fitting. The vertical dotted line shows the position of A and B dip without the THz field.

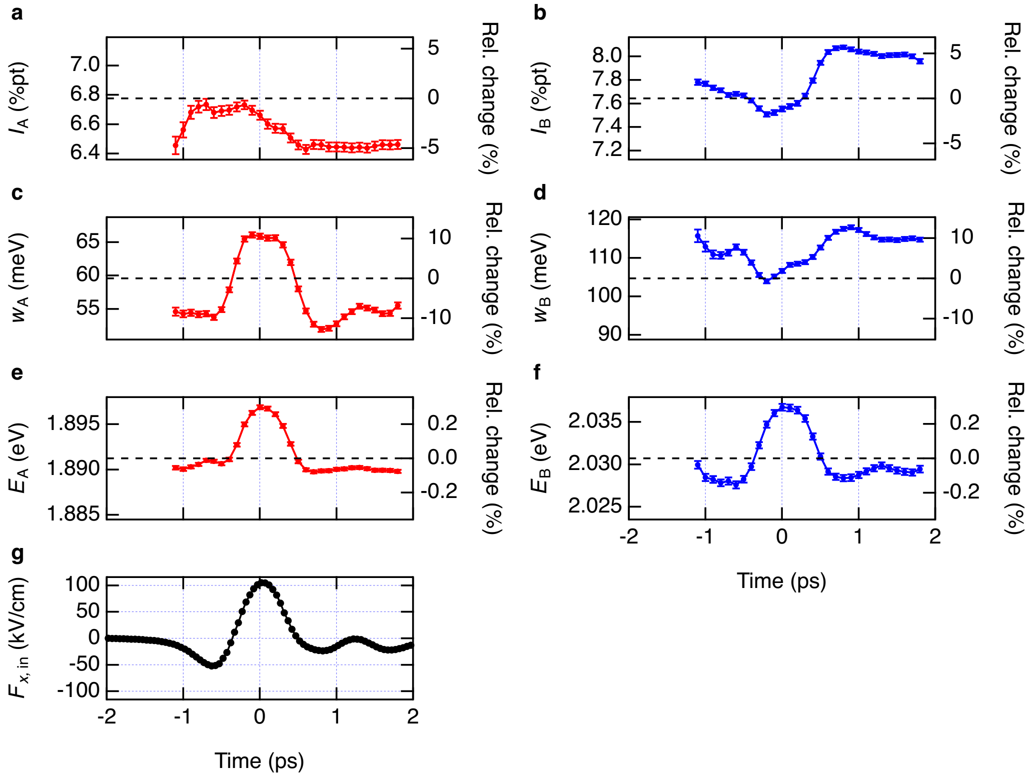

Supplementary Figure 39. An example of a figure named "XXX_REtFit_ParamVsTime," that shows the temporal change of all the fitting parameters. XXX is an identifier for each experiment. **a-b**, Ampitude of A-dip (**a**) and B-dip (**b**.) **c-d**, Width of A-dip (**a**) and B-dip (**b**.) **e-f,** Position of A-dip (**e**) and B-dip (**f**.) **g**, THz waveform incident to the antenna measured with EO sampling, under which the response of **a-f** was observed. Each horizontal dashed line in **a-f** show the original value of the parameter. The right axis in **a-f** is the relative change to the original value. Error bars in **a-f** show standard deviation as fitting parameters.

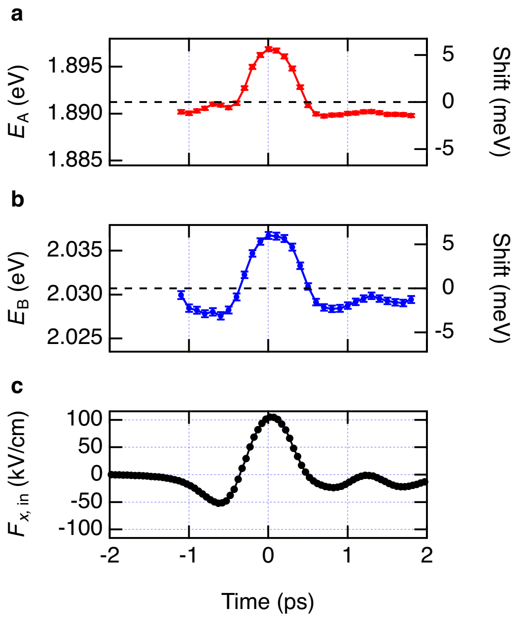

Supplementary Figure 40. An example of a figure named "XXX_REtFit_ParamEshiftVsTime," that shows the temporal change of the peak position with the energy shift shown on the right axis. XXX is an identifier for each experiment. **a-b**, The time dependence of the position of A-dip (**a**) and B-dip (**b**). Horizontal dashed lines show original dip positions without the THz field. **c**, THz waveform incident to the antenna measured with EO sampling, under which the response of **a** and **b** was observed. Horizontal dashed lines show original dip positions. The right axis in **a** and **b** is the relative change to the original value. Error bars in **a** and **b** show standard deviation as fitting parameters.

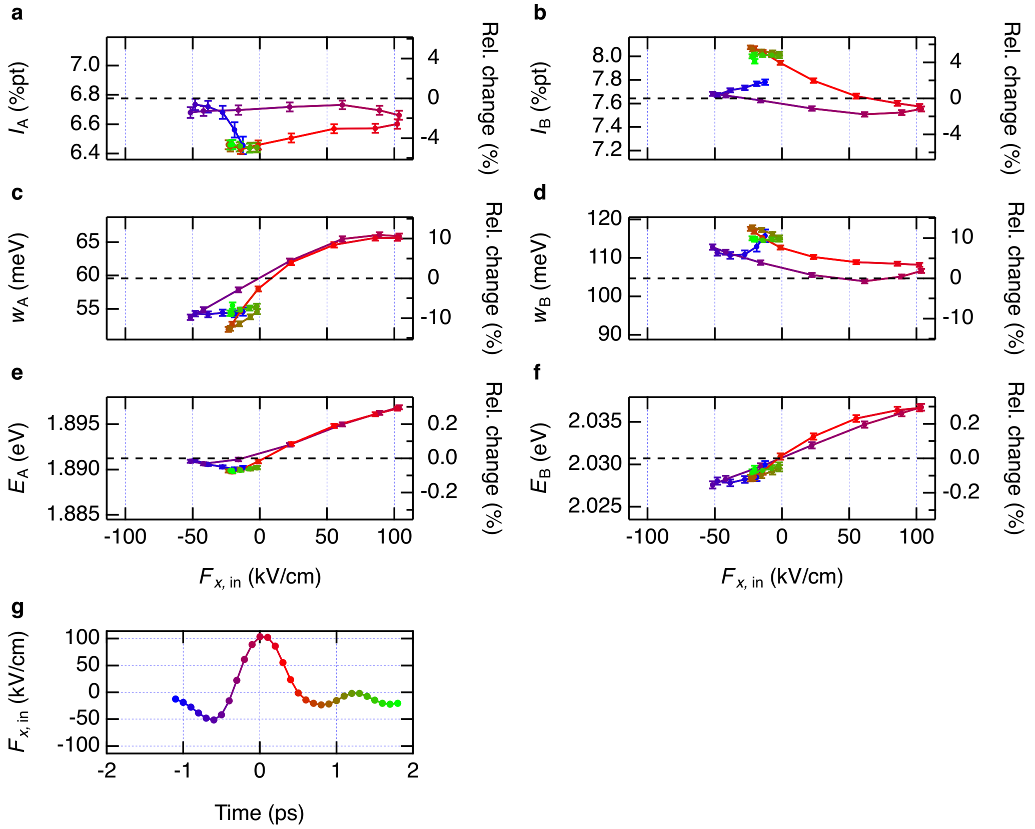

Supplementary Figure 41. An example of a figure named "XXX_REtFit_ParamVsETHz," that shows the incident-field dependence of all the fitting parameters. XXX is an identifier for each experiment. **a-b**, Amplitude of A-dip (**a**) and B-dip (**b**.) **c-d**, Width of A-dip (**a**) and B-dip (**b**.) **e-f,** Position of A-dip (**e**) and B-dip (**f**.) **g**, THz waveform incident to the antenna under which the response of **a-f** was observed. Each point represents the THz feild for the timing at the timing of each TPOP data point in **a-f**, obtained by linearly interpolating the measured data shown in Supplementary Figure 39g. Each horizontal dashed line in **a-f** show the original value of the parameter. The right axis in **a-f** is the relative change to the original value. Error bars in **a-f** show standard deviation as fitting parameters.

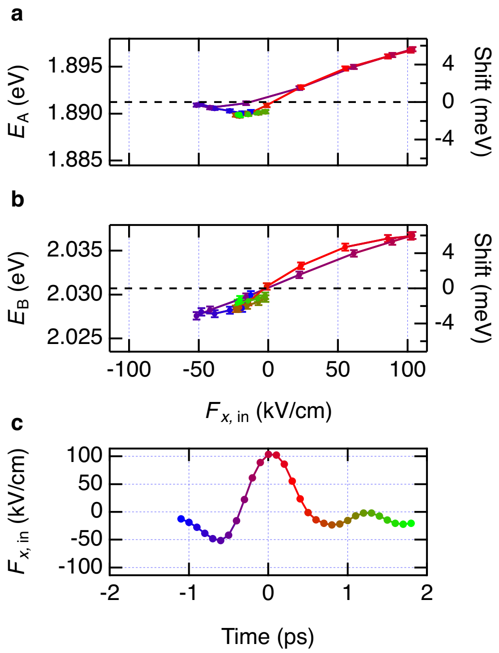

Supplementary Figure 42. An example of a figure named "XXX_REtFit_ParamEshiftVsETHz," that shows the incident-field dependence of the peak position with the energy shift shown on the right axis. XXX is an identifier for each experiment. **a-b**, The time dependence of the position of A-dip (**a**) and B-dip (**b**). Horizontal dashed lines show original dip positions without the THz field. **c**, THz waveform incident to the antenna measured with EO sampling, under which the response of **a** and **b** was observed. Each point represents the THz feild for the timing at the timing of each TPOP data point in **a-f**, obtained by linearly interpolating the measured data shown in Supplementary Figure 39g. Horizontal dashed lines in **a** and **b** show original dip positions. The right axis in **a** and **b** is the relative change to the original value. Error bars in **a** and **b** show standard deviation as fitting parameters.

Supplementary Notes 7. Photoluminescence and reflectance measurement data

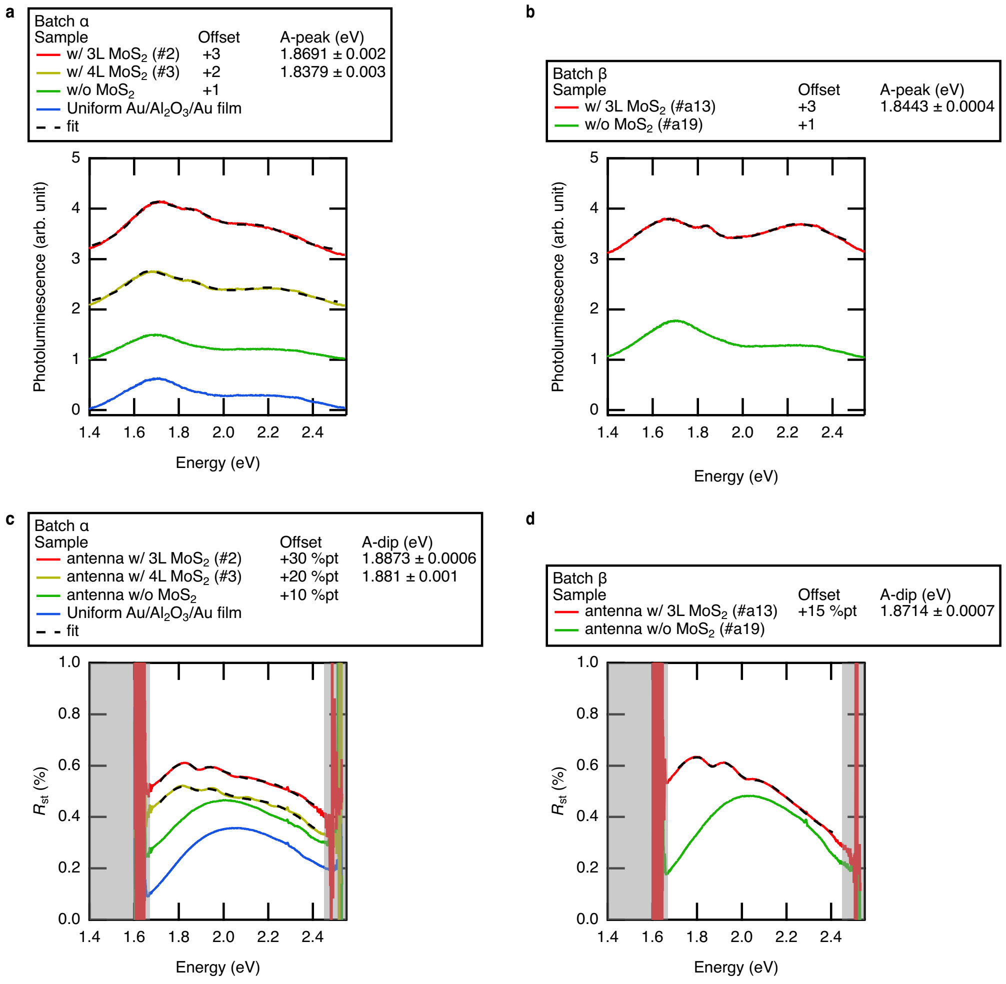

Supplementary Figure 43. PL and reflectance spectra of the devices at the field-enhancement section. **a**, PL spectra of the devices from α-batch. **b**, PL spectra of the devices from β-batch. **c**, Reflectance spectra of the devices from α-batch. **d**, Reflectance spectra of the devices from β-batch. Traces in each graph are offset by the value indicated in the legend. The black dashed lines on the traces are fitting curves. Position of the A-peak and A-dip is indicated for the fitting result of each trace is also shown in the legend.

Supplementary Table 3. Position of A-peak in PL spectrum and A-dip in reflectance spectrum for each device. Δ is the difference of the energy of A-dip to A-peak.

Batch	Fabrication number	Layer num.	Name in the main text	A-peak, PL (eV)	A-dip, Refl. (eV)	Δ (meV)
α	#2	3	Device I	1.8691 $\pm$ 0.002	1.8873 $\pm$ 0.0006	18.2
α	#3	4	Device III	1.8379 $\pm$ 0.003	1.881 $\pm$ 0.001	43.1
β	#13	3	Device II	1.8443 $\pm$ 0.0004	1.8714 $\pm$ 0.0007	27.1

Supplementary Figure 43 shows the photoluminescence (PL) and reflectance spectra of devices fabricated in this study at the field-enhancement section. We measured not only antennae with MoS_2 flakes but also an antenna without MoS_2 and an unstructured uniform film with the same layer structure to the antenna without MoS_2 of α-batch (8-nm Au/75-nm ALD-Al_2O_3/75-nm ALD-Al_2O_3/3-nm Cr/ 25-nm Au).

The PL spectra of antennae without MoS_2 and the uniform film has two broadband peaks, which is likely originated from photoluminescence of gold and an optical interference in the layer structure. In addition to these two peaks, each PL spectrum of antenna with MoS_2 has one more peak at 1.838 - 1.868 eV, which can be assigned as A-exciton luminescence [14]. The peak positions were derived by fitting to the PL spectra with three Lorentzian peaks.

The reflectance spectra of the antennae without MoS_2 and the uniform film have smooth curve originating from the optical interference in the layered structure, and that of antennae with MoS_2 have two additional dips corresponding to the A- and B-exciton absorption as discussed in Supplementary Notes 2. The dip positions were determined via fitting with two-Lorentzian-dip model shown in Fitting of Transient Reflectance Spectra in Method.

The PL peak in each device was red shifted to the reflectance dip, i.e., absorption peak, by 18 to 35 meV, as shown in Supplementary Table 3. This is consistent to the Stokes shift in MoS_2, which can be on the order of several tens of meV and dependent on the doping level [13]. The positions of the A-peaks, position of A-dips, and Stokes shift were different sample to sample. This should be originated from the different number of layer [14], dielectric disorder [15], and so on.

Supplementary Notes 8. Optical measurement detail

8-1. THz pump-optical probe reflection microspectroscopy

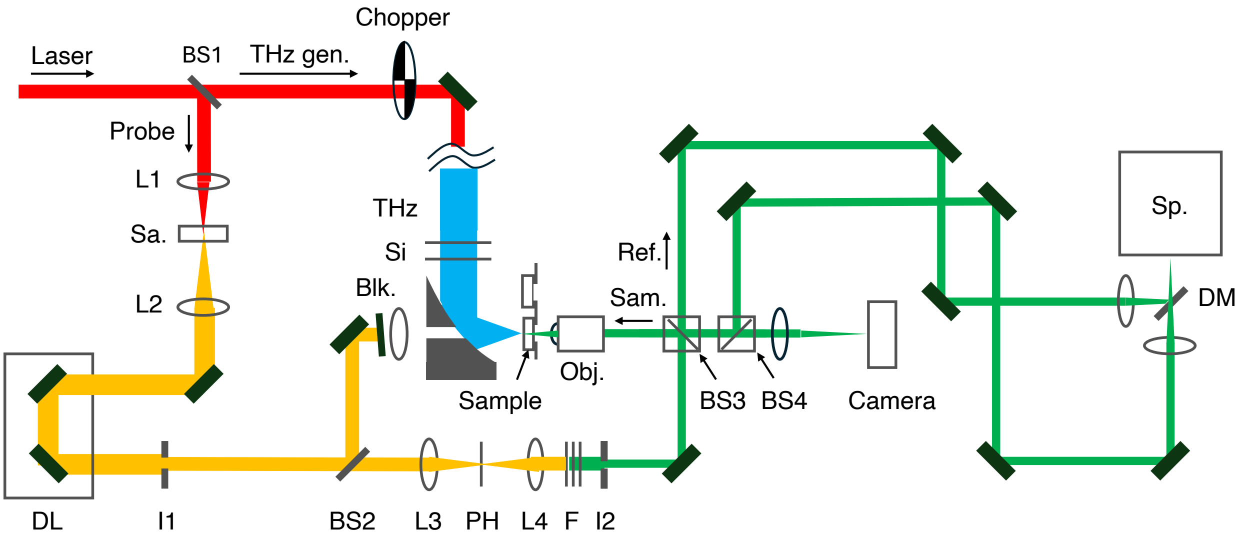

Supplementary Figure 44. Schematic optical setup for THz pump-optical probe reflection microspectroscopy. The input laser (Solstice from Spectraphysics with center wavelength of 800 nm, bandwidth of 50 nm (FWHM), repetition rate of 1 kHz, pulse energy of 6 mJ) is split into THz-generation (THz gen.) path to probe-beam path (Probe) by a beam splitter (BS) 1. In the THz-generation path, the laser was converted to an intense THz pulse via tilted-pulse-front scheme using an $LiNbO_3$ crystal. The THz pulse was attenuated via silicon (Si) wafers depending on experimental conditions and focused onto a sample, i.e., a 2D-3D THz antenna device on a grass substrate. Multiple samples and EO crystal are mounted on a single sample holder on a three-axis stage to make the condition of TPOP and EO sampling measurements the same as possible. A chopper in the THz-generation path chops the THz pulse with 50 Hz. The probe beam is loosely focused on a 4-mm thick sapphire to generate a white light. The white-light probe beam passes a mechanical delay line (DL) to control the time delay between the THz pump pulse and optical probe pulse. The probe beam is split via BS2. The reflected beam at BS2 is blocked in this measurement, and the transmitted beam is used as a probe. Optical filters are used to make the spectrum of the probe beam flat in the measured spectral range and to attenuate the energy of the probe beam to 900 fJ/pulse. Iris H1, focusing onto a pinhole of 20-μm diameter (PH), and iris H2 is used for beam shaping of the probe beam. Then, the probe beam is split via BS3. The transmitted beam is a reference beam (Ref.) which enters the spectrometer (Sp.) after being reflected via D-shaped mirror (DM). The reflected beam at BS3 is a sample beam (Sam.) which is focused on the sample, the 2D-3D hybrid THz antenna device on the backside (left side in the figure) of the glass substrate, with a 10x objective lens (Obj.). The reflected sample beam enters BS4. The sample beam transmitted BS4 is focused onto a camera to monitor the probe beam spot on the sample. The sample beam reflected at BS4 enters the spectrometer after passing through the DM. The spectra of Ref. and Sam. beams are measured simultaneously at the spectrometer. The spectra with THz and without THz are measured to obtain the transient differential reflectance.

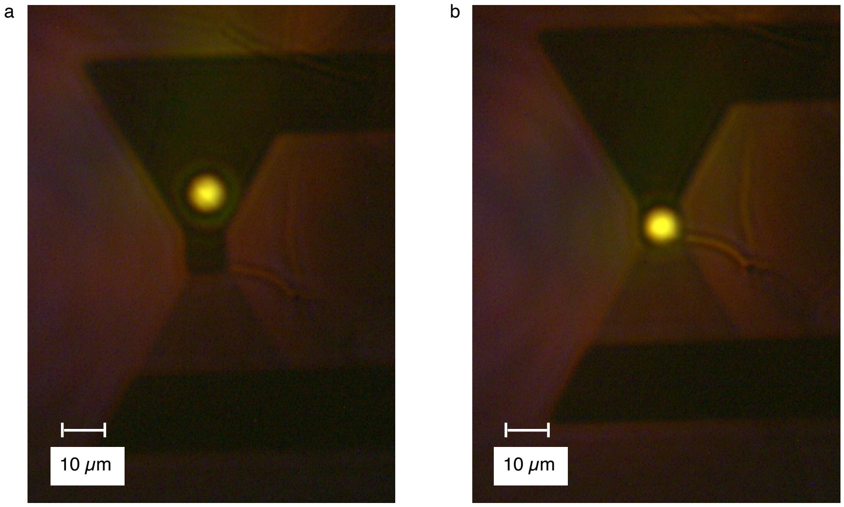

Supplementary Figure 45. Microscopic image of the probe beam and a device in TPOP measurement taken via Camera in Supplementary Figure 44. The white light reflected at BS2 in Supplementary Figure 44 was unblocked and used to illuminate the whole device in this image. **a**, Image with the probe beam focused on the field-enhancement section. **b**, Image with the probe beam out of the field-enhancement section. The focused probe beam has a shape of Airy disk. By comparing the size of the Airy disk to that of the field-enhancement section of 10 μm × 10 μm, the radius of the dark ring of the Airy disk is estimated to be 5 μm.

Supplementary Figure 44 shows the schematic setup of the TPOP measurement, and Supplementary Figure 45 shows the microscopic image of the probe beam and a device in the measurement.

The peak fluence of the probe beam at the sample was 4 μJ/cm^2. In a previous study of optical pump-probe spectroscopy of few-layer MoS_2 with a white-light pump covering the range of 1.68-2.21 eV, it has shown to be a one-photon absorption regime up to the pump energy of 900 μJ/cm^2 [16]. Therefore, our probe fluence was well in the linear regime.

In TPOP measurement, the probe spot was intentionally made to be comparable to the field-enhancement section to make the power of probe beam as high as possible without increasing the fluence. The higher probe power is necessary to perform the pump-probe time scan in a reasonable measurement time. The small clipping of the probe beam at the edge of field-enhancement section is negligible for this measurement, since the effect of clipping is cancelled for the transient differential reflectance $\Delta R/R$, which is a ratio of the reflectance change to the absolute reflectance.

8-2. Reflection microspectroscopy for static absolute reflectance measurement

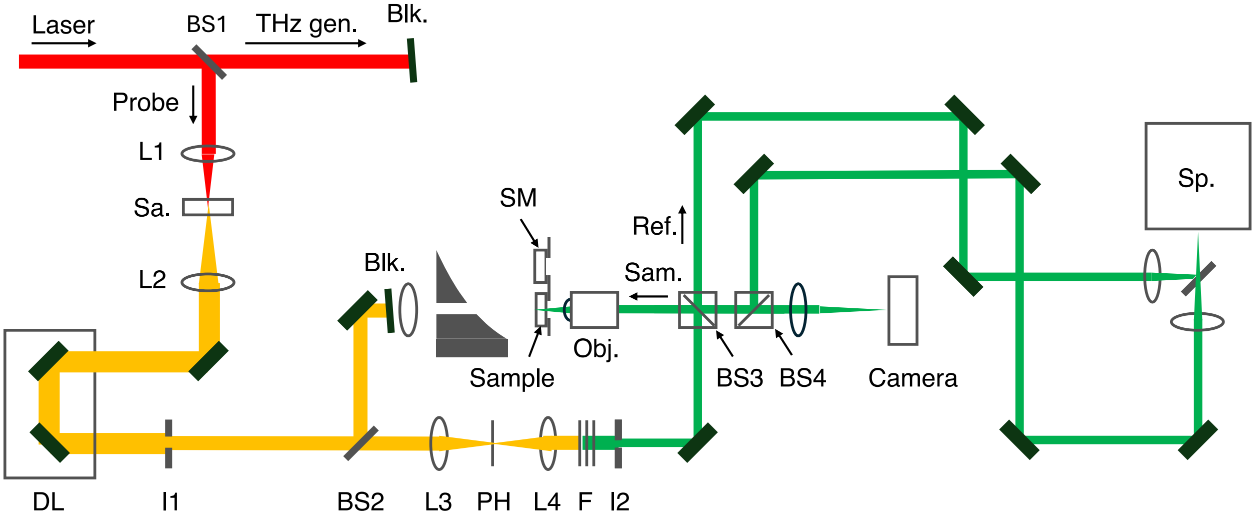

Supplementary Figure 46. Schematic optical setup for reflection microspectroscopy. The same setup as TPOP measurement is used with the THz-generation path blocked. The antenna sample and a reference silver mirror (SM) with known reflectance spectrum are mounted on the same sample holder on a three-axis stage. The measurement result of SM is used to calibrate the transfer function of the optical system and to obtain the reflectance spectrum of the sample. In this measurement, a 20x objective lens was used to make the probe beam spot small enough for the measurement of static absolute reflectance.

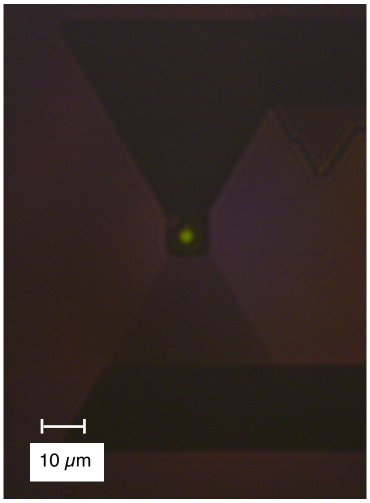

Supplementary Figure 47. Microscopic image of the probe beam and a device in TPOP measurement taken via Camera in Supplementary Figure 46. The white light reflected at BS2 in Supplementary Figure 46 was unblocked and used to illuminate the whole device in this image. The probe beam is focused on the field-enhancement section of the device. By comparing the size of the Airy disk of the probe beam to that of the field-enhancement section of 10 μm × 10 μm, the radius of the dark ring of the Airy disk is estimated to be 2 μm.

Supplementary Figure 46 shows the schematic setup of the reflection microscopy to measure the static absolute reflectance of the device, and Supplementary Figure 47 shows the microscopic image of the probe beam and a device in the measurement. The estimated peak fluence of the probe beam at the sample is 3 $\mu J/cm^2$, which is much smaller than the peak fluence needed to cause nonlinear processes. The probe spot was made smaller than the field-enhancement section to eliminate the effect of the beam clipping for the absolute reflectance measurement.

8-3. EO sampling

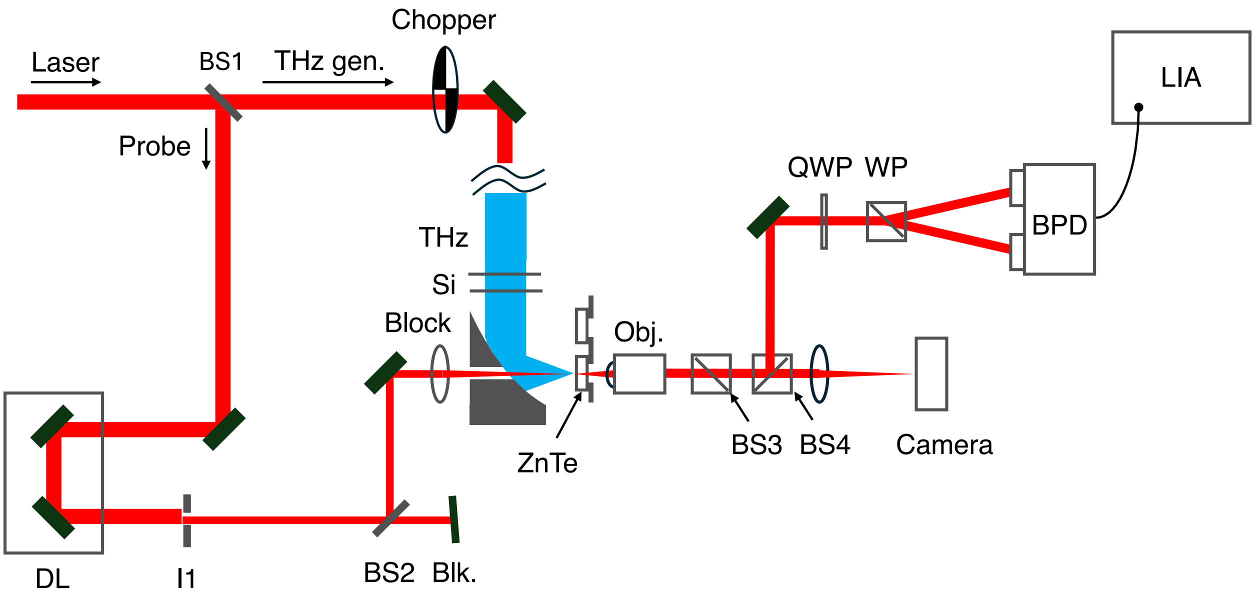

Supplementary Figure 48. Schematic optical setup for EO sampling [6]. In this measurement, the probe beam of 800 nm is used without conversion to the white light. The probe beam reflected at BS2 is focused on a 1-mm Zinc telluride (ZnTe) crystal at the focus of the THz beam. The probe beam reflected at BS4 passes through quarter-wave plate (QWP) and Wollaston prism (WP) to resolve the polarization and is detected at the balanced photodiode (BPD). The THz-generation beam is chopped at 500 Hz, and the signal from BPD is measured with lock-in amplifier (LIA). The mechanical delay line (DL) is used to sweep the time delay between probe pulse and THz pulse. The ZnTe and the 2D-3D hybrid THz antenna devices for the TPOP measurement were mounted on the same sample holder to locate the ZnTe and the antenna at the same position in the THz-beam focus in the two measurements. The EO-sampling probe beam transmitted BS4 was monitored by a camera to adjust the focus spot of the transmission probe beam of EO sampling to the focus spot of the reflection probe beam of TPOP measurement. The position of antenna devices was adjusted to this reflection probe spot in the TPOP measurement.

Supplementary Figure 48 shows the schematic setup for the EO sampling for the characterization of THz waveform.

Supplementary References

[1] Neu, J. & Schmuttenmaer, C. A. Tutorial: An introduction to terahertz time domain spectroscopy (THz-TDS). *J. Appl. Phys.* **124**, 231101 (2018).

[2] Glover, R. E. & Tinkham, M. Conductivity of superconducting films for photon energies between 0.3 and40kTc. *Phys. Rev.* **108**, 243–256 (1957).

[3] Born, M., Born, L. N. L., Wolf, E., Born, M. A. & Bhatia, A. B. *Principles of Optics: Electromagnetic Theory of Propagation, Interference and Diffraction of Light*. (Cambridge University Press, 1999).

[4] Walther, M. *et al.* Terahertz conductivity of thin gold films at the metal-insulator percolation transition. *Phys. Rev. B Condens. Matter* **76**, 125408 (2007).

[5] Robertson, J. High dielectric constant oxides. *Eur. Phys. J. - Appl. phys.* **28**, 265–291 (2004).

[6] Nahata, A., Weling, A. S. & Heinz, T. F. A wideband coherent terahertz spectroscopy system using optical rectification and electro-optic sampling. Appl. Phys. Lett. 69, 2321–2323 (1996).

[7] R. Olmon, B. Slovick, T. Johnson, D. Shelton, S.-H. Oh, G. Boreman, and M. Raschke, "Optical dielectric function of gold," Phys. Rev B. 86(23),235147 (2012).

[8] Z. Mei, S. Deng, L. Li, X. Wen, H. Lu, and M. Li, "Dielectric function of sub-10 nanometer thick gold films," Appl. Phys. A 127(6), 437 (2021).

[9] E. Palik, ed., *Handbook of optical constants of solids*, 3rd ed. (Academic press, 1998).

[10] Y. Yu, Y. Yu, Y. Cai, W. Li, A. Gurarslan, H. Peelaers, D. Aspnes, C. Van de Walle, N. Nguyen, Y.-W. Zhang, and L. Yu, "Exciton-dominated dielectric function of atomically thin MoS_2 films," Sci. Rep. 5(1), 16996 (2015).

[11] D. Marques, J. Guggenheim, R. Ansari, E. Zhang, P. Beard, and P. Munro, "Modelling Fabry-Pérot etalons illuminated by focussed beams," Opt. Express 28(5), 7691-7706 (2020).

[12] Radisavljevic, B. & Kis, A. Mobility engineering and a metal-insulator transition in monolayer MoS_2. *Nat. Mater.* **12**, 815–820 (2013).

[13] Mak, K. F. *et al.* Tightly bound trions in monolayer MoS_2. *Nat. Mater.* **12**, 207–211 (2013).

[14] Splendiani, A. *et al.* Emerging photoluminescence in monolayer MoS_2. *Nano Lett.* **10**, 1271–1275 (2010).

[15] Raja, A. *et al.* Dielectric disorder in two-dimensional materials. *Nat. Nanotechnol.* **14**, 832–837 (2019).

[16] Nie, Z. *et al.* Ultrafast carrier thermalization and cooling dynamics in few-layer MoS_2. *ACS Nano* **8**, 10931–10940 (2014).

Competing interests

Authors declare no competing interests.
