# Supplementary figures and images for: Terahertz field effect in a two-dimensional semiconductor
Source: Nat Commun. 2025 Jun 5;16:5235. doi: 10.1038/s41467-025-60588-6 (PMC12141635; doi:10.1038/s41467-025-60588-6)

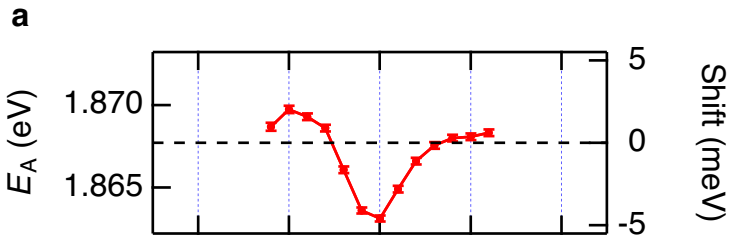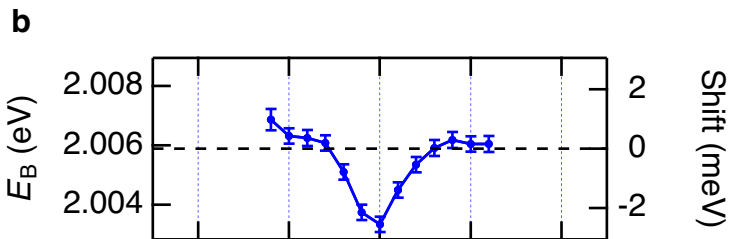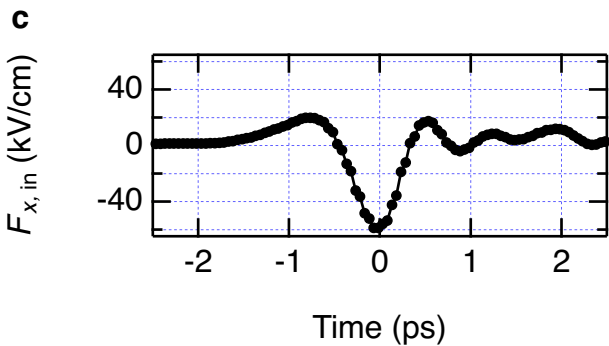

Supplement: Supplementary file 4 — Supplementary Data 1. Figures showing results of all the TPOP measurements and their analysis. For the figure legend of each figure, refer to Supplementary Notes 6. [file 41467_2025_60588_MOESM4_ESM.zip › SupplementaryFigureFiles/deviceII_meas1_180deg/l20240221_tpop412_REtFit_ParamEshiftVsTime.pdf]

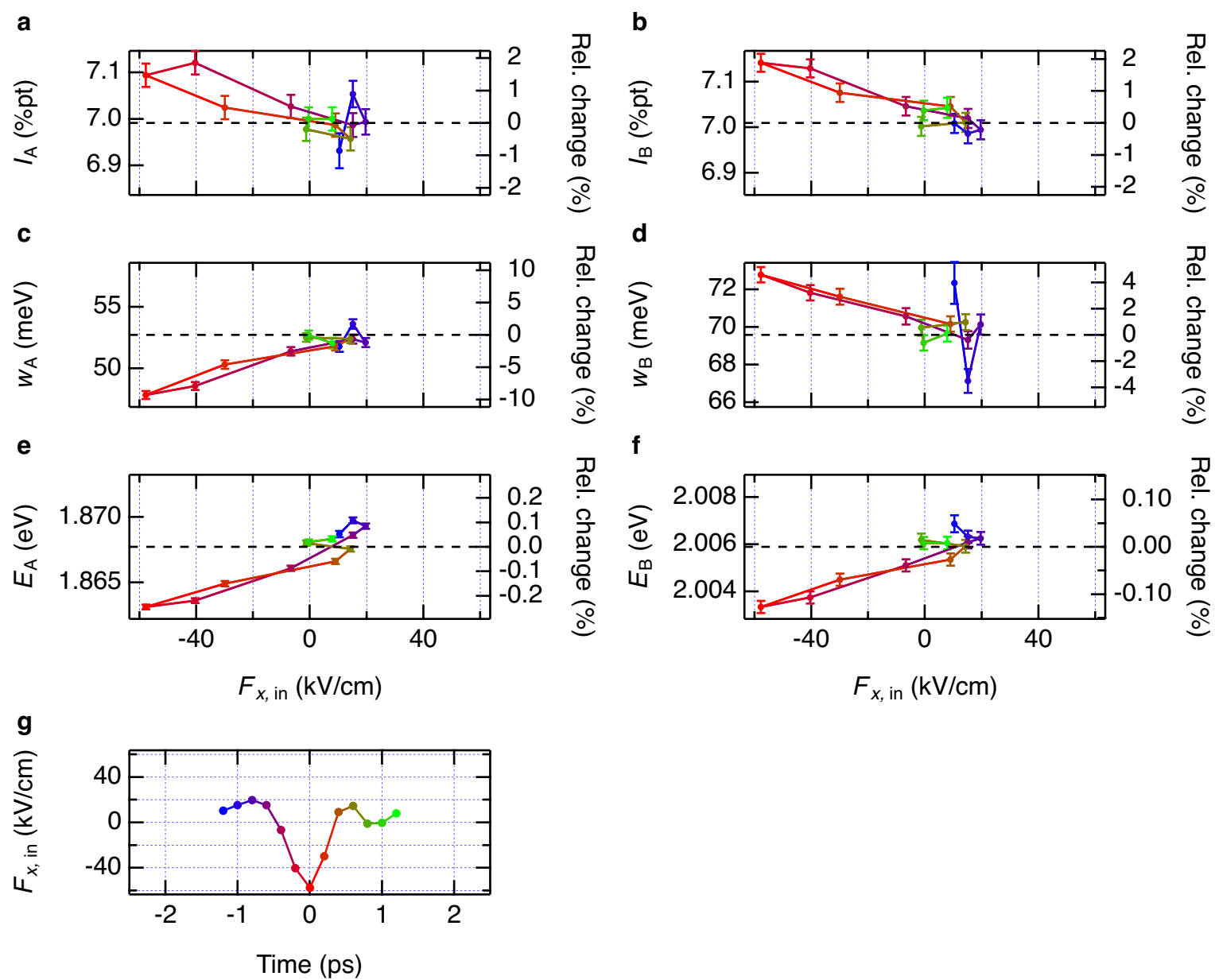

Supplement: Supplementary file 4 — Supplementary Data 1. Figures showing results of all the TPOP measurements and their analysis. For the figure legend of each figure, refer to Supplementary Notes 6. [file 41467_2025_60588_MOESM4_ESM.zip › SupplementaryFigureFiles/deviceII_meas1_180deg/l20240221_tpop412_REtFit_ParamVsETHz.pdf]

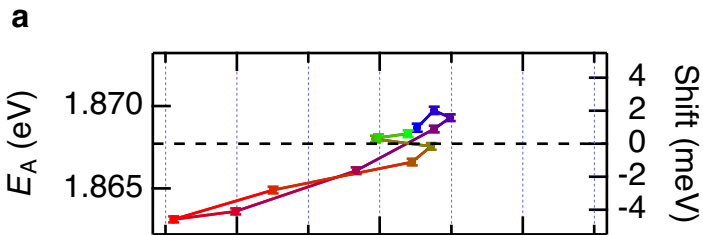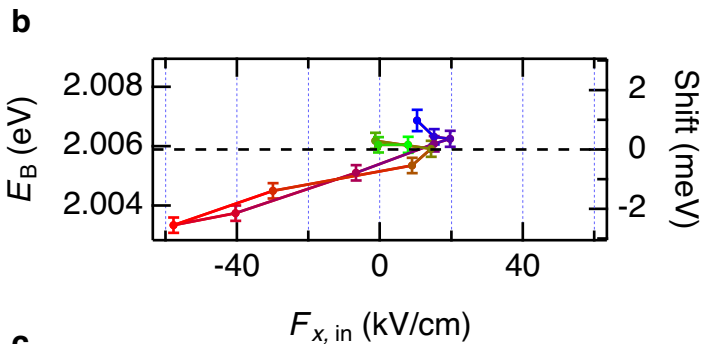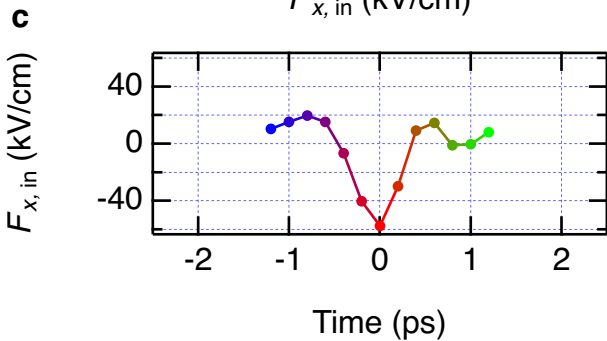

Supplement: Supplementary file 4 — Supplementary Data 1. Figures showing results of all the TPOP measurements and their analysis. For the figure legend of each figure, refer to Supplementary Notes 6. [file 41467_2025_60588_MOESM4_ESM.zip › SupplementaryFigureFiles/deviceII_meas1_180deg/l20240221_tpop412_REtFit_ParamEshiftVsETHz.pdf]

**a**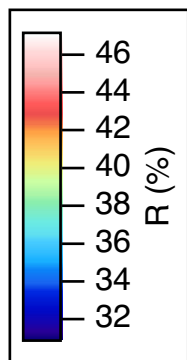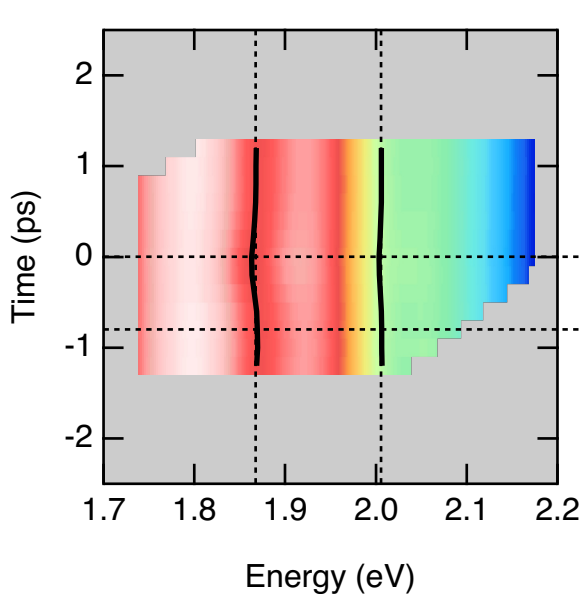**b**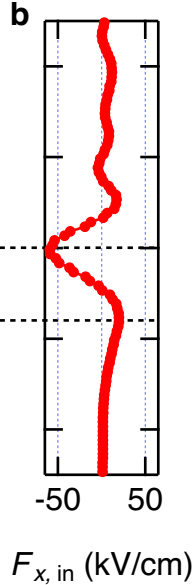**c**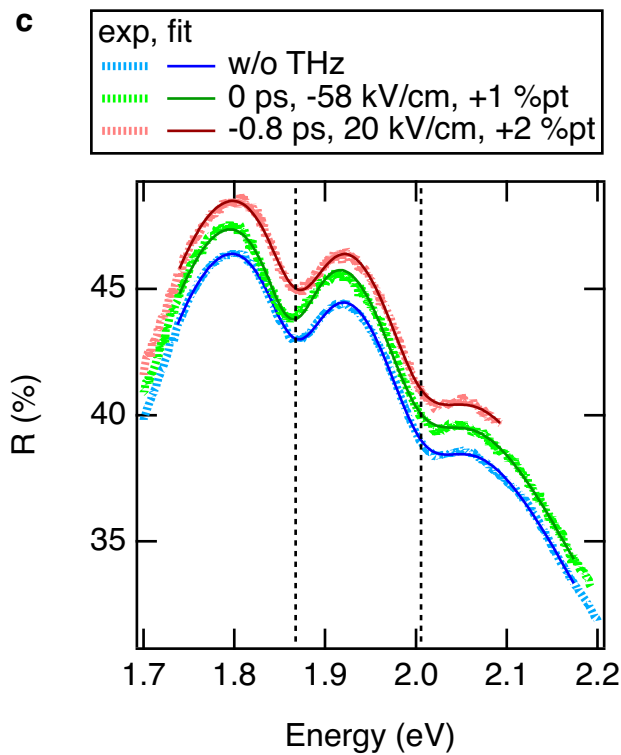

Supplement: Supplementary file 4 — Supplementary Data 1. Figures showing results of all the TPOP measurements and their analysis. For the figure legend of each figure, refer to Supplementary Notes 6. [file 41467_2025_60588_MOESM4_ESM.zip › SupplementaryFigureFiles/deviceII_meas1_180deg/l20240221_tpop412_REtFit_and_EOS.pdf]

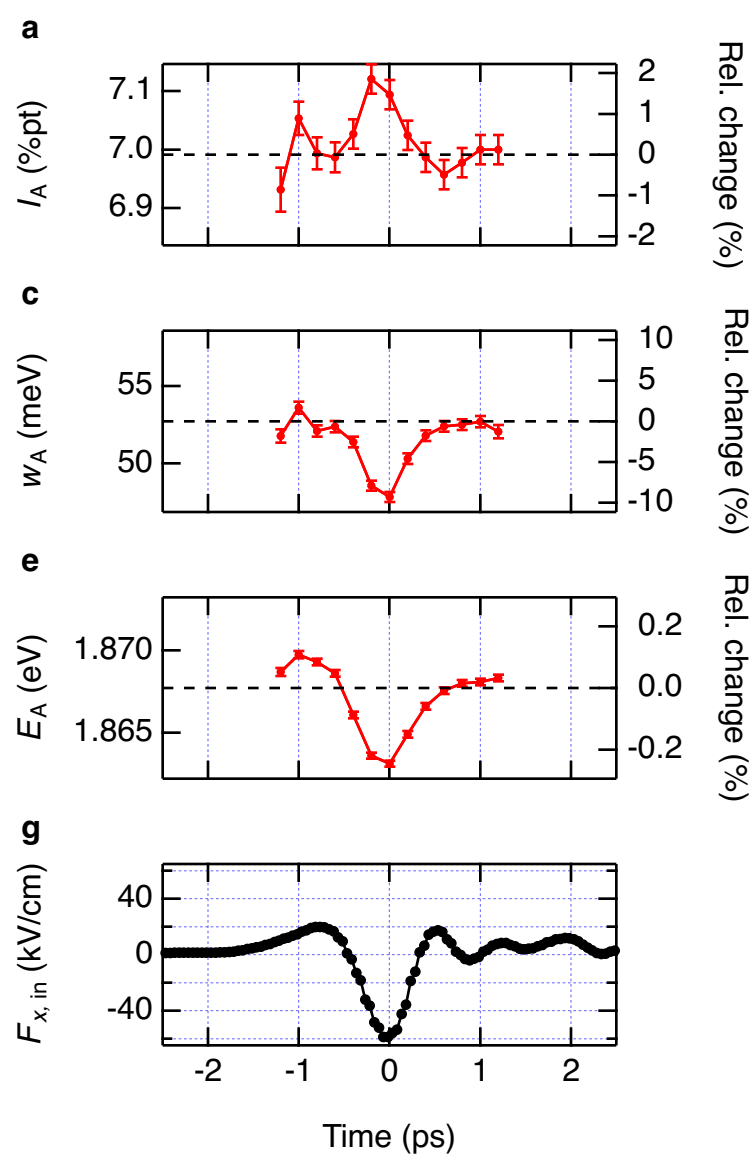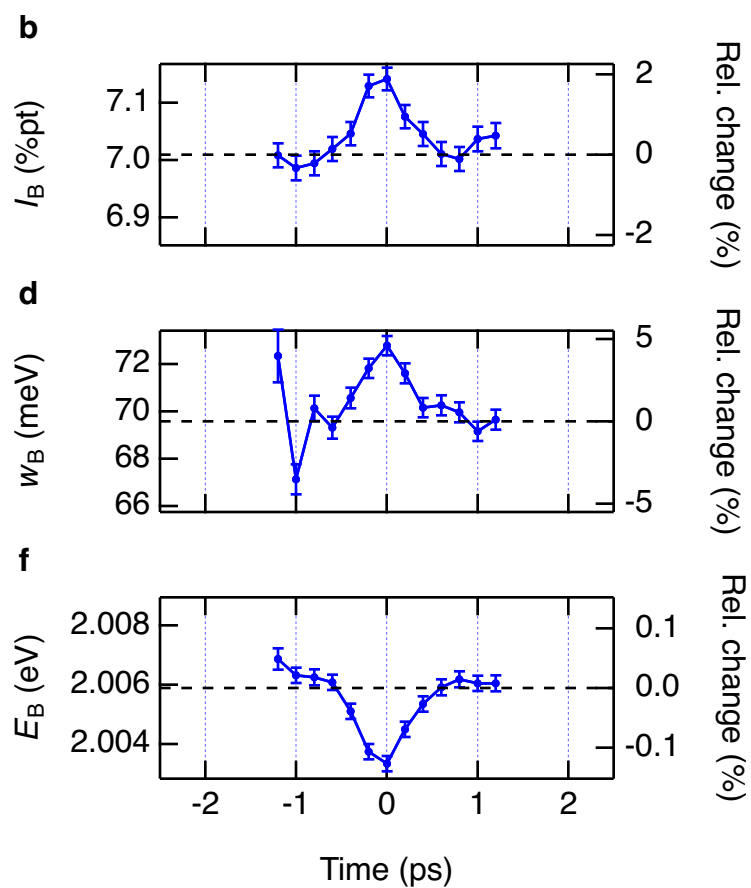

Supplement: Supplementary file 4 — Supplementary Data 1. Figures showing results of all the TPOP measurements and their analysis. For the figure legend of each figure, refer to Supplementary Notes 6. [file 41467_2025_60588_MOESM4_ESM.zip › SupplementaryFigureFiles/deviceII_meas1_180deg/l20240221_tpop412_REtFit_ParamVsTime.pdf]

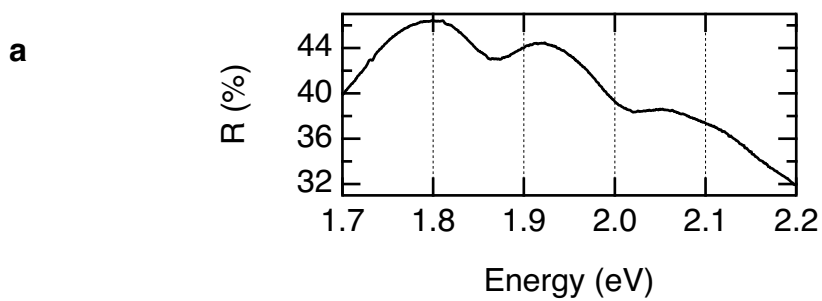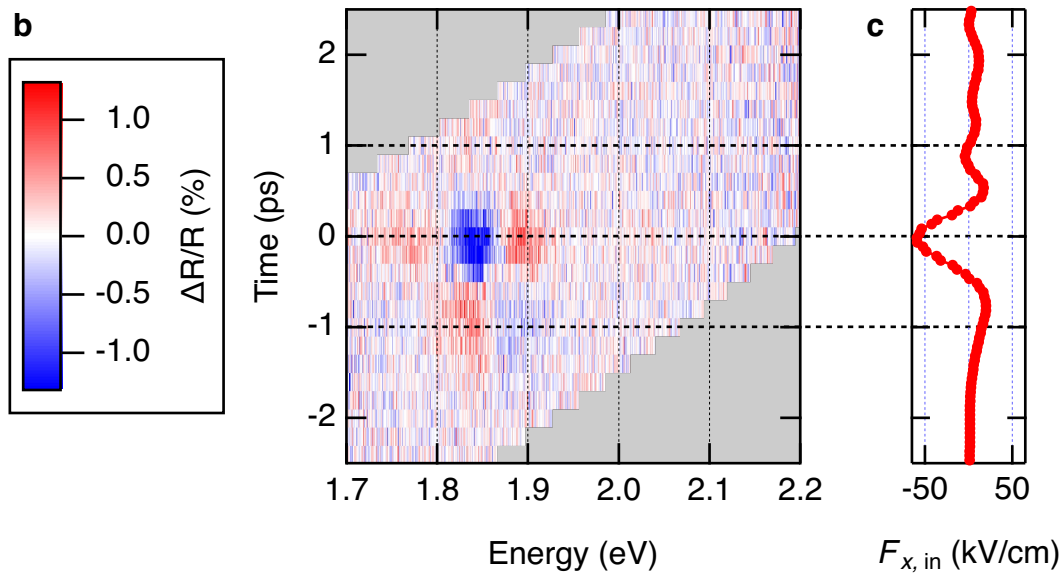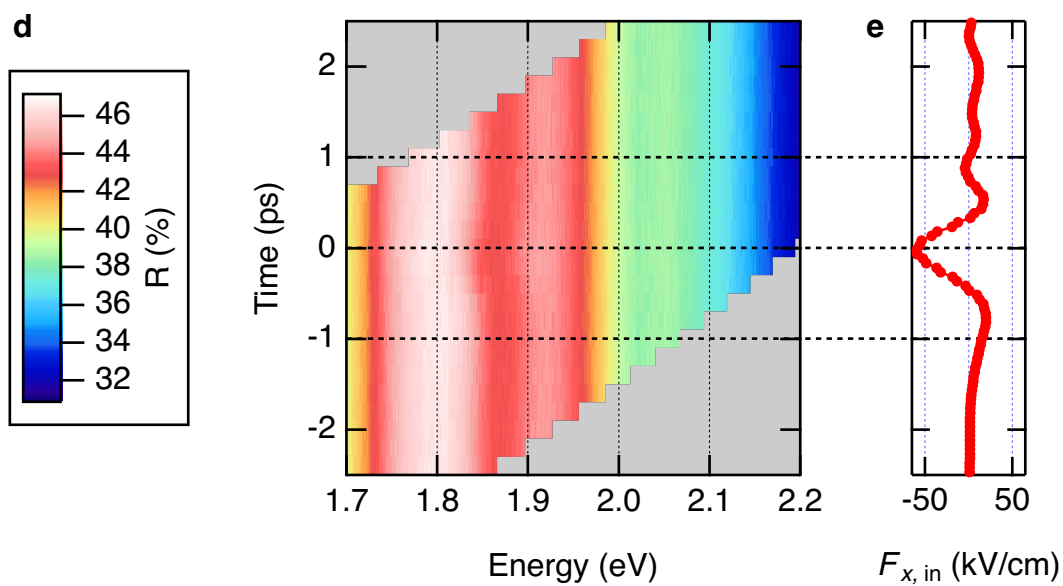

Supplement: Supplementary file 4 — Supplementary Data 1. Figures showing results of all the TPOP measurements and their analysis. For the figure legend of each figure, refer to Supplementary Notes 6. [file 41467_2025_60588_MOESM4_ESM.zip › SupplementaryFigureFiles/deviceII_meas1_180deg/l20240221_tpop412_summary_measTPOPdata.pdf]

**a**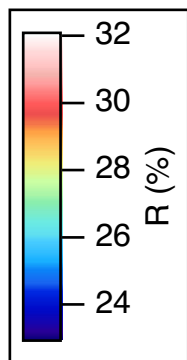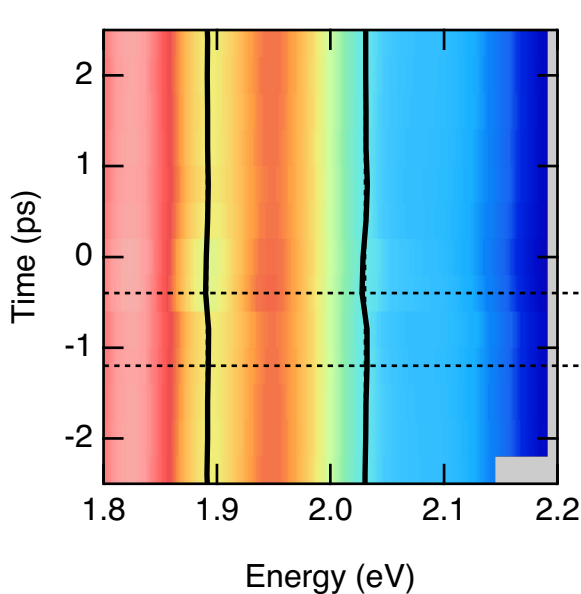**b**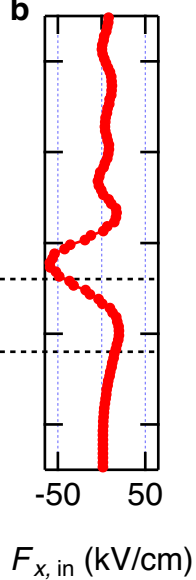**c**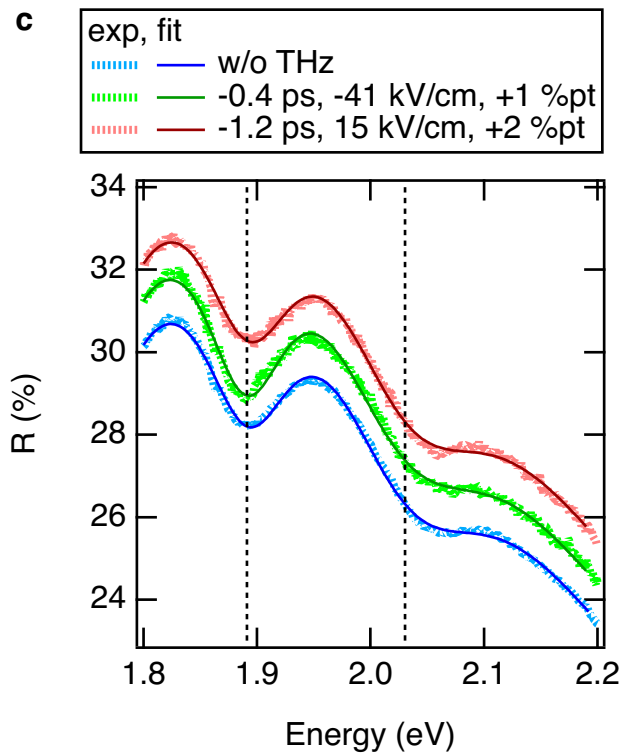

Supplement: Supplementary file 4 — Supplementary Data 1. Figures showing results of all the TPOP measurements and their analysis. For the figure legend of each figure, refer to Supplementary Notes 6. [file 41467_2025_60588_MOESM4_ESM.zip › SupplementaryFigureFiles/deviceI_meas2_180deg/l20240221_tpop11_REtFit_and_EOS.pdf]

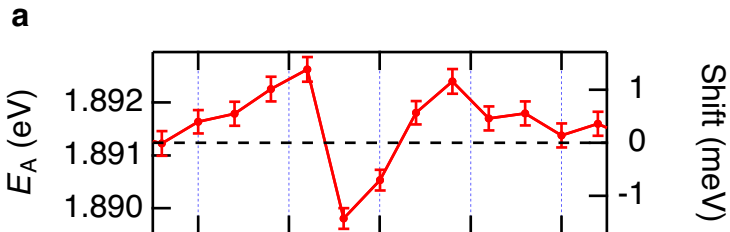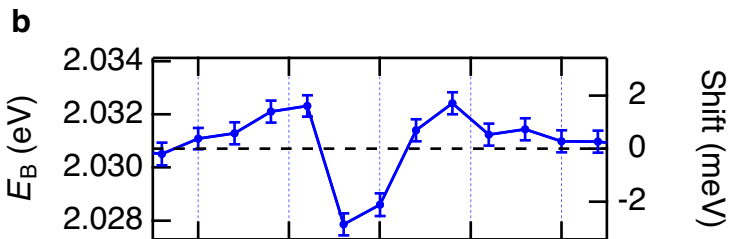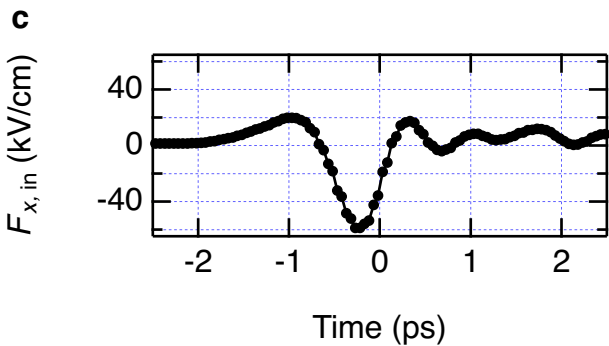

Supplement: Supplementary file 4 — Supplementary Data 1. Figures showing results of all the TPOP measurements and their analysis. For the figure legend of each figure, refer to Supplementary Notes 6. [file 41467_2025_60588_MOESM4_ESM.zip › SupplementaryFigureFiles/deviceI_meas2_180deg/l20240221_tpop11_REtFit_ParamEshiftVsTime.pdf]

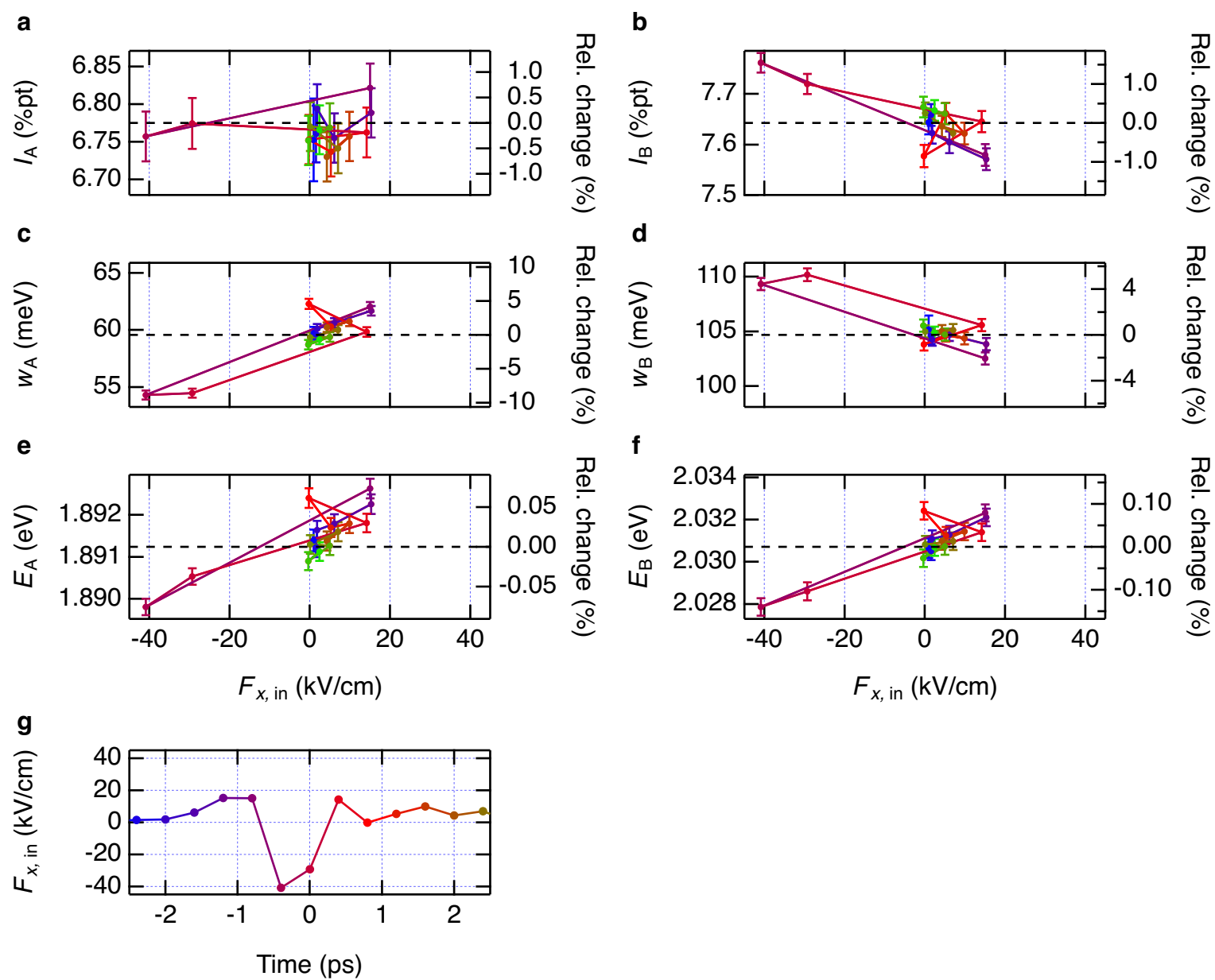

Supplement: Supplementary file 4 — Supplementary Data 1. Figures showing results of all the TPOP measurements and their analysis. For the figure legend of each figure, refer to Supplementary Notes 6. [file 41467_2025_60588_MOESM4_ESM.zip › SupplementaryFigureFiles/deviceI_meas2_180deg/l20240221_tpop11_REtFit_ParamVsETHz.pdf]

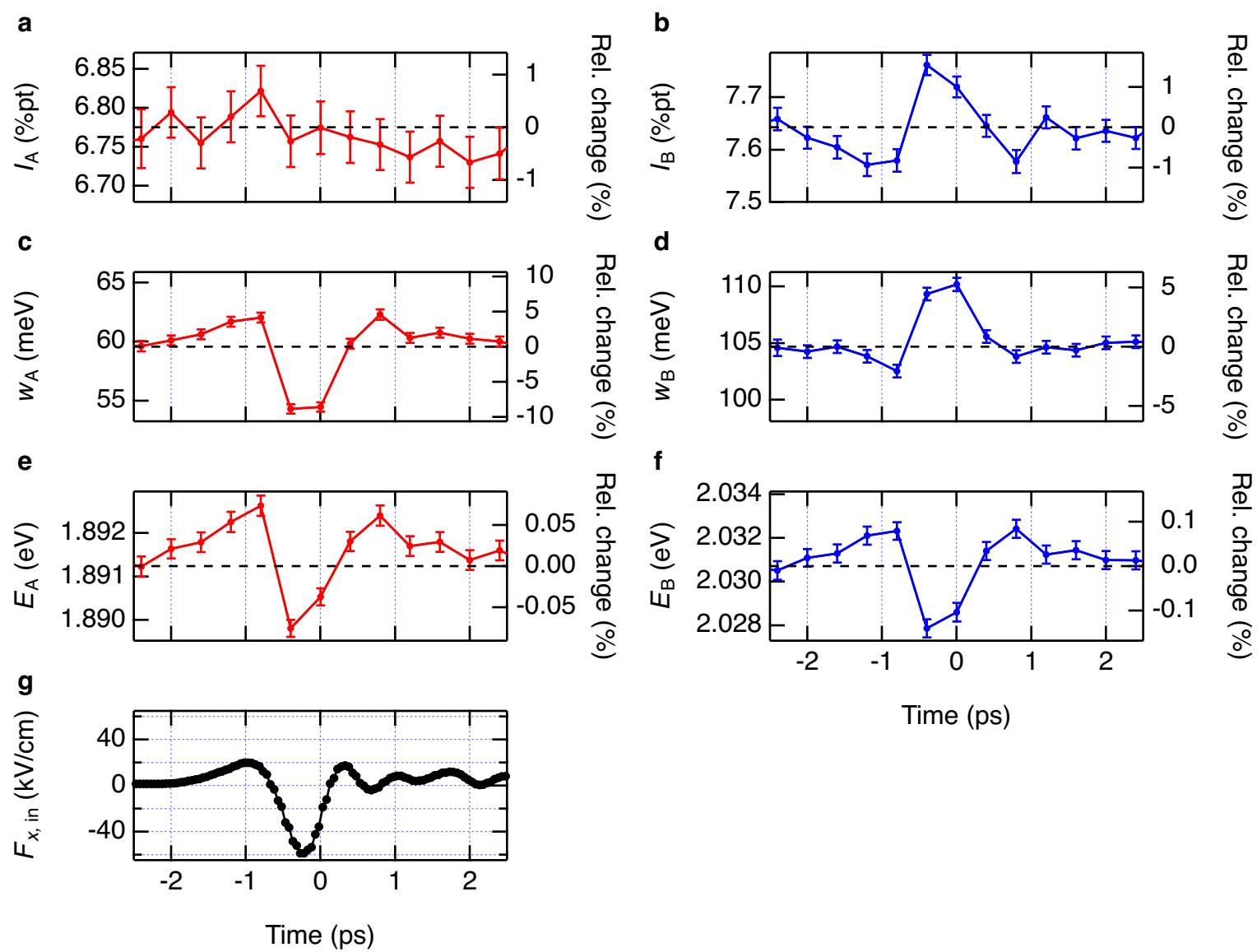

Supplement: Supplementary file 4 — Supplementary Data 1. Figures showing results of all the TPOP measurements and their analysis. For the figure legend of each figure, refer to Supplementary Notes 6. [file 41467_2025_60588_MOESM4_ESM.zip › SupplementaryFigureFiles/deviceI_meas2_180deg/l20240221_tpop11_REtFit_ParamVsTime.pdf]

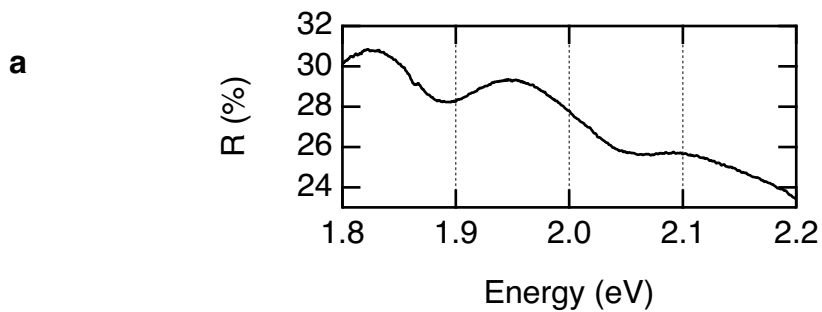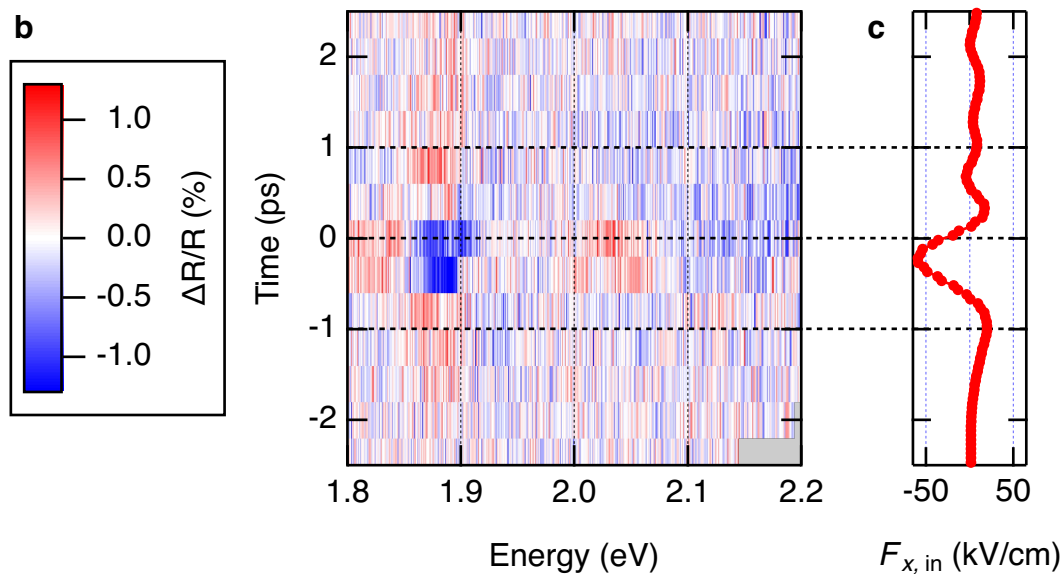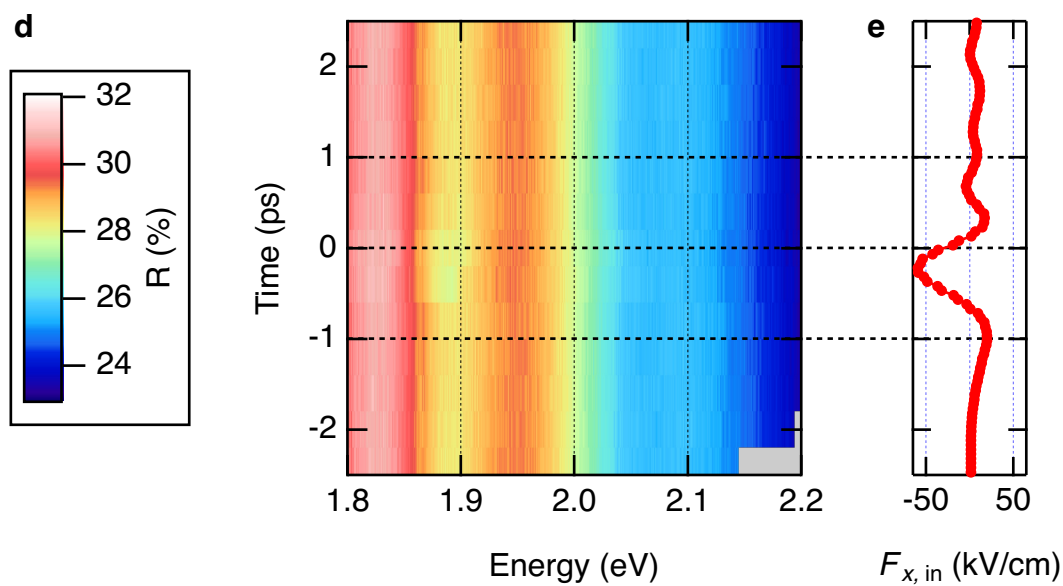

Supplement: Supplementary file 4 — Supplementary Data 1. Figures showing results of all the TPOP measurements and their analysis. For the figure legend of each figure, refer to Supplementary Notes 6. [file 41467_2025_60588_MOESM4_ESM.zip › SupplementaryFigureFiles/deviceI_meas2_180deg/l20240221_tpop11_summary_measTPOPdata.pdf]

**a**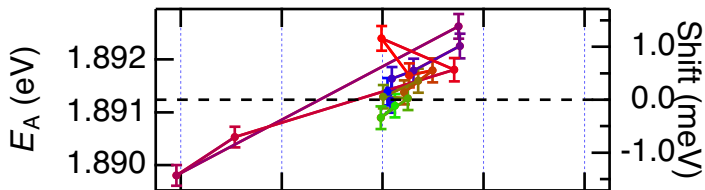**b**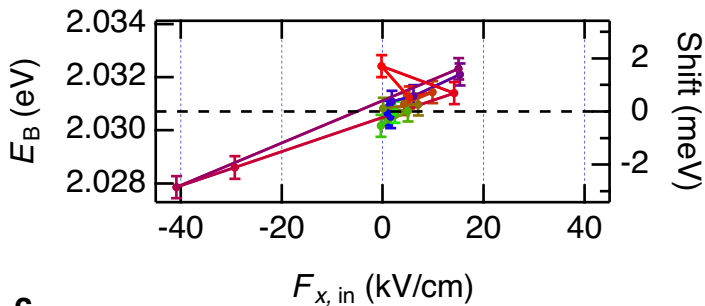**c**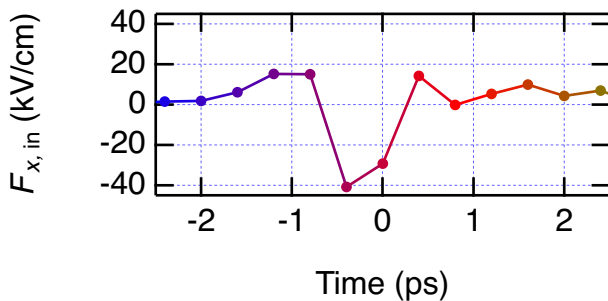

Supplement: Supplementary file 4 — Supplementary Data 1. Figures showing results of all the TPOP measurements and their analysis. For the figure legend of each figure, refer to Supplementary Notes 6. [file 41467_2025_60588_MOESM4_ESM.zip › SupplementaryFigureFiles/deviceI_meas2_180deg/l20240221_tpop11_REtFit_ParamEshiftVsETHz.pdf]

**a**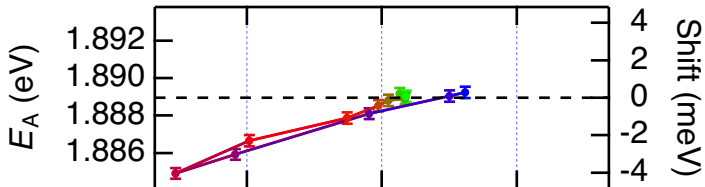**b**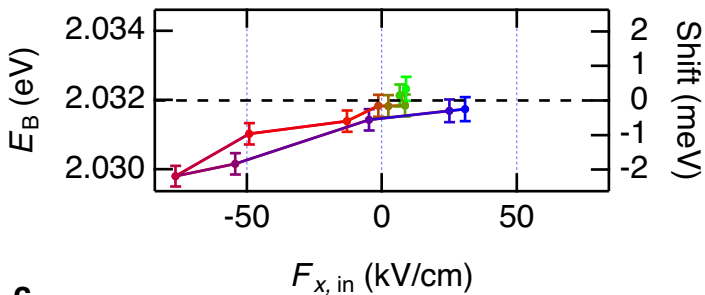**c**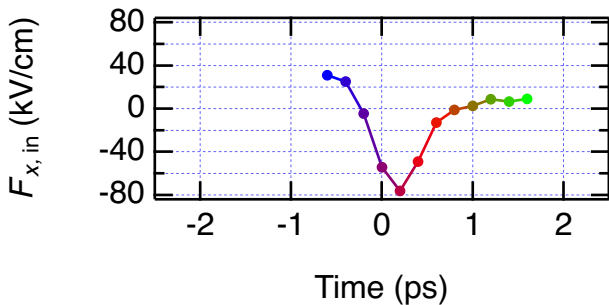

Supplement: Supplementary file 4 — Supplementary Data 1. Figures showing results of all the TPOP measurements and their analysis. For the figure legend of each figure, refer to Supplementary Notes 6. [file 41467_2025_60588_MOESM4_ESM.zip › SupplementaryFigureFiles/deviceIII_meas2_180deg/l20240222_tpop13_REtFit_ParamEshiftVsETHz.pdf]

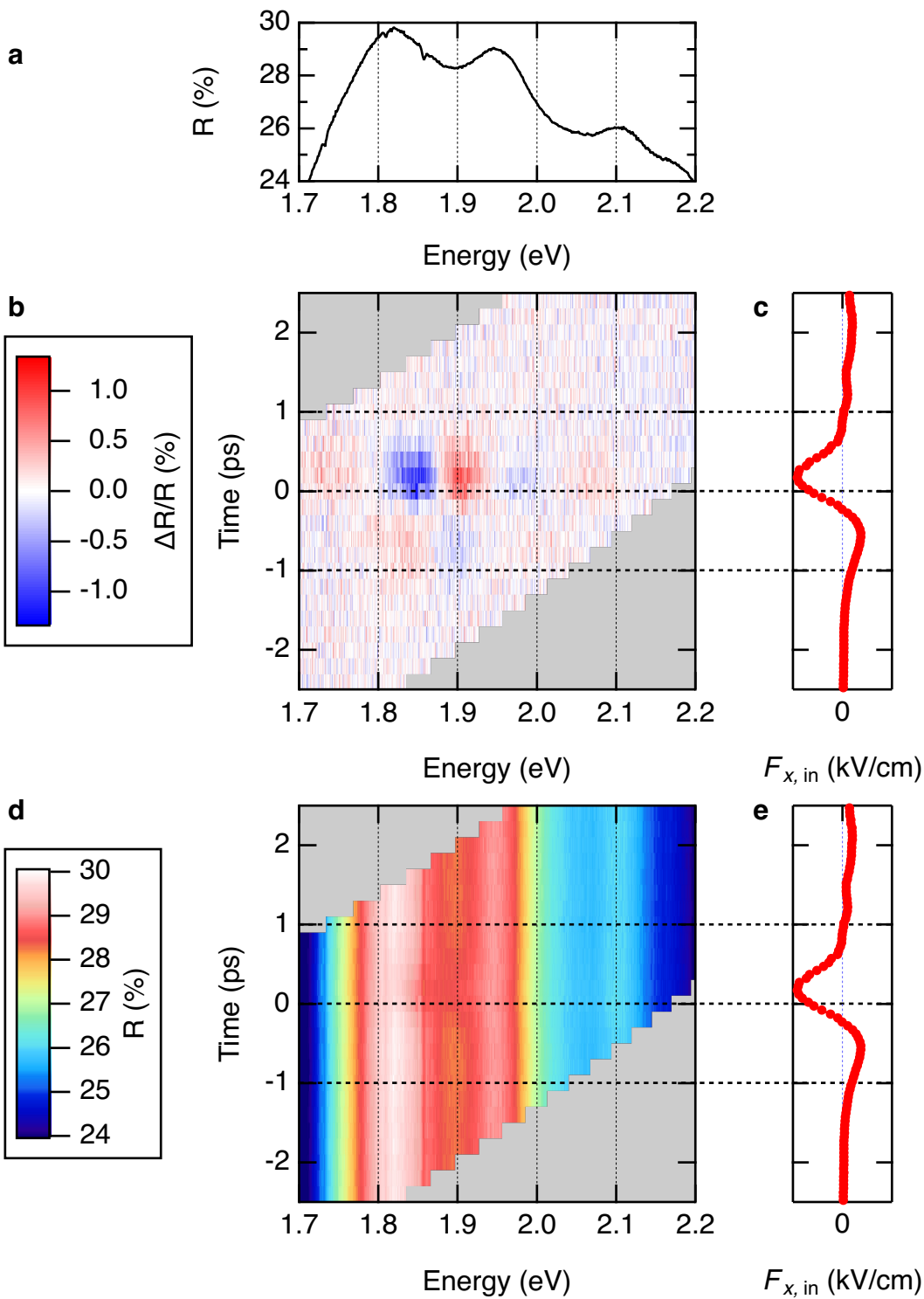

Supplement: Supplementary file 4 — Supplementary Data 1. Figures showing results of all the TPOP measurements and their analysis. For the figure legend of each figure, refer to Supplementary Notes 6. [file 41467_2025_60588_MOESM4_ESM.zip › SupplementaryFigureFiles/deviceIII_meas2_180deg/l20240222_tpop13_summary_measTPOPdata.pdf]

**a**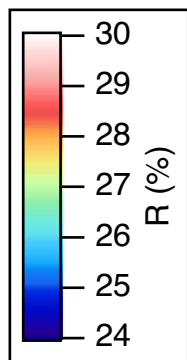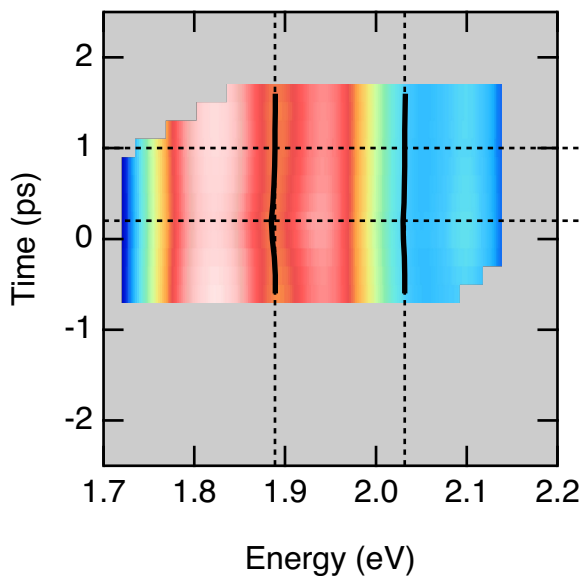**b**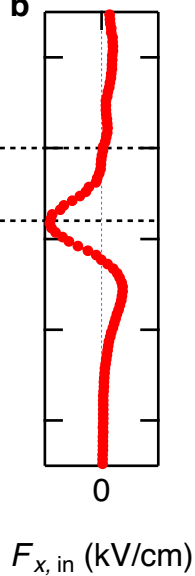**c**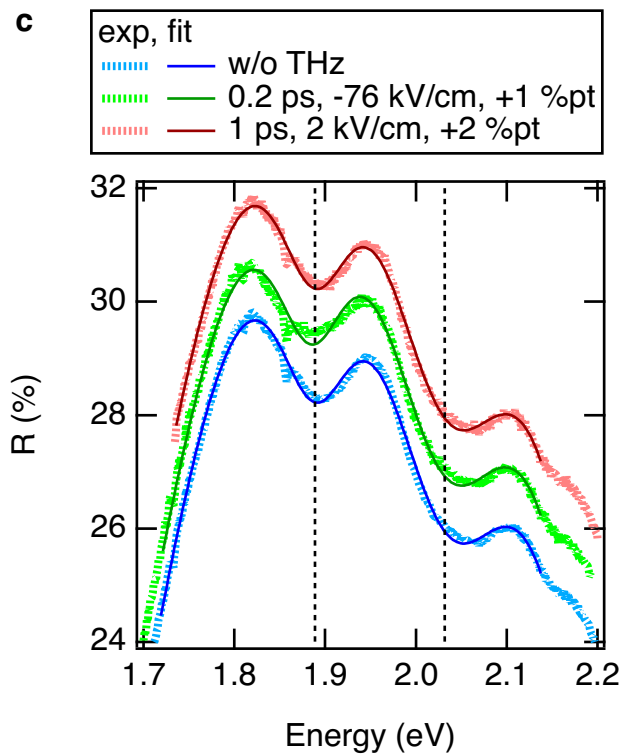

Supplement: Supplementary file 4 — Supplementary Data 1. Figures showing results of all the TPOP measurements and their analysis. For the figure legend of each figure, refer to Supplementary Notes 6. [file 41467_2025_60588_MOESM4_ESM.zip › SupplementaryFigureFiles/deviceIII_meas2_180deg/l20240222_tpop13_REtFit_and_EOS.pdf]

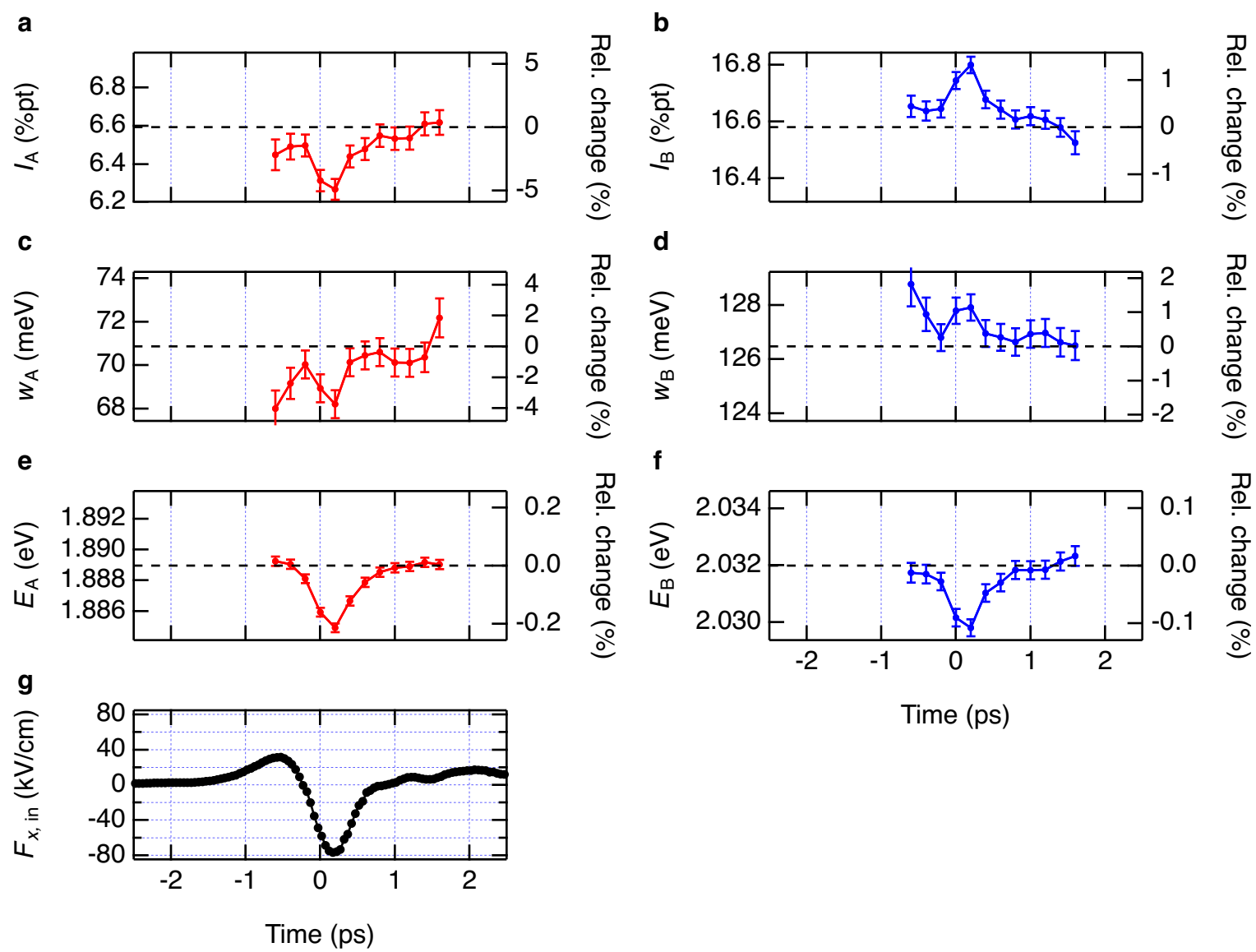

Supplement: Supplementary file 4 — Supplementary Data 1. Figures showing results of all the TPOP measurements and their analysis. For the figure legend of each figure, refer to Supplementary Notes 6. [file 41467_2025_60588_MOESM4_ESM.zip › SupplementaryFigureFiles/deviceIII_meas2_180deg/l20240222_tpop13_REtFit_ParamVsTime.pdf]

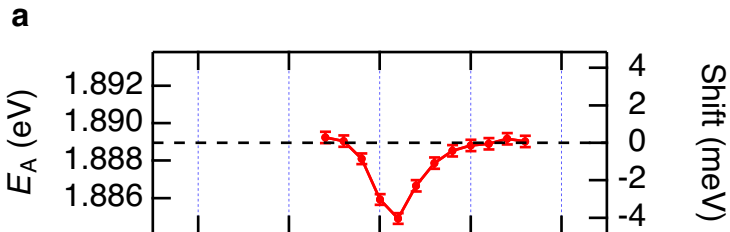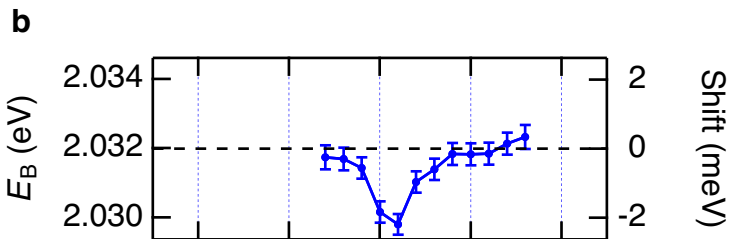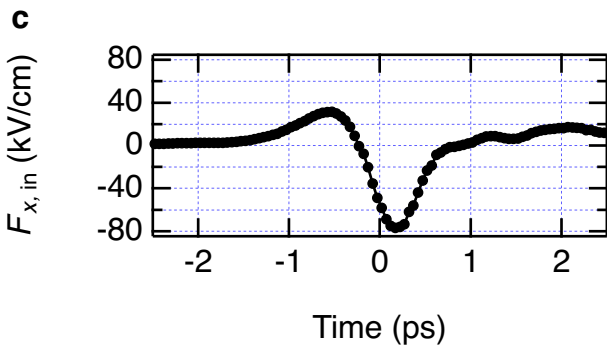

Supplement: Supplementary file 4 — Supplementary Data 1. Figures showing results of all the TPOP measurements and their analysis. For the figure legend of each figure, refer to Supplementary Notes 6. [file 41467_2025_60588_MOESM4_ESM.zip › SupplementaryFigureFiles/deviceIII_meas2_180deg/l20240222_tpop13_REtFit_ParamEshiftVsTime.pdf]

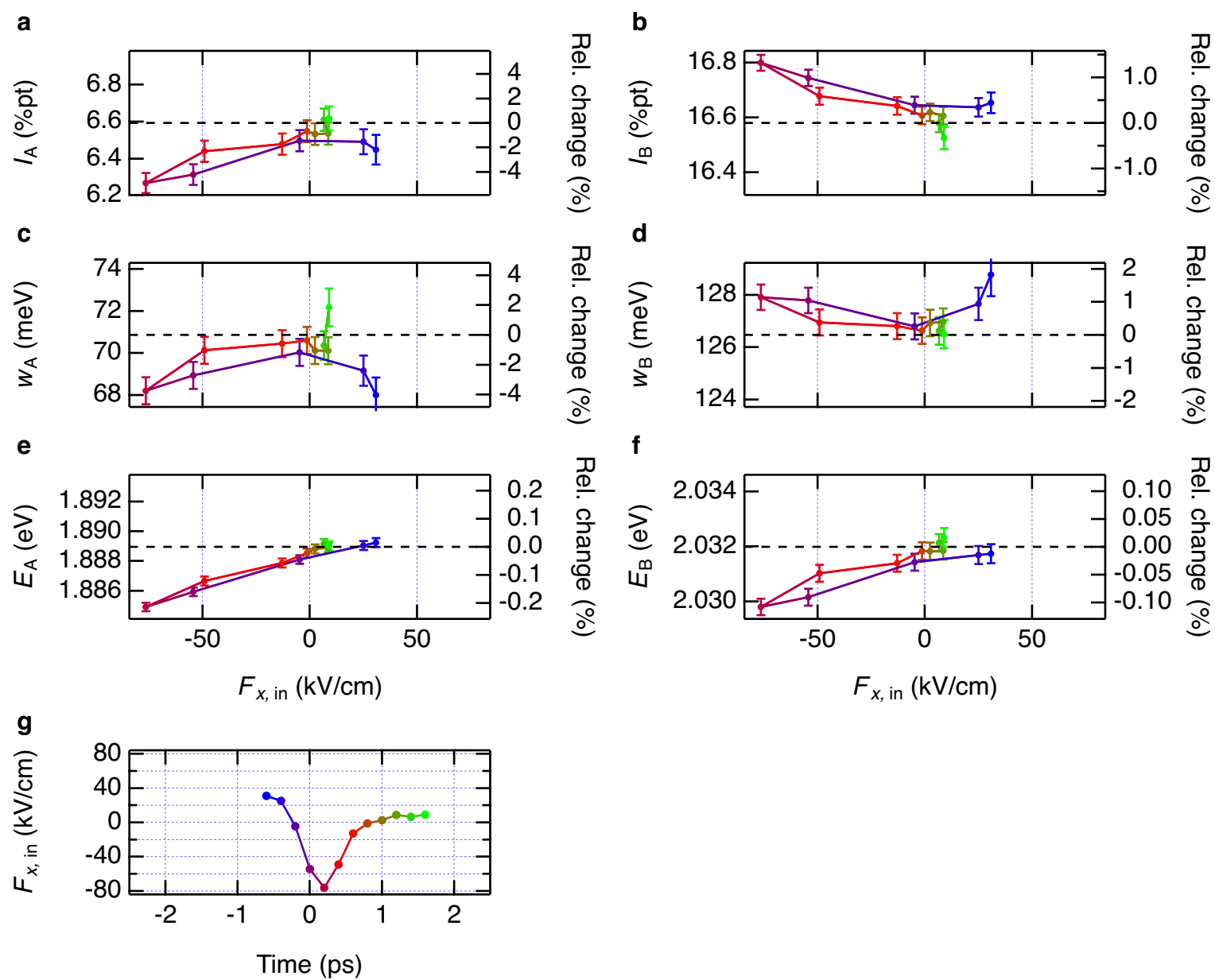

Supplement: Supplementary file 4 — Supplementary Data 1. Figures showing results of all the TPOP measurements and their analysis. For the figure legend of each figure, refer to Supplementary Notes 6. [file 41467_2025_60588_MOESM4_ESM.zip › SupplementaryFigureFiles/deviceIII_meas2_180deg/l20240222_tpop13_REtFit_ParamVsETHz.pdf]

**a**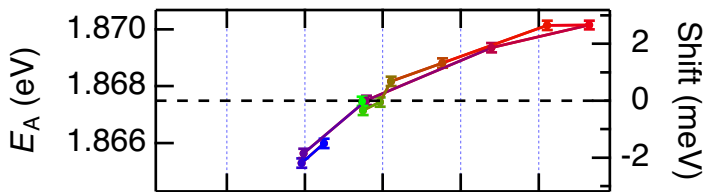**b**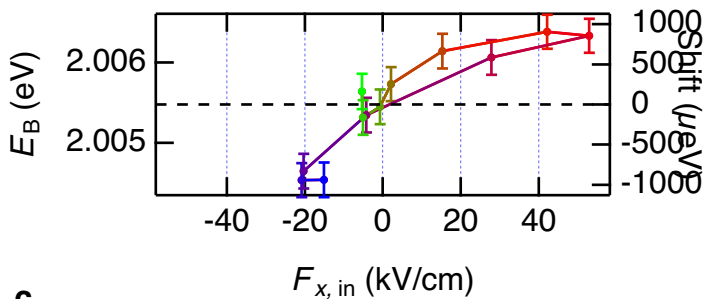**c**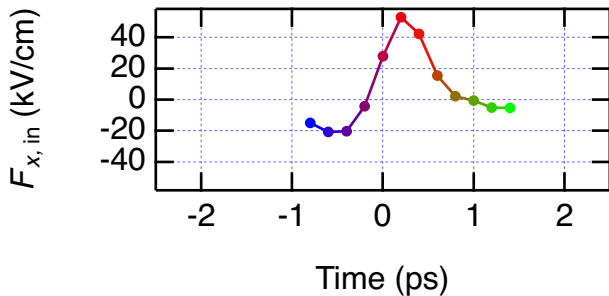

Supplement: Supplementary file 4 — Supplementary Data 1. Figures showing results of all the TPOP measurements and their analysis. For the figure legend of each figure, refer to Supplementary Notes 6. [file 41467_2025_60588_MOESM4_ESM.zip › SupplementaryFigureFiles/deviceII_meas3_0deg/l20240222_tpop22_REtFit_ParamEshiftVsETHz.pdf]

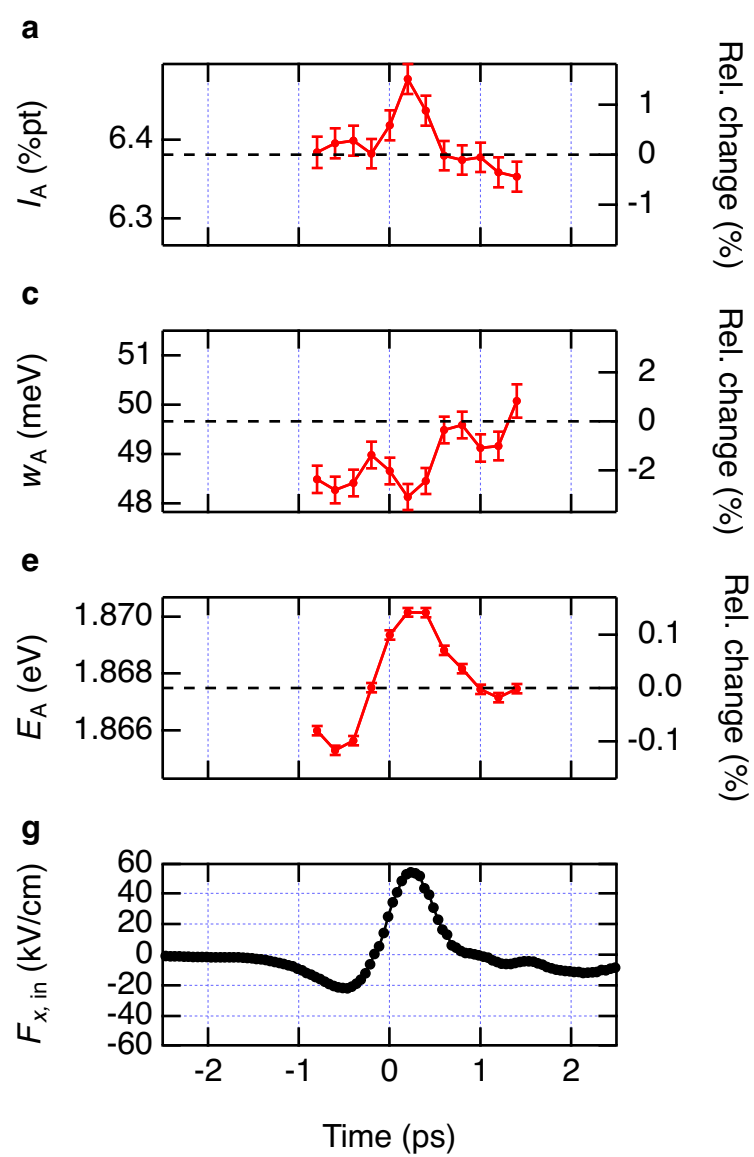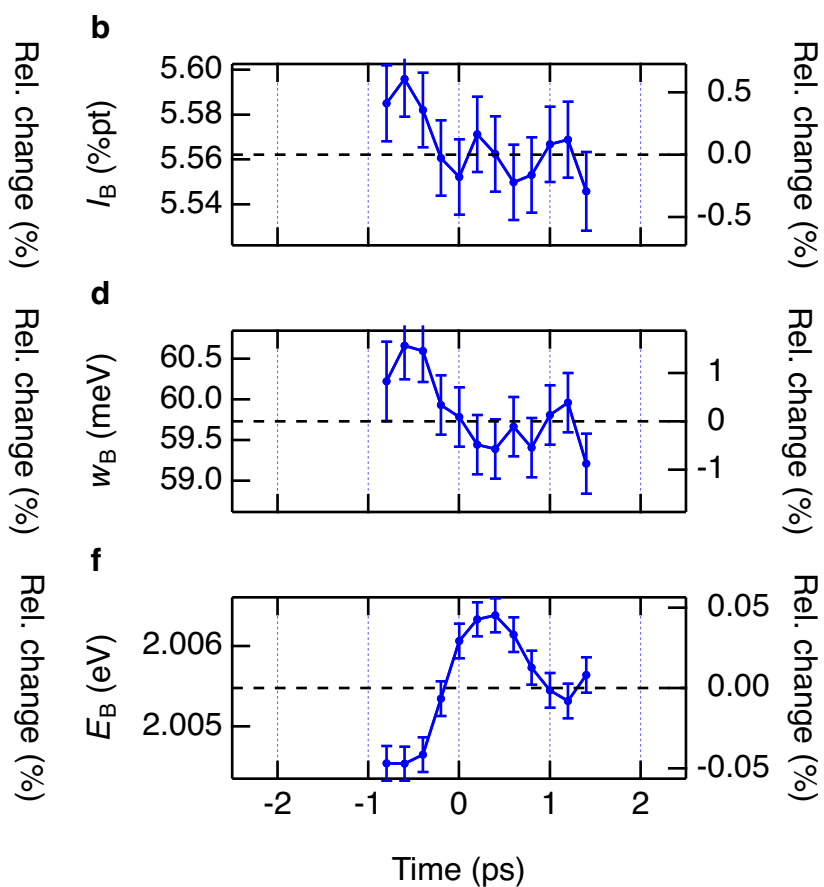

Supplement: Supplementary file 4 — Supplementary Data 1. Figures showing results of all the TPOP measurements and their analysis. For the figure legend of each figure, refer to Supplementary Notes 6. [file 41467_2025_60588_MOESM4_ESM.zip › SupplementaryFigureFiles/deviceII_meas3_0deg/l20240222_tpop22_REtFit_ParamVsTime.pdf]

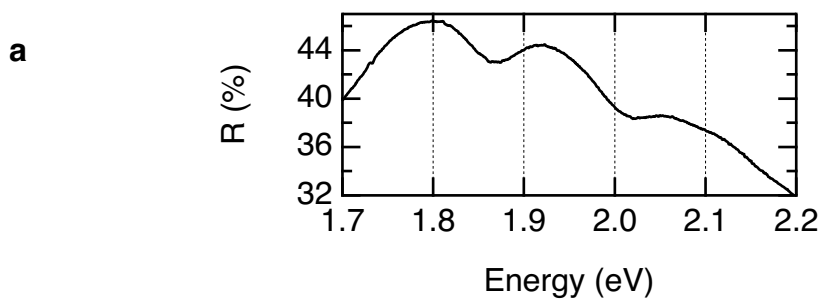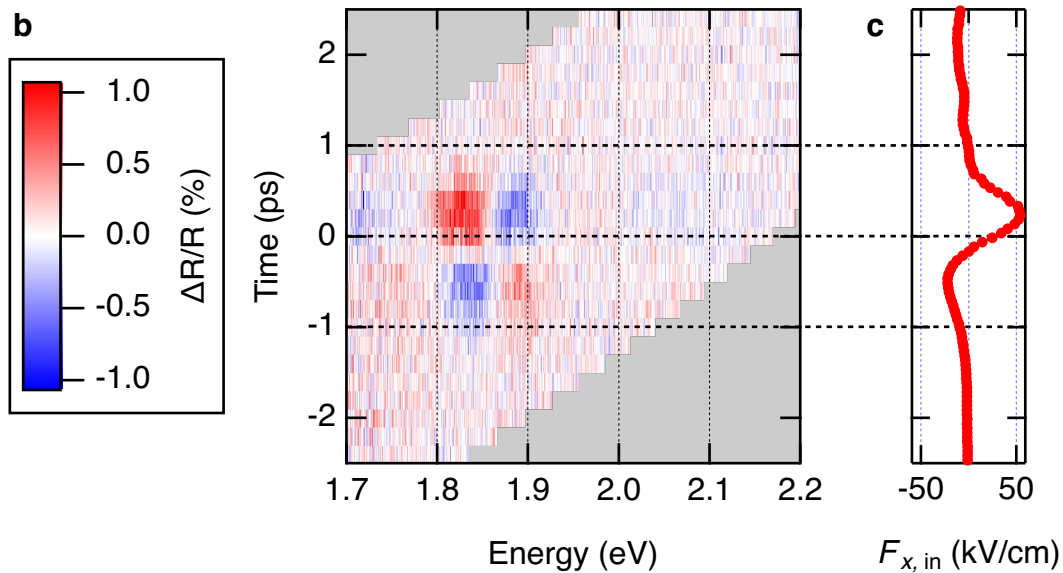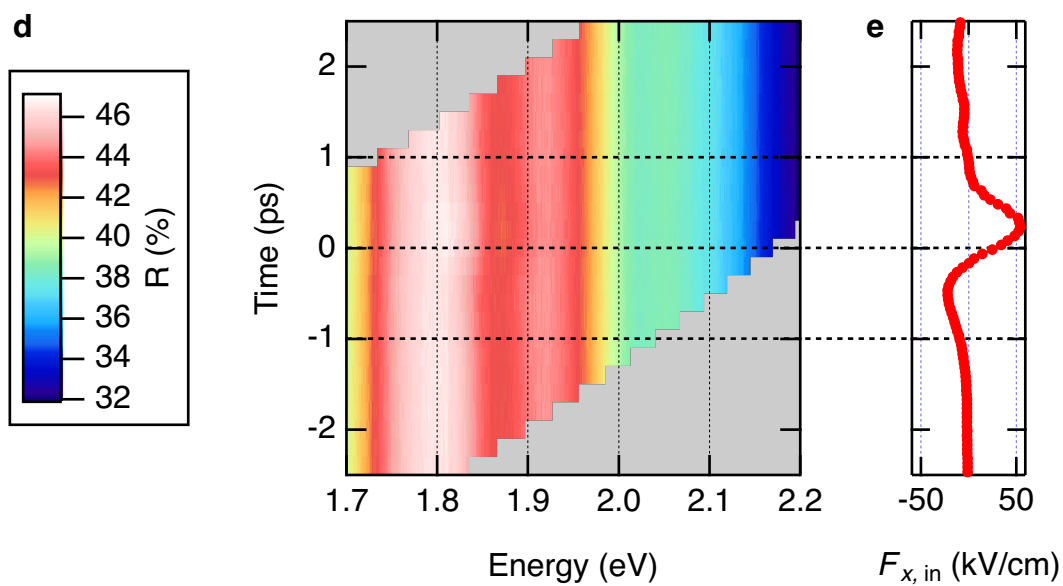

Supplement: Supplementary file 4 — Supplementary Data 1. Figures showing results of all the TPOP measurements and their analysis. For the figure legend of each figure, refer to Supplementary Notes 6. [file 41467_2025_60588_MOESM4_ESM.zip › SupplementaryFigureFiles/deviceII_meas3_0deg/l20240222_tpop22_summary_measTPOPdata.pdf]

**a**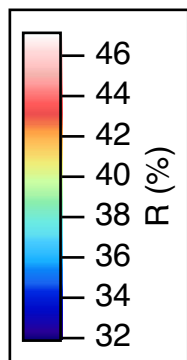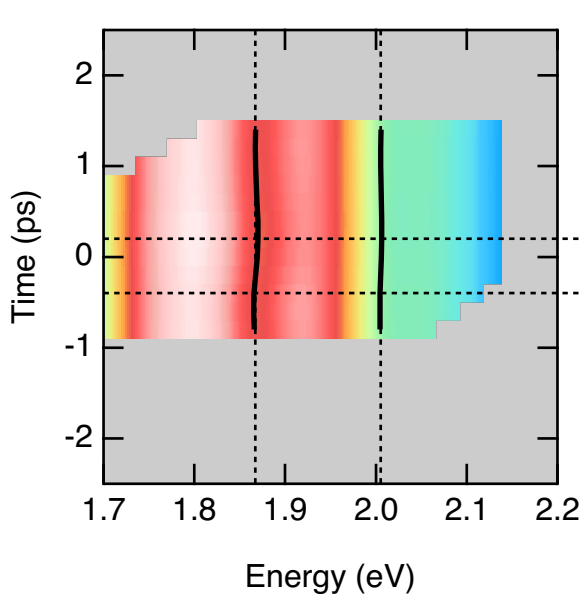**b**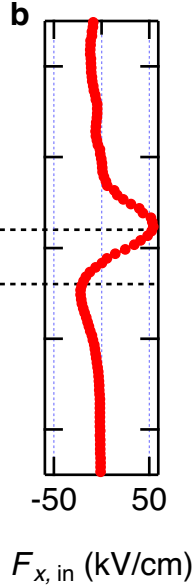**c**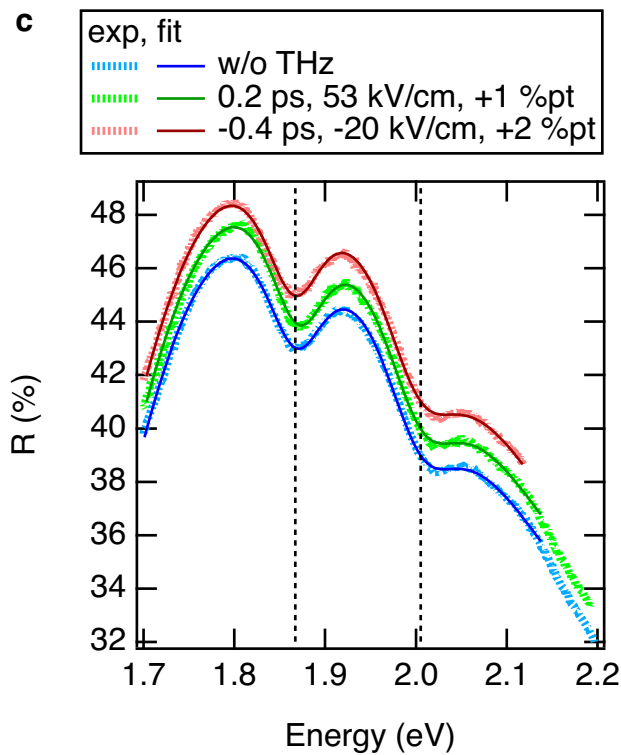

Supplement: Supplementary file 4 — Supplementary Data 1. Figures showing results of all the TPOP measurements and their analysis. For the figure legend of each figure, refer to Supplementary Notes 6. [file 41467_2025_60588_MOESM4_ESM.zip › SupplementaryFigureFiles/deviceII_meas3_0deg/l20240222_tpop22_REtFit_and_EOS.pdf]

**a**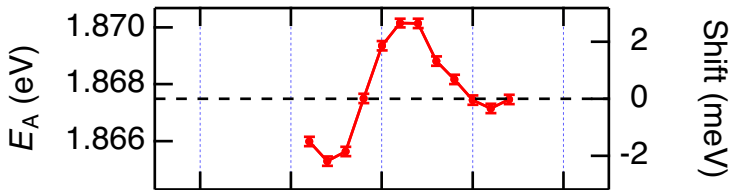**b**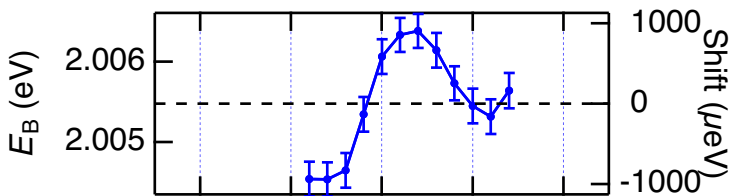**c**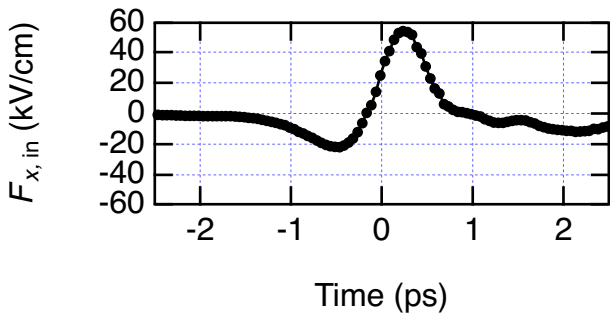

Supplement: Supplementary file 4 — Supplementary Data 1. Figures showing results of all the TPOP measurements and their analysis. For the figure legend of each figure, refer to Supplementary Notes 6. [file 41467_2025_60588_MOESM4_ESM.zip › SupplementaryFigureFiles/deviceII_meas3_0deg/l20240222_tpop22_REtFit_ParamEshiftVsTime.pdf]

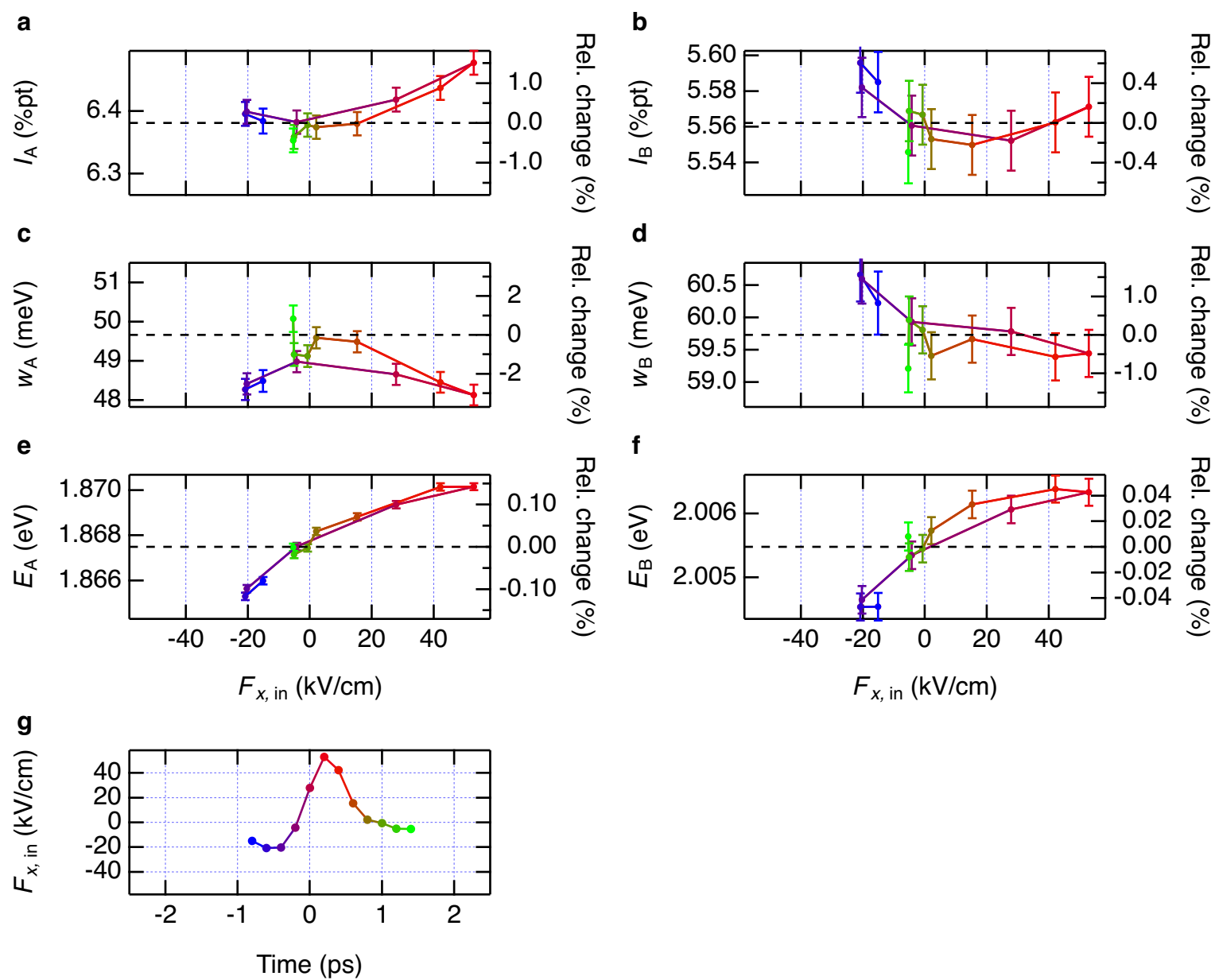

Supplement: Supplementary file 4 — Supplementary Data 1. Figures showing results of all the TPOP measurements and their analysis. For the figure legend of each figure, refer to Supplementary Notes 6. [file 41467_2025_60588_MOESM4_ESM.zip › SupplementaryFigureFiles/deviceII_meas3_0deg/l20240222_tpop22_REtFit_ParamVsETHz.pdf]

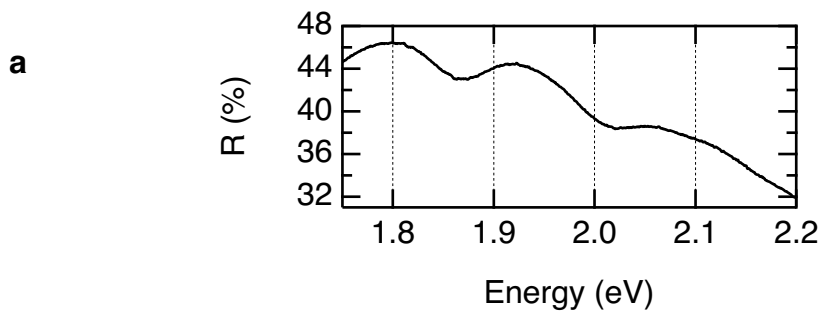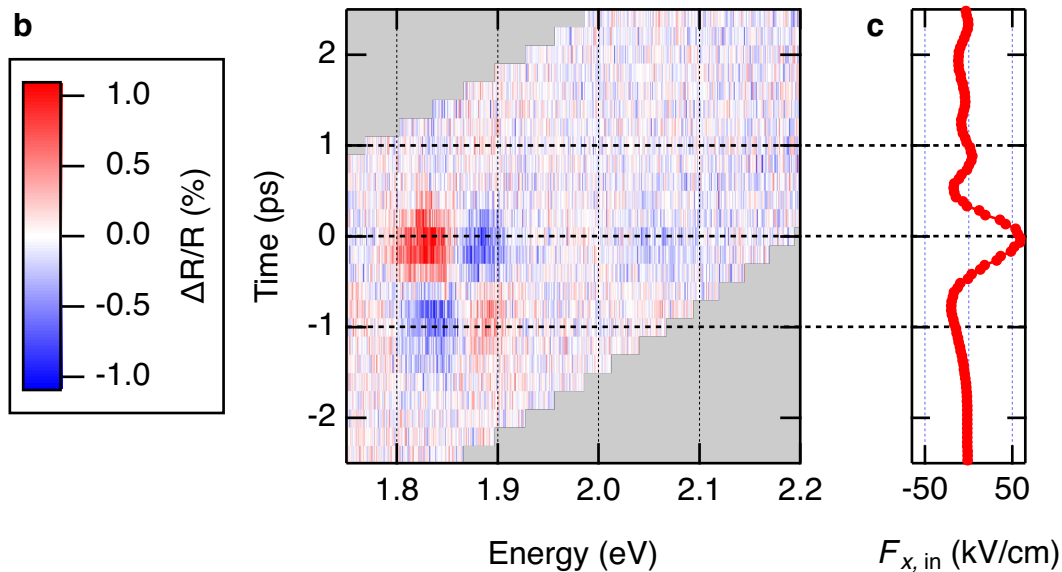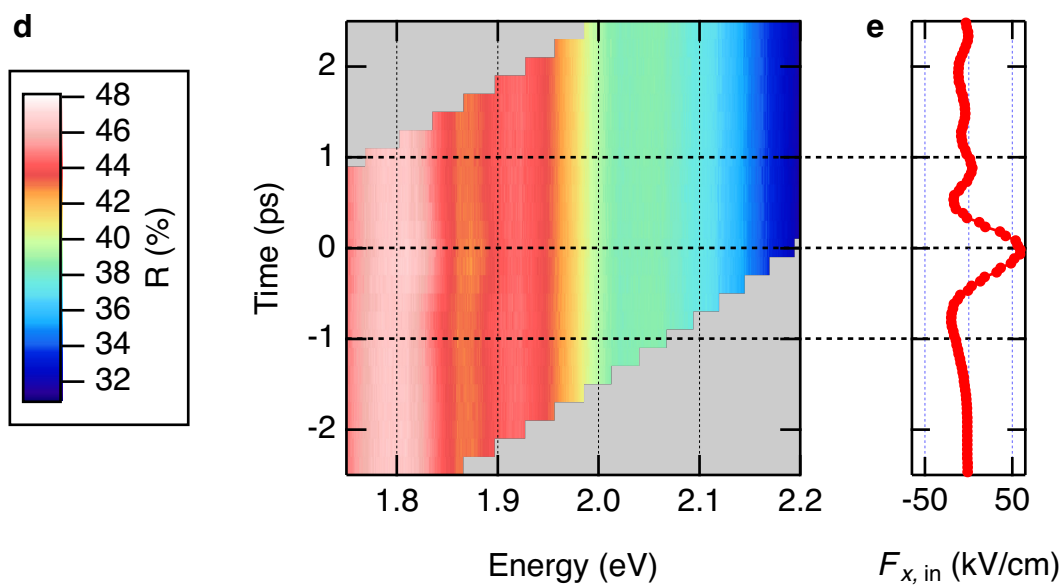

Supplement: Supplementary file 4 — Supplementary Data 1. Figures showing results of all the TPOP measurements and their analysis. For the figure legend of each figure, refer to Supplementary Notes 6. [file 41467_2025_60588_MOESM4_ESM.zip › SupplementaryFigureFiles/deviceII_meas2_0deg/l20240221_tpop1111_summary_measTPOPdata.pdf]

**a**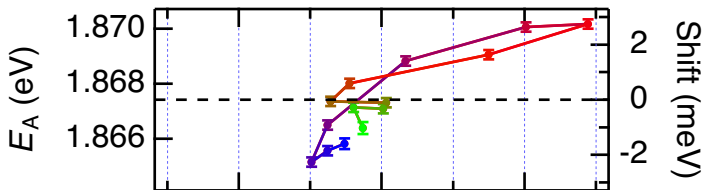**b**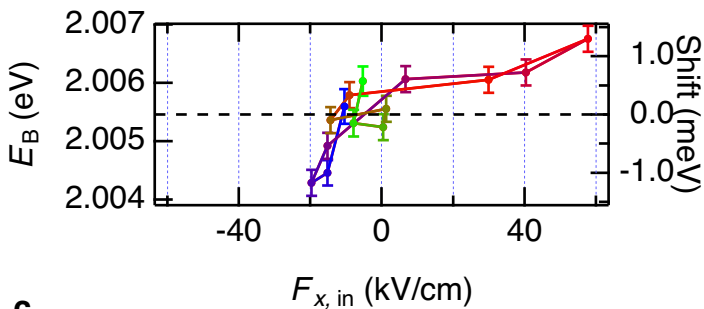**c**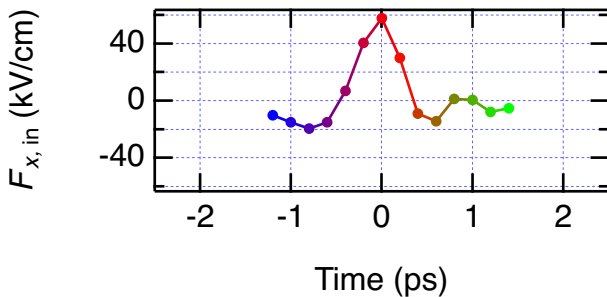

Supplement: Supplementary file 4 — Supplementary Data 1. Figures showing results of all the TPOP measurements and their analysis. For the figure legend of each figure, refer to Supplementary Notes 6. [file 41467_2025_60588_MOESM4_ESM.zip › SupplementaryFigureFiles/deviceII_meas2_0deg/l20240221_tpop1111_REtFit_ParamEshiftVsETHz.pdf]

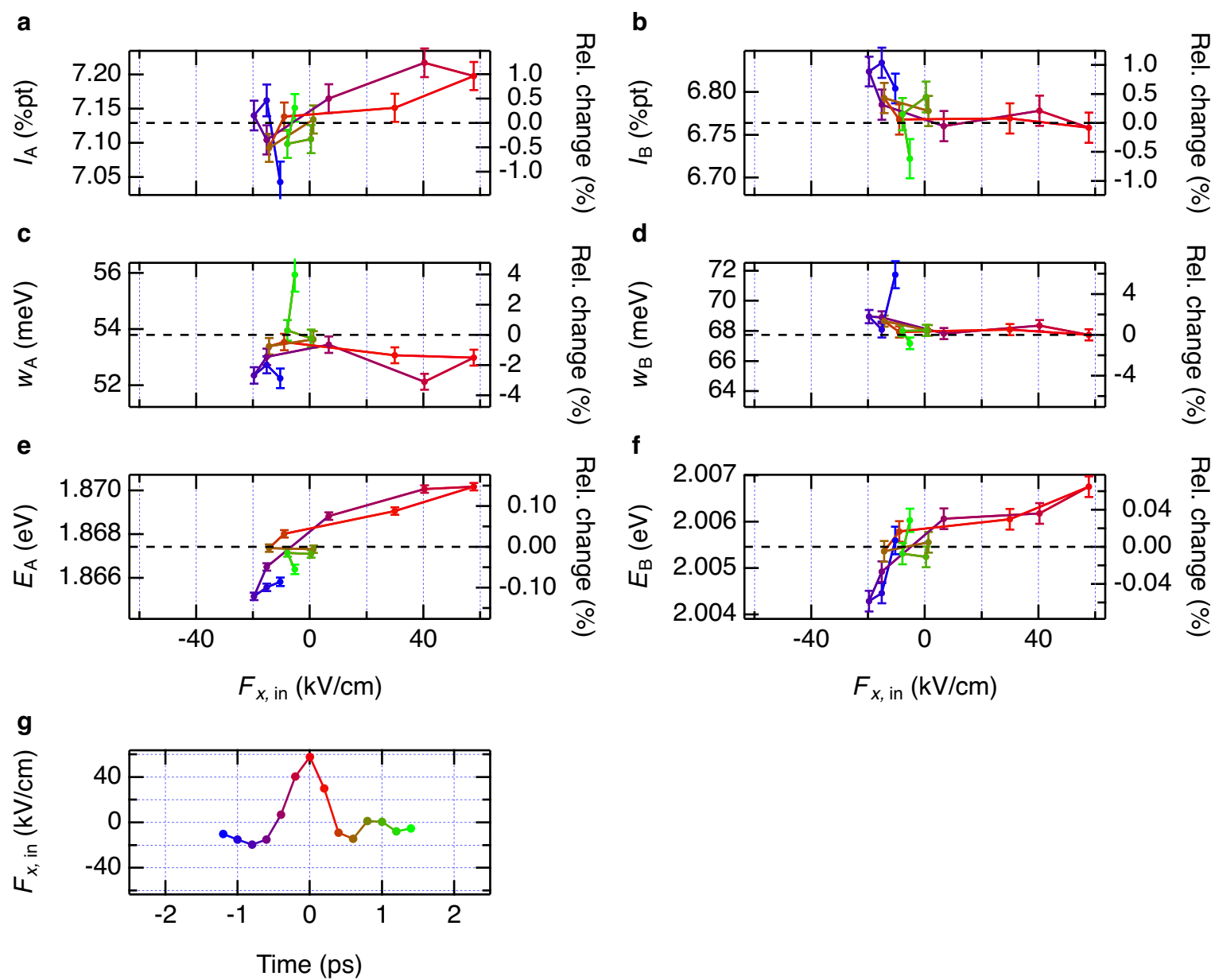

Supplement: Supplementary file 4 — Supplementary Data 1. Figures showing results of all the TPOP measurements and their analysis. For the figure legend of each figure, refer to Supplementary Notes 6. [file 41467_2025_60588_MOESM4_ESM.zip › SupplementaryFigureFiles/deviceII_meas2_0deg/l20240221_tpop1111_REtFit_ParamVsETHz.pdf]

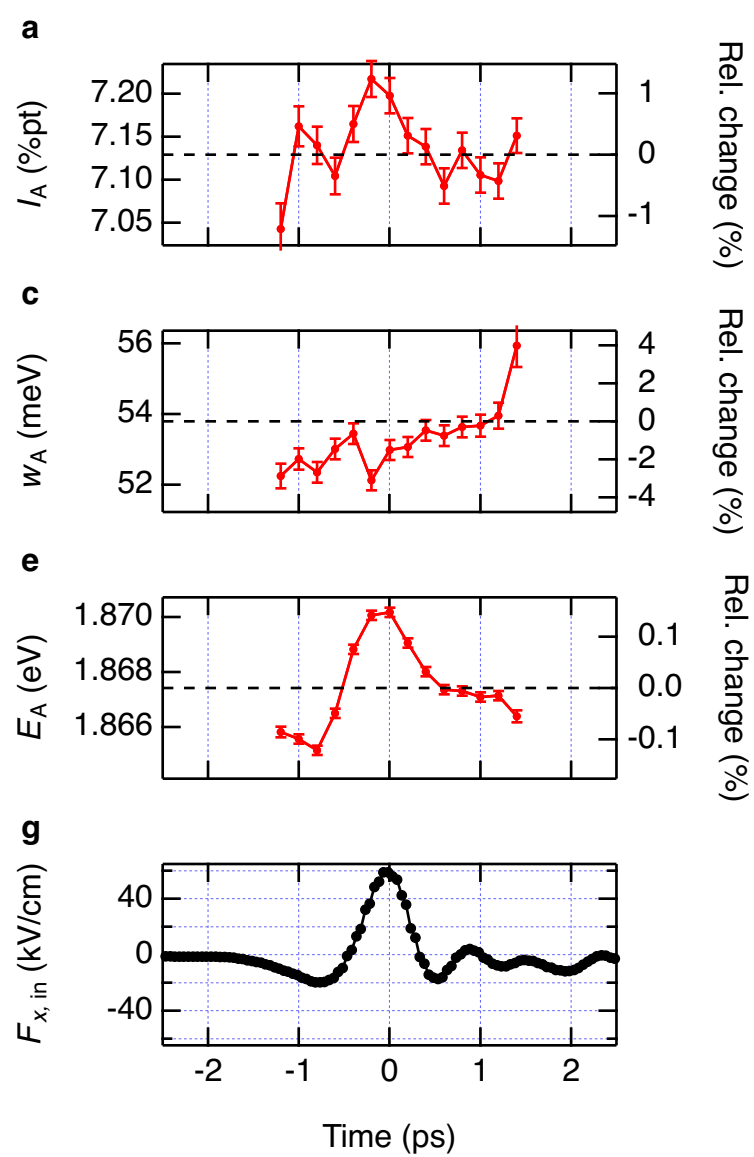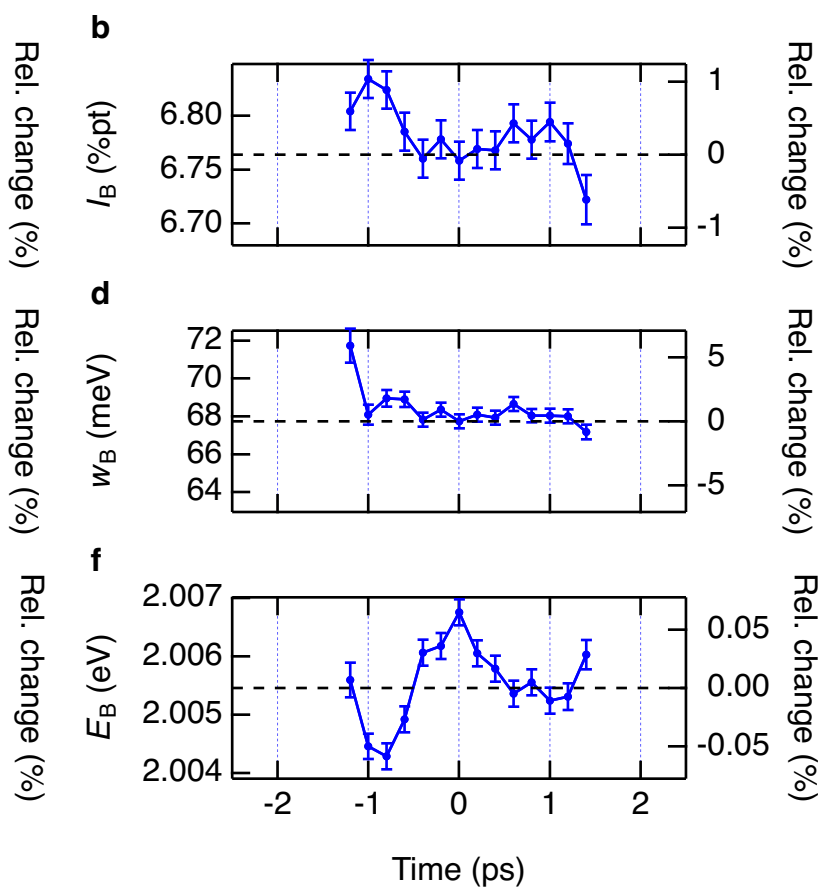

Supplement: Supplementary file 4 — Supplementary Data 1. Figures showing results of all the TPOP measurements and their analysis. For the figure legend of each figure, refer to Supplementary Notes 6. [file 41467_2025_60588_MOESM4_ESM.zip › SupplementaryFigureFiles/deviceII_meas2_0deg/l20240221_tpop1111_REtFit_ParamVsTime.pdf]

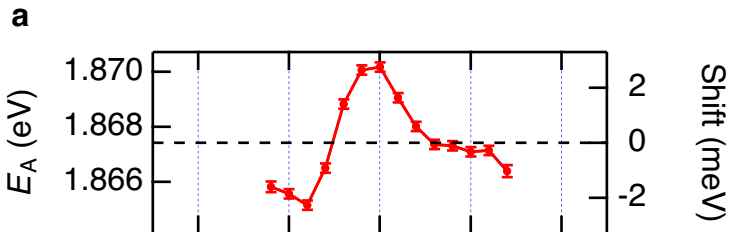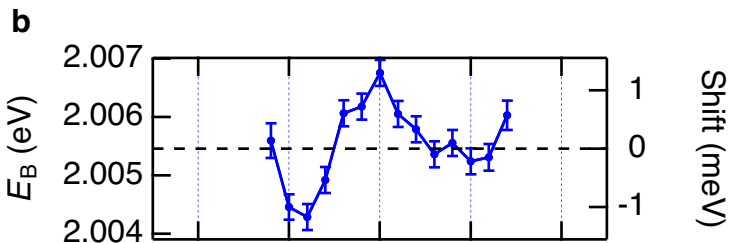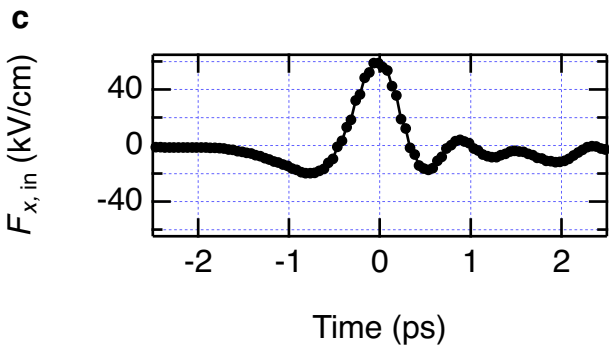

Supplement: Supplementary file 4 — Supplementary Data 1. Figures showing results of all the TPOP measurements and their analysis. For the figure legend of each figure, refer to Supplementary Notes 6. [file 41467_2025_60588_MOESM4_ESM.zip › SupplementaryFigureFiles/deviceII_meas2_0deg/l20240221_tpop1111_REtFit_ParamEshiftVsTime.pdf]

**a**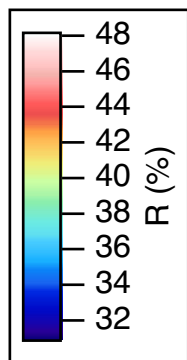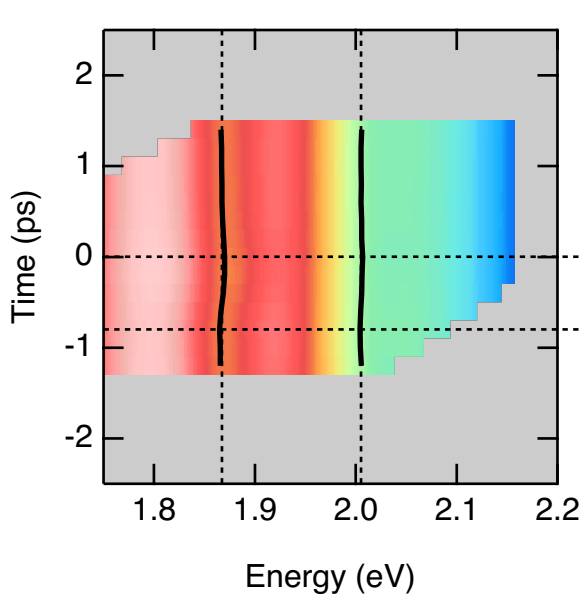**b**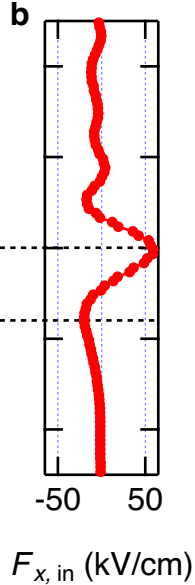**c**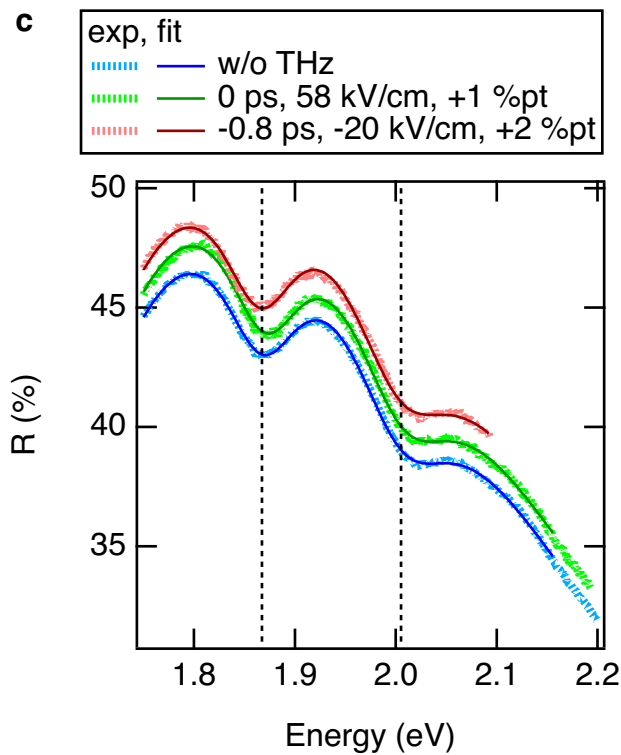

Supplement: Supplementary file 4 — Supplementary Data 1. Figures showing results of all the TPOP measurements and their analysis. For the figure legend of each figure, refer to Supplementary Notes 6. [file 41467_2025_60588_MOESM4_ESM.zip › SupplementaryFigureFiles/deviceII_meas2_0deg/l20240221_tpop1111_REtFit_and_EOS.pdf]

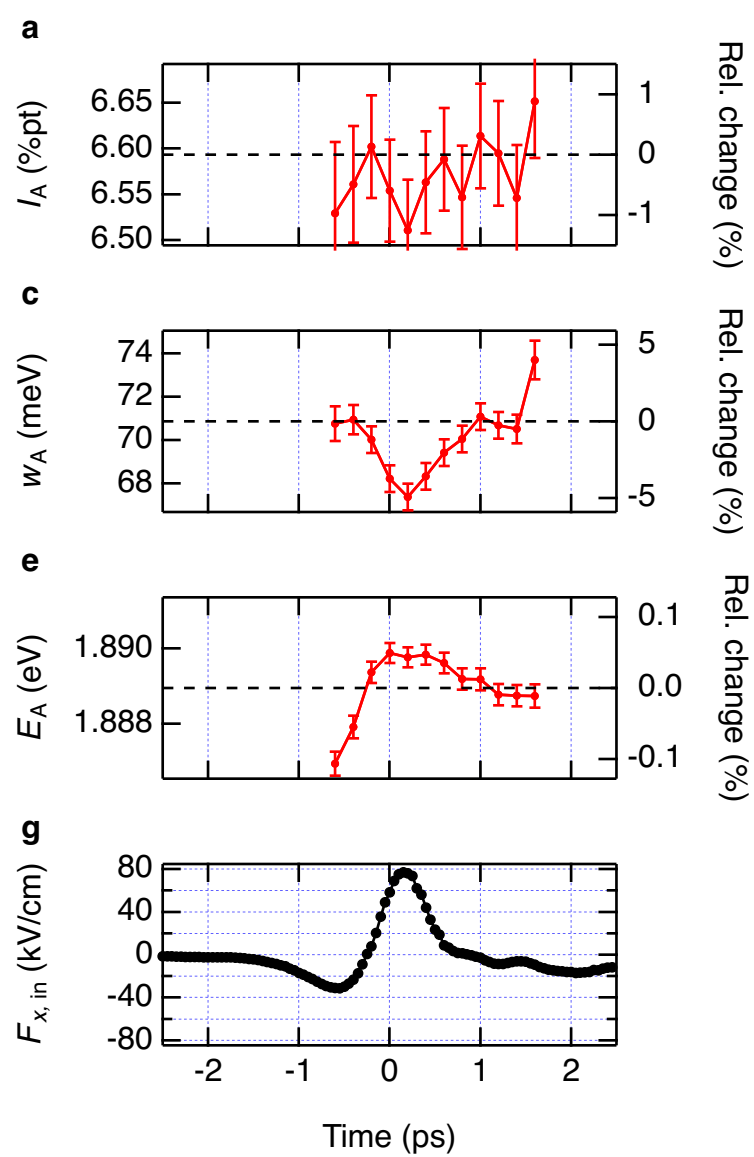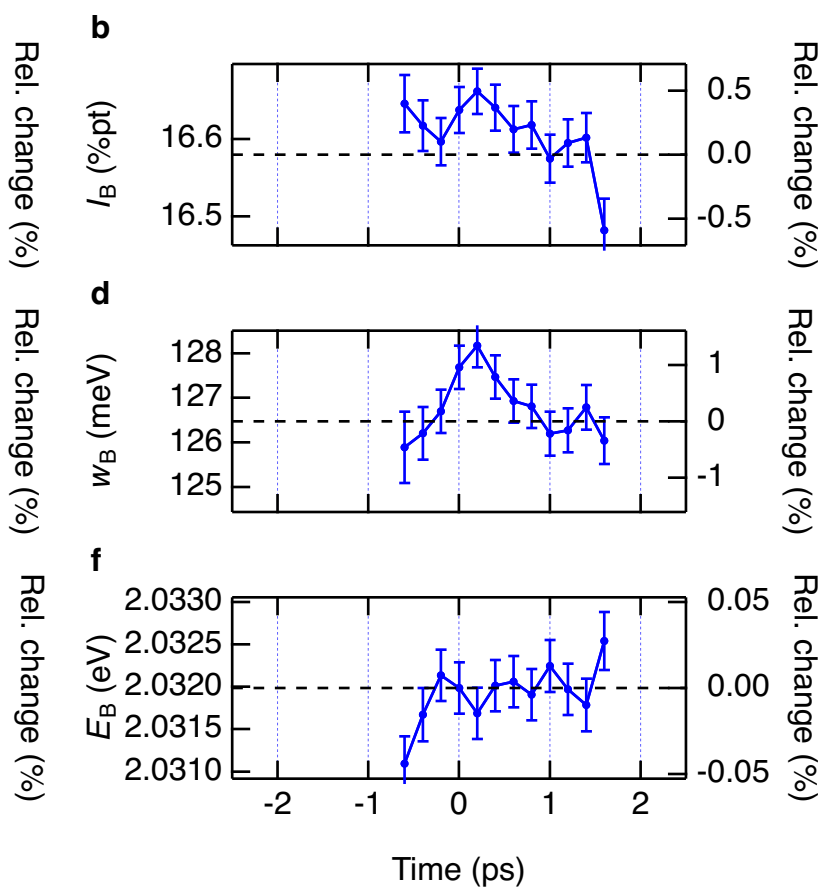

Supplement: Supplementary file 4 — Supplementary Data 1. Figures showing results of all the TPOP measurements and their analysis. For the figure legend of each figure, refer to Supplementary Notes 6. [file 41467_2025_60588_MOESM4_ESM.zip › SupplementaryFigureFiles/deviceIII_meas1_0deg/l20240222_tpop12_REtFit_ParamVsTime.pdf]

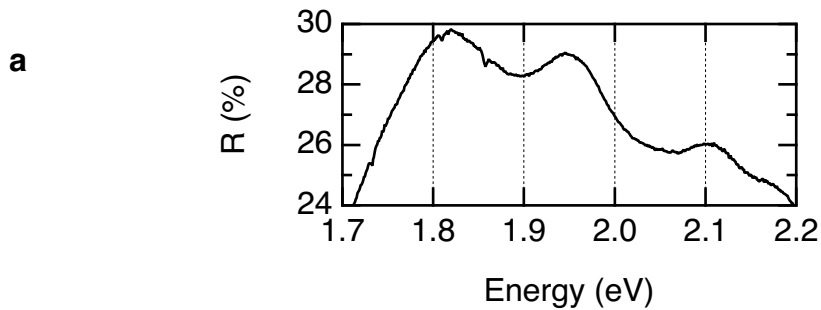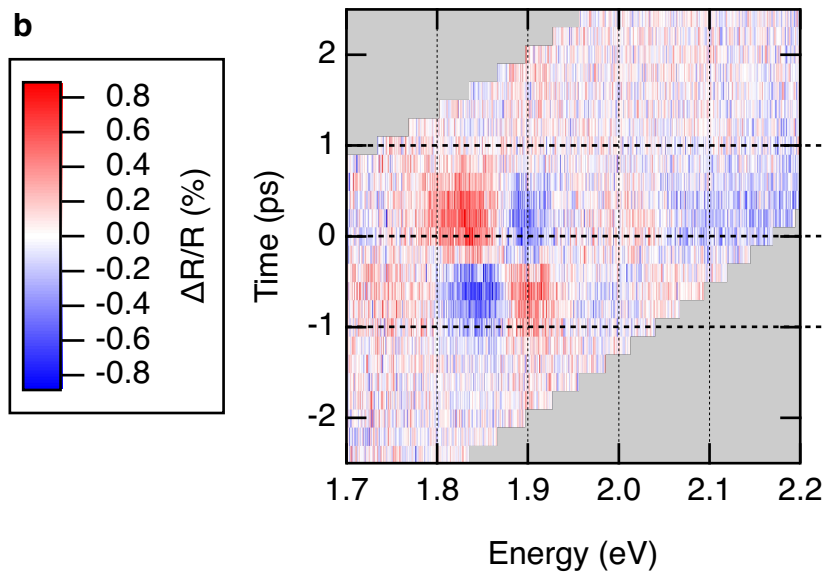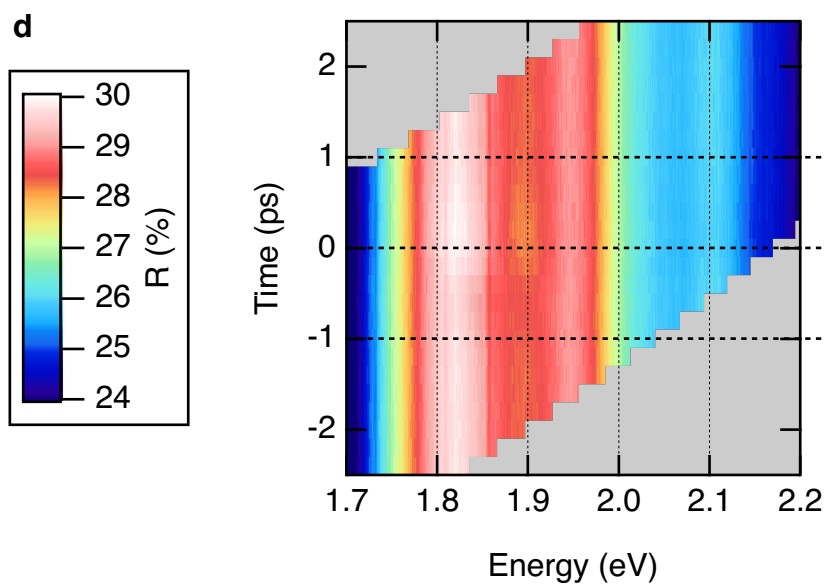

$F_{x, \text{in}}$  (kV/cm)

$F_{x, \text{in}}$  (kV/cm)

Supplement: Supplementary file 4 — Supplementary Data 1. Figures showing results of all the TPOP measurements and their analysis. For the figure legend of each figure, refer to Supplementary Notes 6. [file 41467_2025_60588_MOESM4_ESM.zip › SupplementaryFigureFiles/deviceIII_meas1_0deg/l20240222_tpop12_summary_measTPOPdata.pdf]

**a**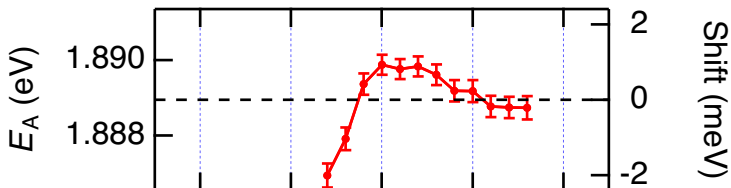**b**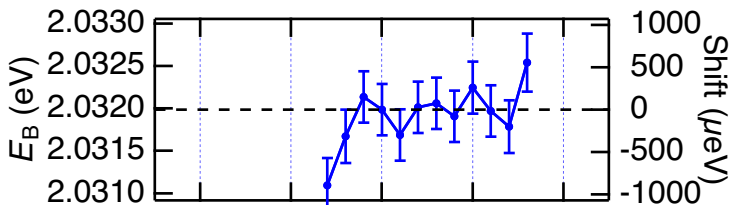**c**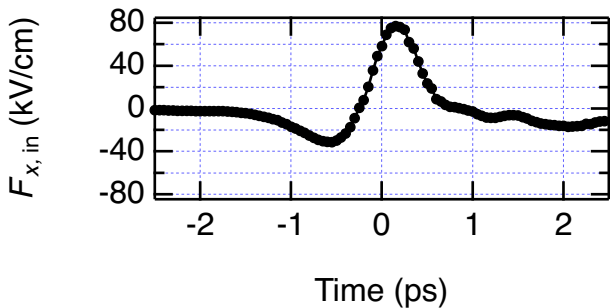

Supplement: Supplementary file 4 — Supplementary Data 1. Figures showing results of all the TPOP measurements and their analysis. For the figure legend of each figure, refer to Supplementary Notes 6. [file 41467_2025_60588_MOESM4_ESM.zip › SupplementaryFigureFiles/deviceIII_meas1_0deg/l20240222_tpop12_REtFit_ParamEshiftVsTime.pdf]

**a**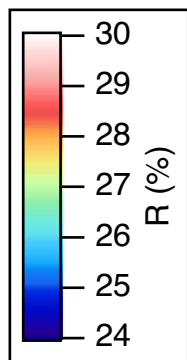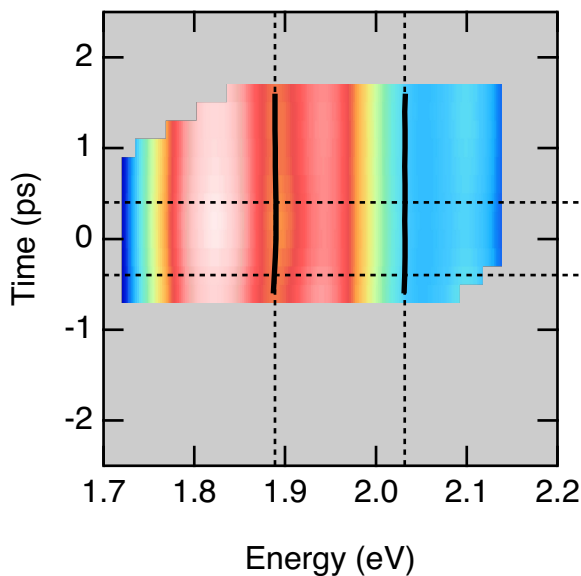**b**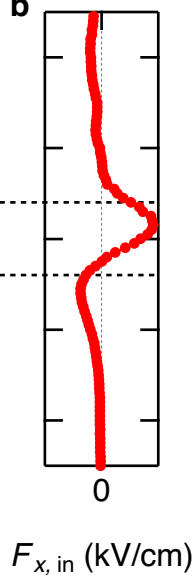**c**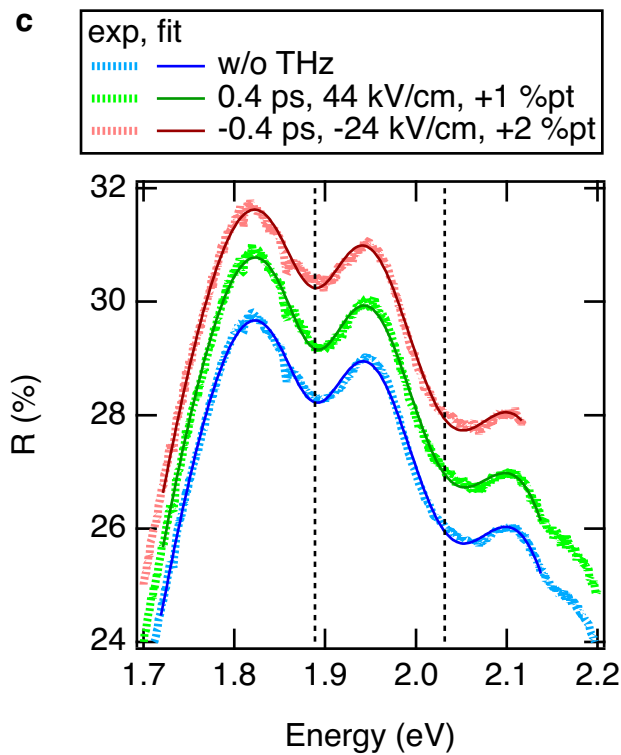

Supplement: Supplementary file 4 — Supplementary Data 1. Figures showing results of all the TPOP measurements and their analysis. For the figure legend of each figure, refer to Supplementary Notes 6. [file 41467_2025_60588_MOESM4_ESM.zip › SupplementaryFigureFiles/deviceIII_meas1_0deg/l20240222_tpop12_REtFit_and_EOS.pdf]

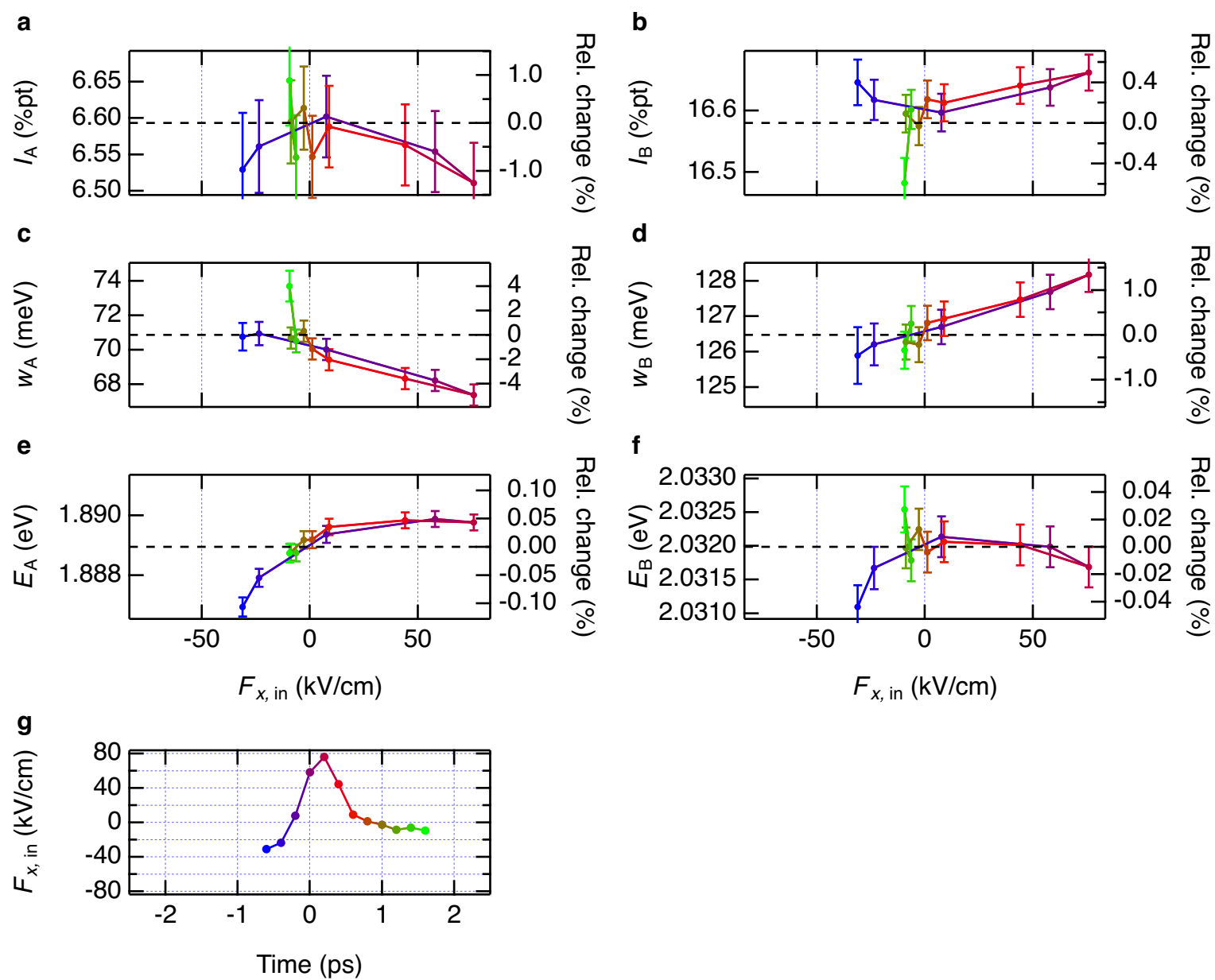

Supplement: Supplementary file 4 — Supplementary Data 1. Figures showing results of all the TPOP measurements and their analysis. For the figure legend of each figure, refer to Supplementary Notes 6. [file 41467_2025_60588_MOESM4_ESM.zip › SupplementaryFigureFiles/deviceIII_meas1_0deg/l20240222_tpop12_REtFit_ParamVsETHz.pdf]

**a**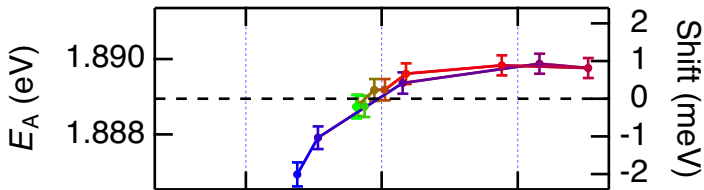**b**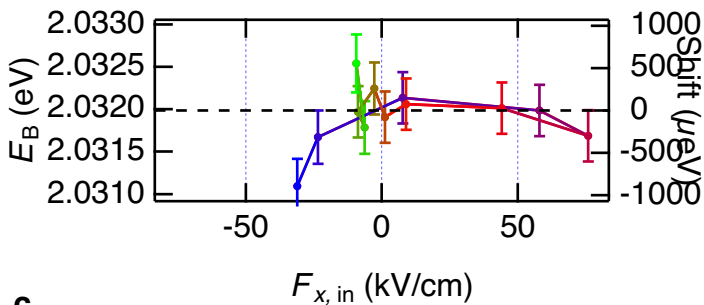**c**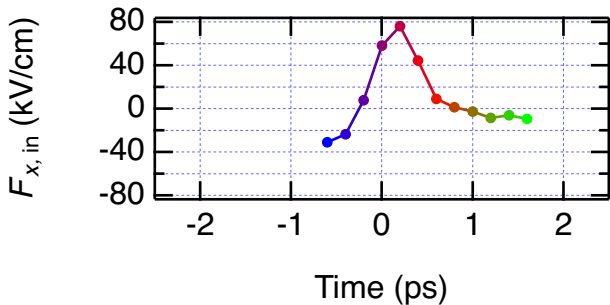

Supplement: Supplementary file 4 — Supplementary Data 1. Figures showing results of all the TPOP measurements and their analysis. For the figure legend of each figure, refer to Supplementary Notes 6. [file 41467_2025_60588_MOESM4_ESM.zip › SupplementaryFigureFiles/deviceIII_meas1_0deg/l20240222_tpop12_REtFit_ParamEshiftVsETHz.pdf]

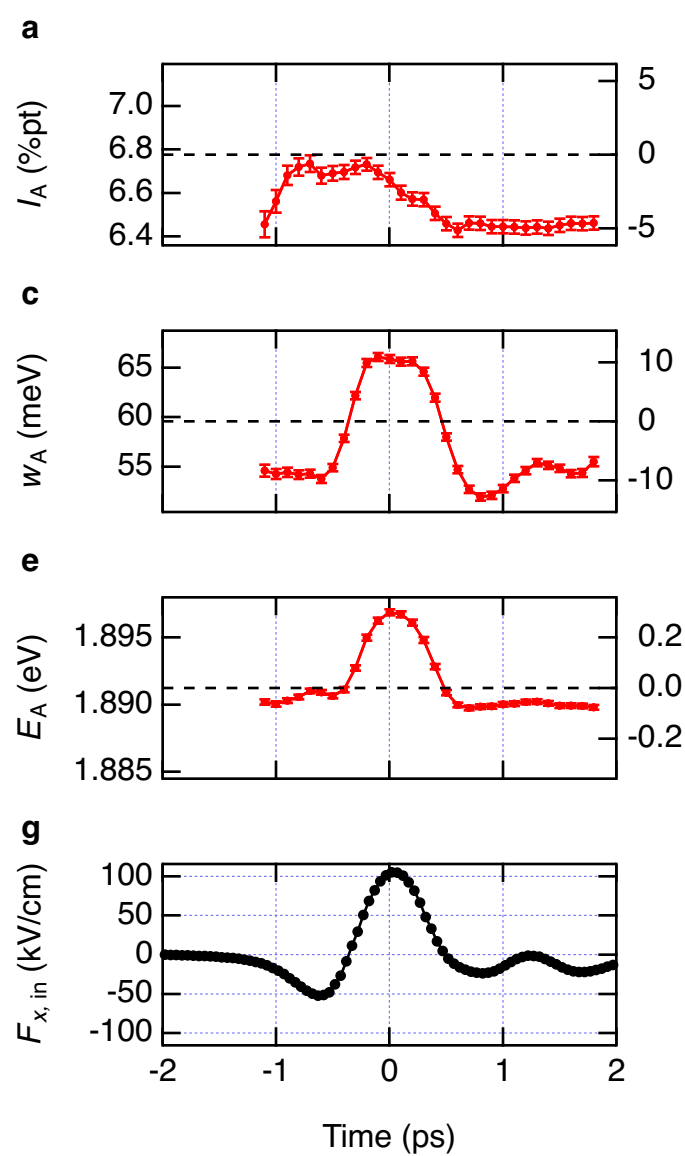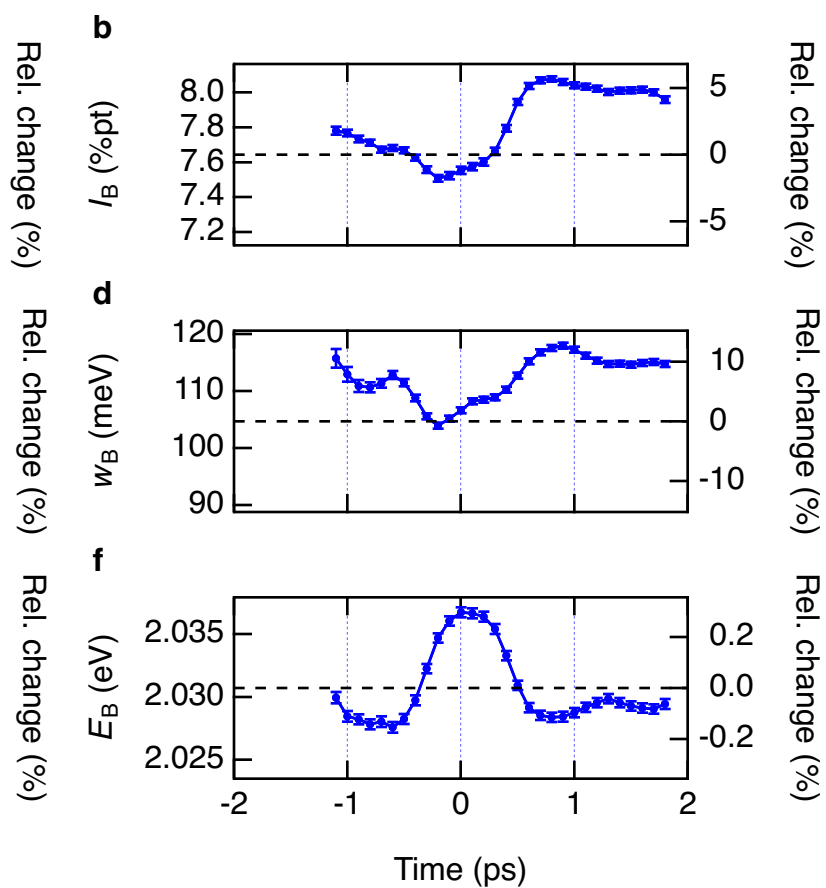

Supplement: Supplementary file 4 — Supplementary Data 1. Figures showing results of all the TPOP measurements and their analysis. For the figure legend of each figure, refer to Supplementary Notes 6. [file 41467_2025_60588_MOESM4_ESM.zip › SupplementaryFigureFiles/deviceI_meas1_0deg/l20230910_exp232_REtFit_ParamVsTime.pdf]

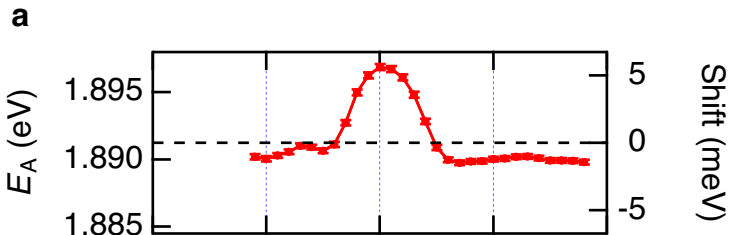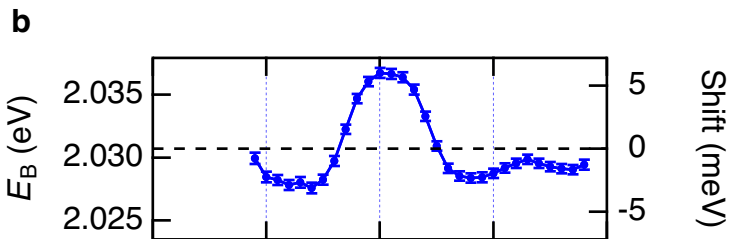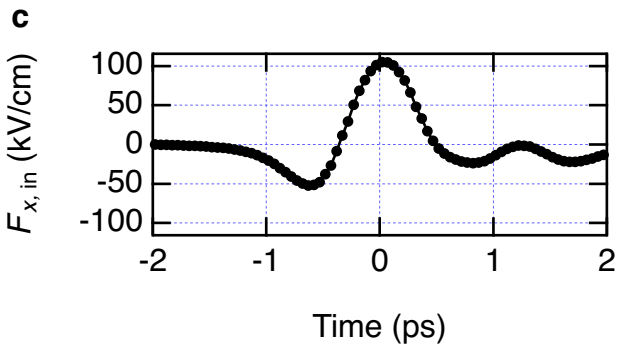

Supplement: Supplementary file 4 — Supplementary Data 1. Figures showing results of all the TPOP measurements and their analysis. For the figure legend of each figure, refer to Supplementary Notes 6. [file 41467_2025_60588_MOESM4_ESM.zip › SupplementaryFigureFiles/deviceI_meas1_0deg/l20230910_exp232_REtFit_ParamEshiftVsTime.pdf]

**a**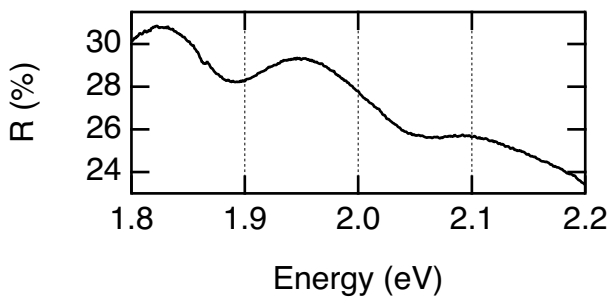**b**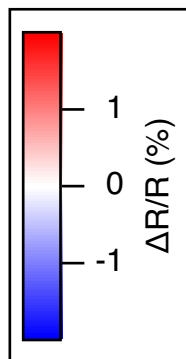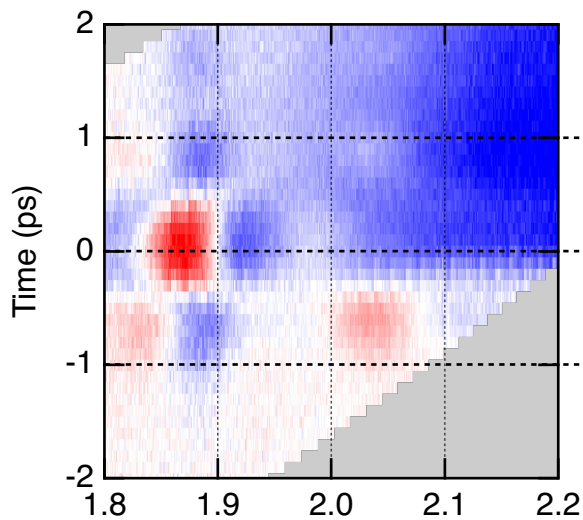**c**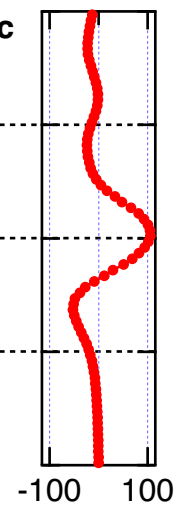**d**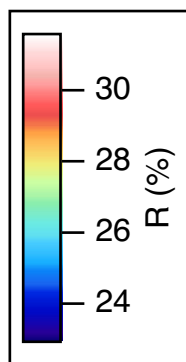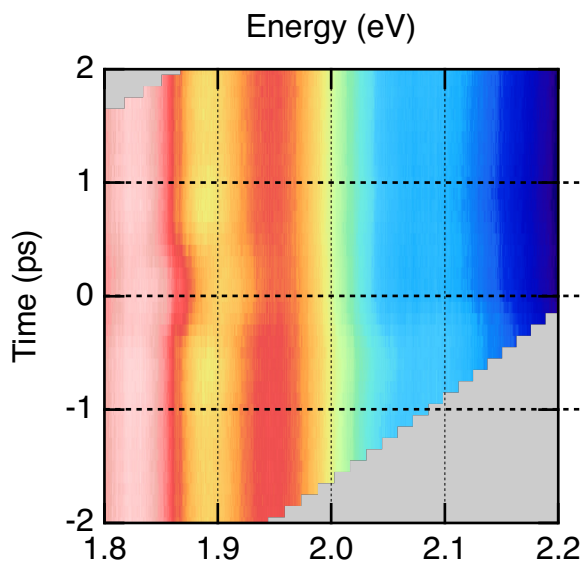**e**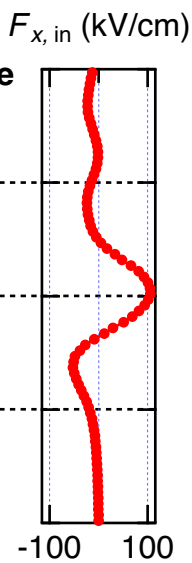

Supplement: Supplementary file 4 — Supplementary Data 1. Figures showing results of all the TPOP measurements and their analysis. For the figure legend of each figure, refer to Supplementary Notes 6. [file 41467_2025_60588_MOESM4_ESM.zip › SupplementaryFigureFiles/deviceI_meas1_0deg/l20230910_exp232_summary_measTPOPdata.pdf]

**a**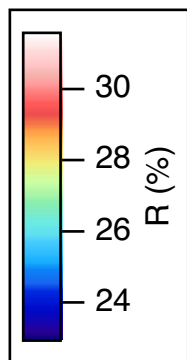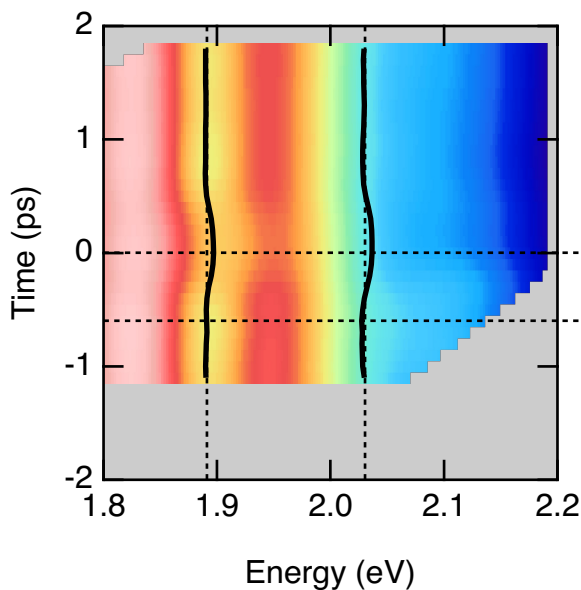**b**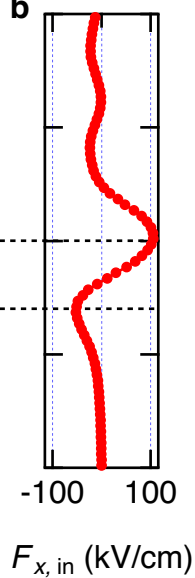**c**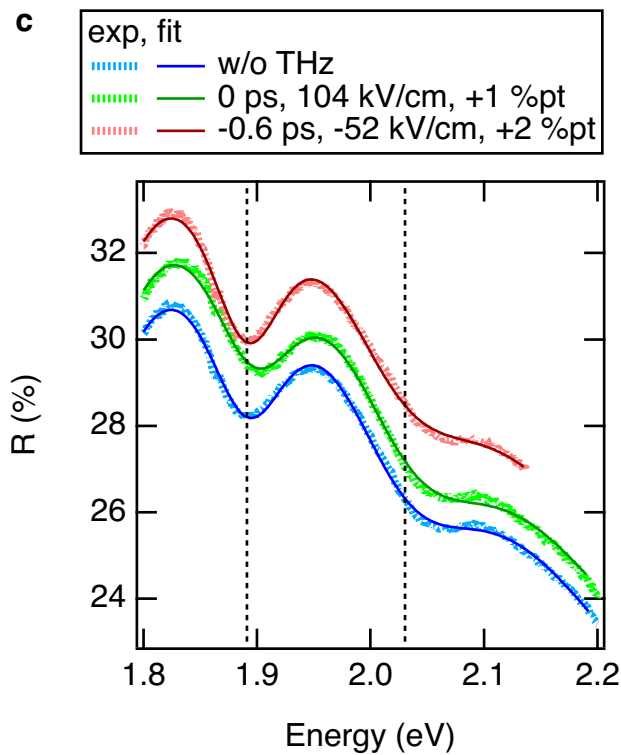

Supplement: Supplementary file 4 — Supplementary Data 1. Figures showing results of all the TPOP measurements and their analysis. For the figure legend of each figure, refer to Supplementary Notes 6. [file 41467_2025_60588_MOESM4_ESM.zip › SupplementaryFigureFiles/deviceI_meas1_0deg/l20230910_exp232_REtFit_and_EOS.pdf]

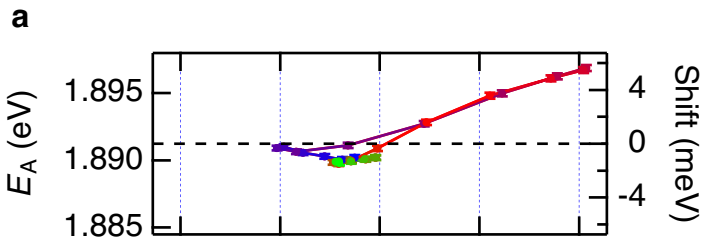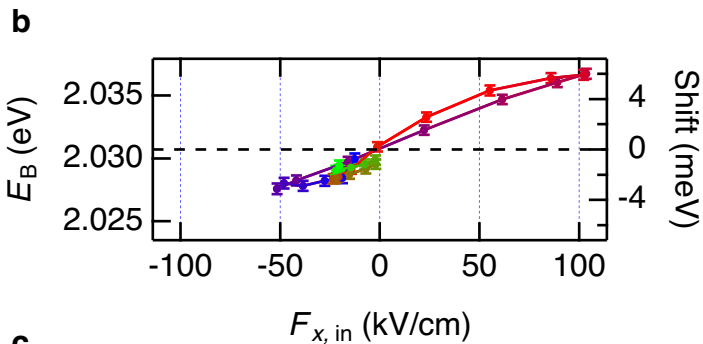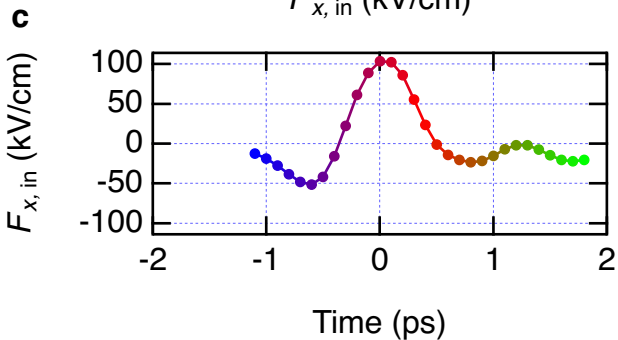

Supplement: Supplementary file 4 — Supplementary Data 1. Figures showing results of all the TPOP measurements and their analysis. For the figure legend of each figure, refer to Supplementary Notes 6. [file 41467_2025_60588_MOESM4_ESM.zip › SupplementaryFigureFiles/deviceI_meas1_0deg/l20230910_exp232_REtFit_ParamEshiftVsETHz.pdf]

Rel. change (%)

Rel. change (%)

Rel. change (%)

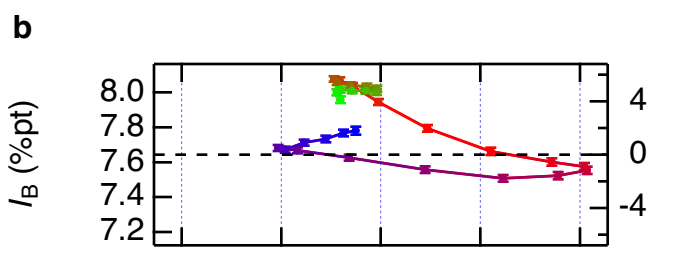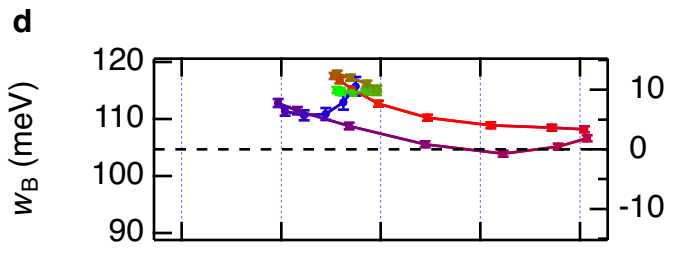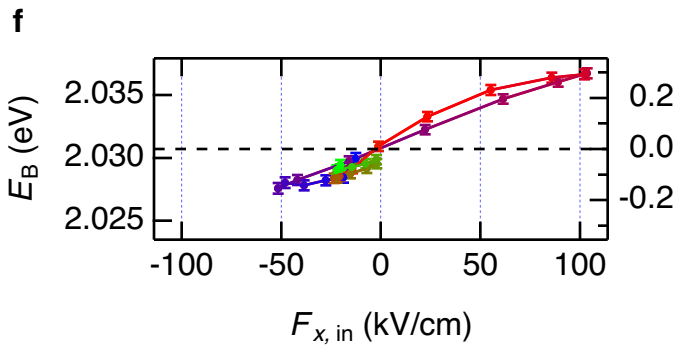

Rel. change (%)

Rel. change (%)

Rel. change (%)

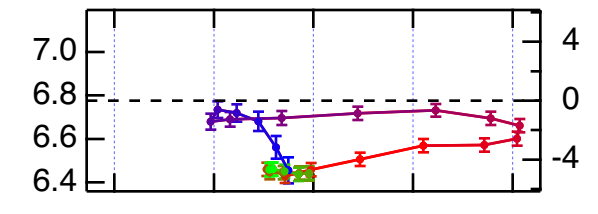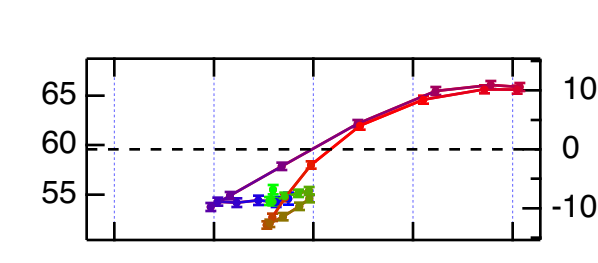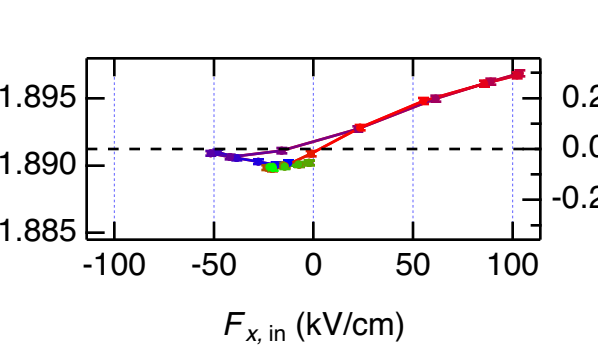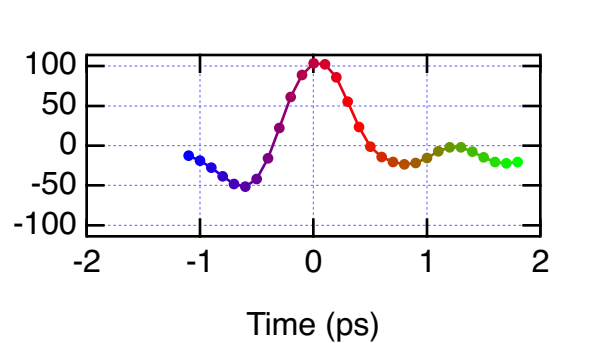

Supplement: Supplementary file 4 — Supplementary Data 1. Figures showing results of all the TPOP measurements and their analysis. For the figure legend of each figure, refer to Supplementary Notes 6. [file 41467_2025_60588_MOESM4_ESM.zip › SupplementaryFigureFiles/deviceI_meas1_0deg/l20230910_exp232_REtFit_ParamVsETHz.pdf]

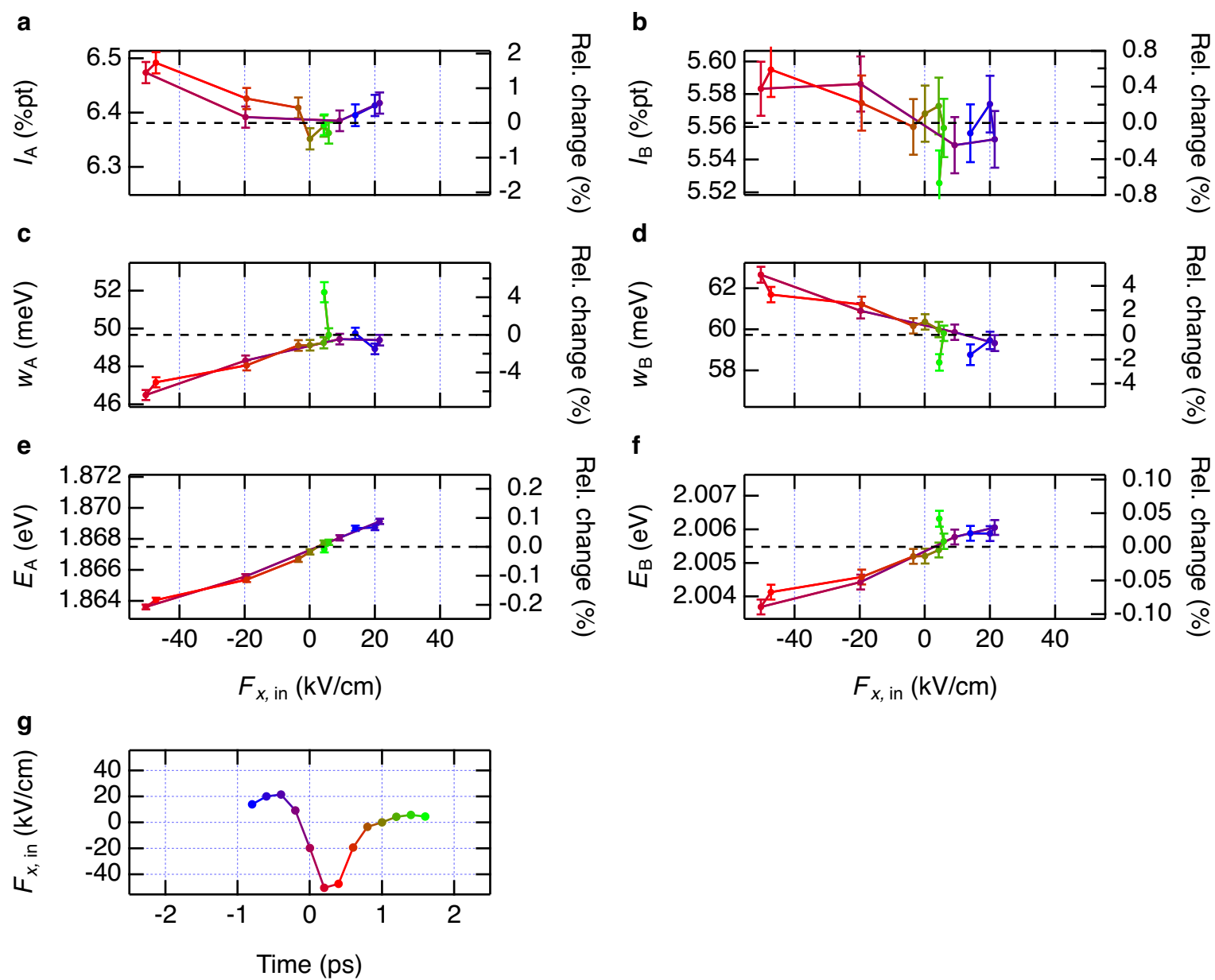

Supplement: Supplementary file 4 — Supplementary Data 1. Figures showing results of all the TPOP measurements and their analysis. For the figure legend of each figure, refer to Supplementary Notes 6. [file 41467_2025_60588_MOESM4_ESM.zip › SupplementaryFigureFiles/deviceII_meas4_180deg/l20240222_tpop42_REtFit_ParamVsETHz.pdf]

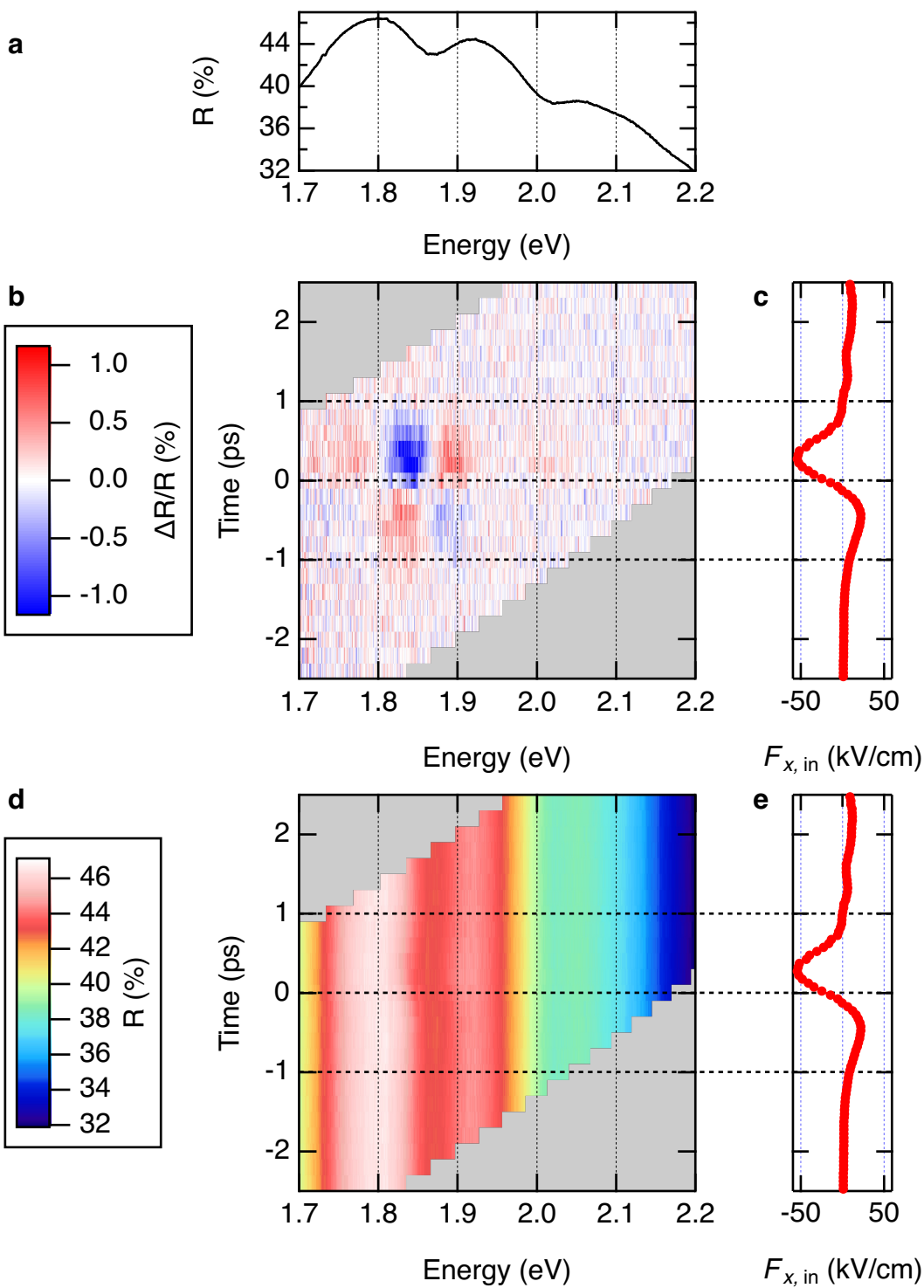

Supplement: Supplementary file 4 — Supplementary Data 1. Figures showing results of all the TPOP measurements and their analysis. For the figure legend of each figure, refer to Supplementary Notes 6. [file 41467_2025_60588_MOESM4_ESM.zip › SupplementaryFigureFiles/deviceII_meas4_180deg/l20240222_tpop42_summary_measTPOPdata.pdf]

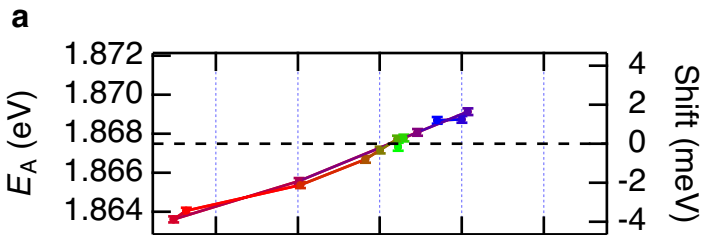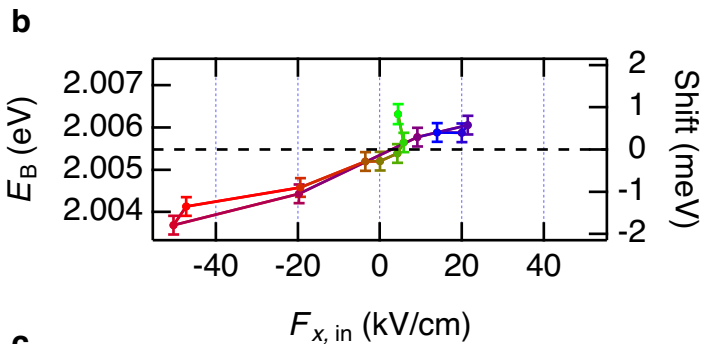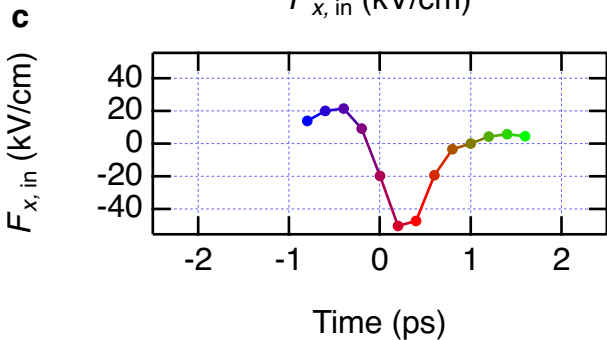

Supplement: Supplementary file 4 — Supplementary Data 1. Figures showing results of all the TPOP measurements and their analysis. For the figure legend of each figure, refer to Supplementary Notes 6. [file 41467_2025_60588_MOESM4_ESM.zip › SupplementaryFigureFiles/deviceII_meas4_180deg/l20240222_tpop42_REtFit_ParamEshiftVsETHz.pdf]

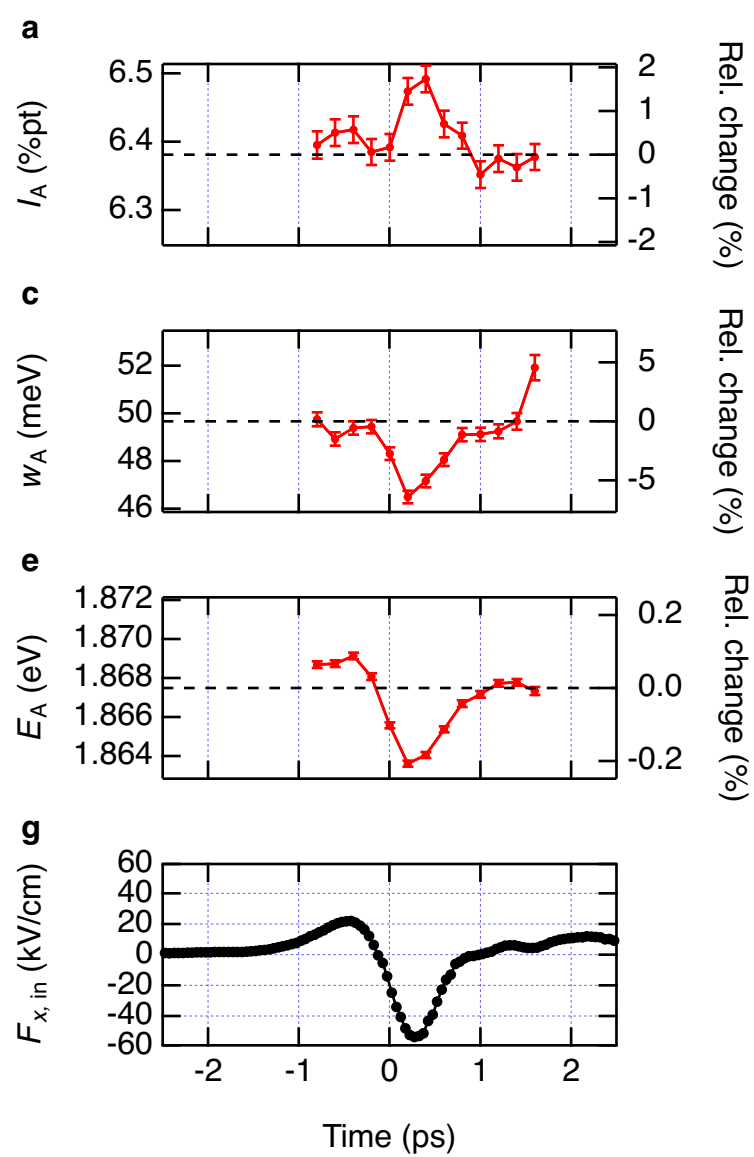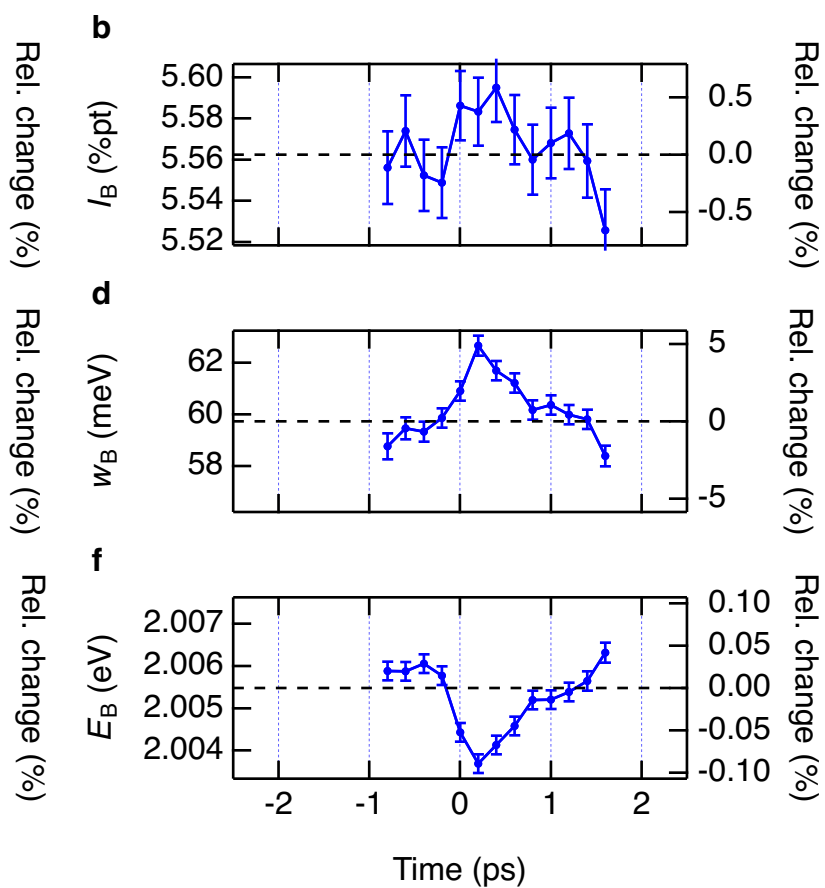

Supplement: Supplementary file 4 — Supplementary Data 1. Figures showing results of all the TPOP measurements and their analysis. For the figure legend of each figure, refer to Supplementary Notes 6. [file 41467_2025_60588_MOESM4_ESM.zip › SupplementaryFigureFiles/deviceII_meas4_180deg/l20240222_tpop42_REtFit_ParamVsTime.pdf]

**a**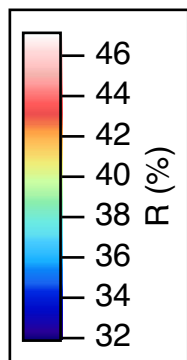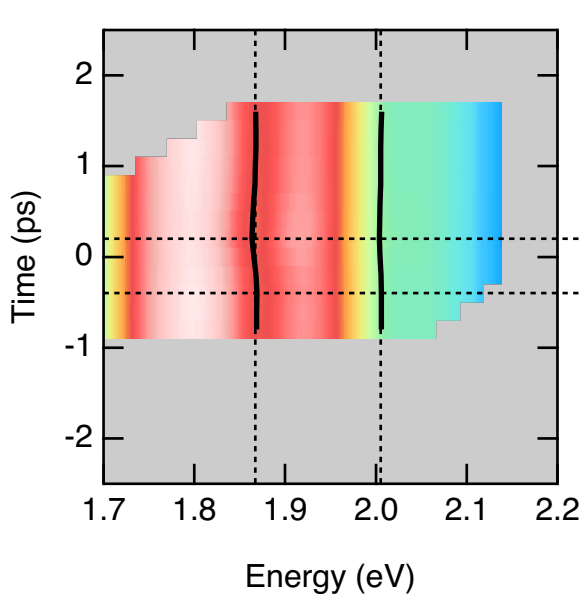**b**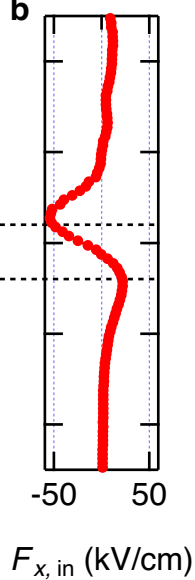**c**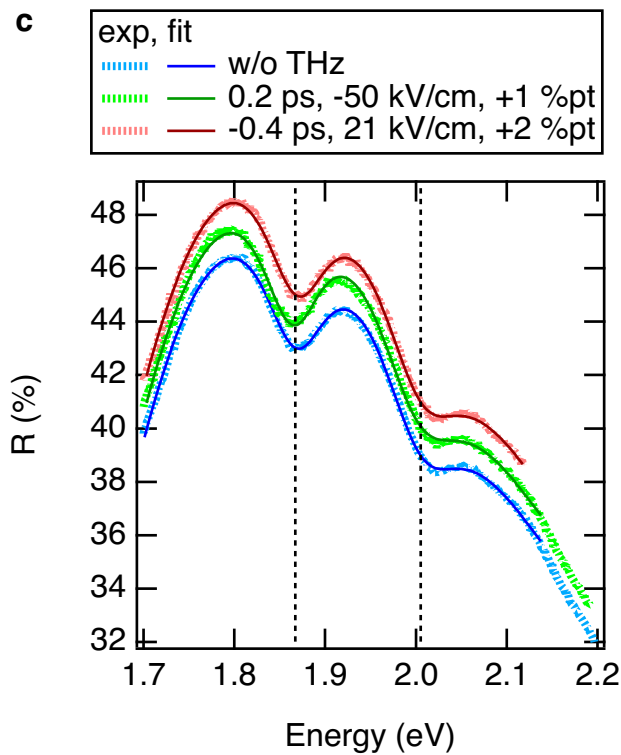

Supplement: Supplementary file 4 — Supplementary Data 1. Figures showing results of all the TPOP measurements and their analysis. For the figure legend of each figure, refer to Supplementary Notes 6. [file 41467_2025_60588_MOESM4_ESM.zip › SupplementaryFigureFiles/deviceII_meas4_180deg/l20240222_tpop42_REtFit_and_EOS.pdf]

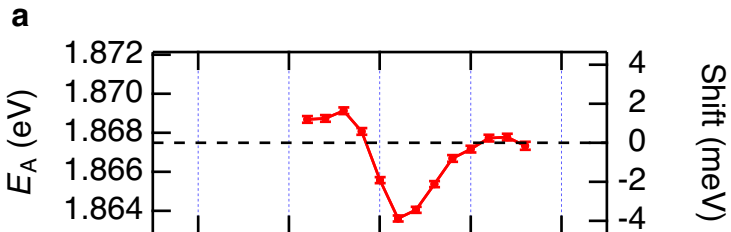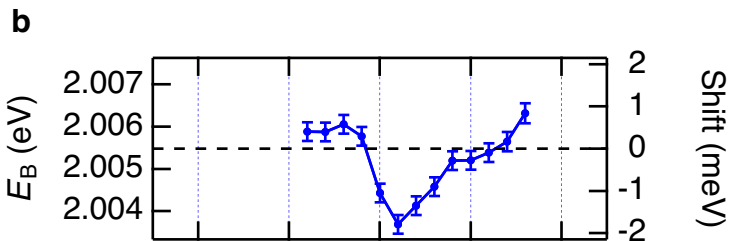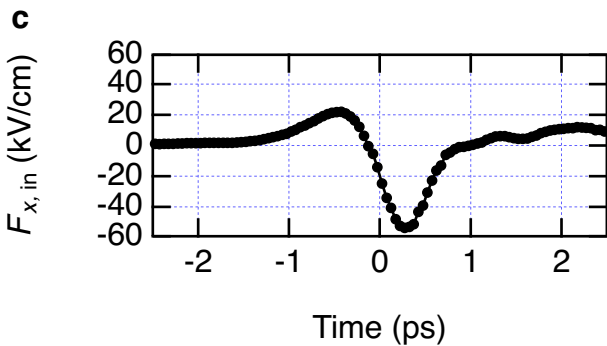

Supplement: Supplementary file 4 — Supplementary Data 1. Figures showing results of all the TPOP measurements and their analysis. For the figure legend of each figure, refer to Supplementary Notes 6. [file 41467_2025_60588_MOESM4_ESM.zip › SupplementaryFigureFiles/deviceII_meas4_180deg/l20240222_tpop42_REtFit_ParamEshiftVsTime.pdf]
